# In-silico design and evaluation of an epitope-based serotype-independent promising vaccine candidate for highly cross-reactive regions of pneumococcal surface protein A

**DOI:** 10.1186/s12967-022-03864-z

**Published:** 2023-01-10

**Authors:** Elnaz Afshari, Reza Ahangari Cohan, Fattah Sotoodehnejadnematalahi, Seyed Fazlollah Mousavi

**Affiliations:** 1 Department of Biology, Science and Research Branch, Islamic Azad University, Tehran, Iran; 2 Department of Nanobiotechnology, New Technologies Research Group, Pasteur Institute of Iran, Tehran, Iran; 3 Department of Microbiology, Pasteur Institute of Iran, 69 Pasteur Ave., Tehran, 13164 Iran

**Keywords:** Clade-defining region, Cross-reactivity, PspA, Serotype-independent vaccine, *Streptococcus pneumoniae*, Immunoinformatics

## Abstract

**Background:**

The pathogenicity of pneumococcus with high morbidity, mortality, and multi-drug resistance patterns has been increasing. The limited coverage of the licensed polysaccharide-based vaccines and the replacement of the non-vaccine serotypes are the main reasons for producing a successful serotype-independent vaccine. Pneumococcal surface protein A (PspA) is an extremely important virulence factor and an interesting candidate for conserved protein-based pneumococcal vaccine classified into two prominent families containing five clades. PspA family-elicited immunity is clade-dependent, and the level of the PspA cross-reactivity is restricted to the same family.

**Methods:**

To cover and overcome the clade-dependent immunity of the PspAs in this study, we designed and tested a PspA_1-5c+p_ vaccine candidate composed of the highest immunodominant coverage of B- and T-cell epitope truncated domain of each clade focusing on two cross-reactive B and C regions of the PspAs. The antigenicity, toxicity, physicochemical properties, 3D structure prediction, stability and flexibility of the designed protein using molecular dynamic (MD) simulation, molecular docking of the construct withHLADRB1*(01:01) and human lactoferrin N-lop, and immune simulation were assessed using immunoinformatics tools. In the experimental section, after intraperitoneal immunization of the mice with Alum adjuvanted recombinant PspA_1-5c+p_, we evaluated the immune response, cross-reactivity, and functionality of the Anti-PspA_1-5c+p_ antibody using ELISA, Opsonophagocytic killing activity, and serum bactericidal assay.

**Results:**

For the first time, this work suggested a novel PspA-based vaccine candidate using immunoinformatics tools. The designed PspA_1-5c+p_ protein is predicted to be highly antigenic, non-toxic, soluble, stable with low flexibility in MD simulation, and able to stimulate both humoral and cellular immune responses. The designed protein also could interact strongly with HLADRB1*(01:01) and human lactoferrin N-lop in the docking study. Our immunoinformatics predictions were validated using experimental data. Results showed that the anti-PspA_1-5c+p_ IgG not only had a high titer with strong and same cross-reactivity coverage against all pneumococcal serotypes used but also had high and effective bioactivity for pneumococcal clearance using complement system and phagocytic cells.

**Conclusion:**

Our findings elucidated the potential application of the PspA_1-5c+p_ vaccine candidate as a serotype-independent pneumococcal vaccine with a strong cross-reactivity feature. Further in-vitro and in-vivo investigations against other PspA clades should be performed to confirm the full protection of the PspA_1-5c+p_ vaccine candidate.

**Supplementary Information:**

The online version contains supplementary material available at 10.1186/s12967-022-03864-z.

## Introduction

*Streptococcus pneumoniae* (pneumococcus) is an opportunistic pathogen and is a major cause of morbidity and mortality worldwide, with more than 98 serotypes based on their polysaccharide capsules [1, 2]. In 2018, the global pneumococcal burden was appraised to be 26.7 occurrences per 1,000 people, resulting in over 1,000,000 deaths [3, 4]. Effective treatment of pneumococcal diseases concerning antibiotic selection is a growing concern because of the increasing multi-drug resistance pattern of pneumococci [5, 6]. For the prevention of pneumococcal diseases, licensed vaccines are based on polysaccharide capsules of the most prevalent pneumococcal serotypes. The limited coverage of the licensed vaccines, broad geographical variation in circulating serotypes, non-vaccine serotype replacement, and the prevalence of non-encapsulated pneumococci from patients with invasive pneumococcal disease (IPD) are key reasons for an attempt to overcome the pneumococcal vaccine limitations and design the novel serotype-independent vaccines [7–11]. Pneumococcal protein-based vaccine (PPV) formulation is a cost-effective and promising candidate for serotype-independent vaccine development [12, 13]; and many pneumococcal conserved cell-surface proteins have already been identified as ideal antigens for PPV in recent years [6, 14, 15].

Pneumococcal surface protein A (PspA) is a very important virulence factor that has been widely studied and is present in all pneumococcal strains [3, 16, 17]. Various active or passive immunization studies using rPspAs demonstrated that animal models were protected against the lethal challenge of pneumococci [7]. Furthermore, the administration of PspA in early human adult clinical trials has been reported [13]. Another study demonstrated that PspA immunization provides more comprehensive protection than Prevnar pneumococcal conjugate vaccine [18].

The N-terminal end of PspA, which is more variable due to mutation accumulation [19], has protection-eliciting epitopes, that have been divided into three regions A, B, and C [20]. The B-region of PspA is serologically variable and forms the basis of classifying PspA into three families with six clades. This region is identified as a clade-defining region (CDR) and comprises two prominent families. Family 1 contains Clades 1 and 2, and Family 2 is made up of Clades 3, 4, and 5. These two families are exhibited in almost 100% of clinical isolates from adult IPD and non-IPD children. Finally, Family 3 is composed of Clade 6, which is extremely rare among pneumococci, and it has been reported that the percentage of Clade 6 in pneumococcal strains is less than 1%. So in many studies, this clade is excluded from the study [20]. Analysis of the CDR sequence showed that the sequences belonging to the same clades demonstrated a sequence identity of ≥ 90% and those of different families ≤ 55% sequence identity [21]. Previous studies have shown high levels of cross-reactivity between different PspA fragments within the B-region of PspA [7, 22]. The C region of PspA is the Proline-Rich Domain (PRD), characterized by the presence of repetitive motifs of proline residues, and highly conserved 22-amino acid immunogenic epitopes called the Non-Proline Block (NPB). Although this region has a partly variable sequence, it is serologically highly cross-reactive and elicits antibodies against the PRD region, which can passively protect mice from lethal pneumococcal disease [21, 23].

**Fig. 1:**
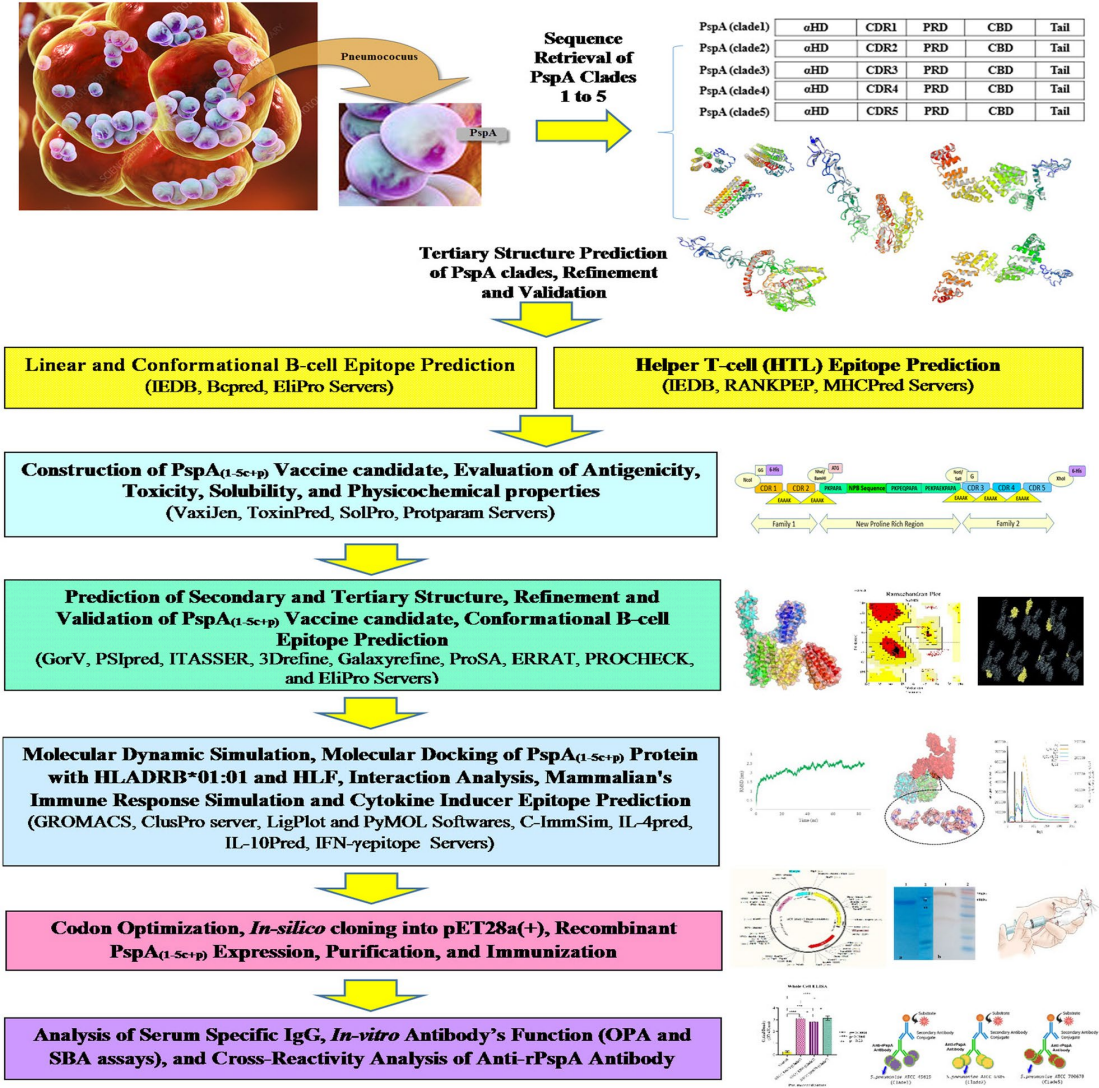
The workflow of the PspA-based vaccine designing against pneumococcus

Increasing evidence strongly proposes that a single protein, especially PspA from one family or clade, will not be sufficient to stimulate protection against all pneumococcal strains [12, 24, 25]. Higher levels of cross-reactivity have been reported within the same family, not between families, and the family-elicited immunity is clade-dependent [7, 22]. Therefore, at least one fragment from each of two prominent families has been considered for PspA-based vaccines to extend protection [22]. Akbari et al. showed that immunization of mice with PspAB1-5 (B region of N-terminal from all PspA clades) led to higher protection than PspA4ABC (A, B, and C regions of PspA Clade 4) in pneumococcal challenges [7]. Other studies showed that the combined vaccine candidate composed of two segments of each PspA family exhibited varying degrees of cross-reactivity and protection. Piao et al. showed that in three constructs of the PspA, including N-terminal and proline-rich regions from PspA families 1 and 2, immunization with PspA2 + 4 and PspA2 + 5 exhibited no protection against pneumococcal challenge with two Clades 1 and 3. Also, the binding capacity of the anti-PspA3 + 2 specific IgG to the surface of pneumococci with PspA Clades 1–4 was high, but not for Clade5. Finally, they concluded that PspA3 + 2 has an advantage over PspA2 + 4 and PspA2 + 5 [7, 25]. Akbari et al. also suggested that all B and C regions of all clades should be used in PspA-based vaccine designs to achieve the full level of cross-reactivity and cross-protection against all pneumococci [7]. Therefore, an essential step for PspA-based vaccine design is to cover and overcome clade-dependent immunity against all pneumococcal strains expressing all PspA families by selecting immunodominant truncated domains of all PspA clades focusing on two cross-reactive B and C-regions. This can be achieved through cost and time-benefit approaches such as immunoinformatics tools in vaccine development. Many studies have reported that immunoinformatics, reverse vaccinomics, or computational immunological approaches are reliable, accurate, quick, and cost-effective methods, with a broad collection of available and powerful tools for epitope-based vaccine design and vaccine development [26–30]. Therefore, the present study is the first attempt to use immunoinformatics tools for epitope mapping analysis of the N-terminal sequence of all five PspA clades. Then we designed and constructed the PspA_1-5c+p_ vaccine candidate and evaluated the PspA_1-5c+p_ protection against pneumococcal infection by immunization of mice with recombinant PspA_1-5c+p_. We also evaluated the cross-reactivity ability of the anti-PspA_1-5c+p_ antibody against pneumococcal strains representing both PspA families and the functional activity of the anti-PspA_1-5c+p_ antibody. The findings suggest the potential use of this vaccine candidate as a novel serotype-independent PspA-based pneumococcal vaccine with a strong cross-reactivity response. The schematic procedure of this research has been shown in Fig. 1.

## Materials and methods

### Sequence retrieval and the structural characteristics

PspA sequence of the pneumococcal strain St 435/96 (Clade1), RX1 (Clade2), EF3296 (Clade3), EF5668 (Clade4), and strain ATCC 6303 (Clade5), which represented five different clade types of the PspA, were retrieved from the National Center for Biotechnology Information (NCBI) at https://www.ncbi.nlm.nih.gov with the accession number of the GenBank AAL92492.1, AAA27018.1, AAF27712.1, AAC62252.1, and AAF27715.1, respectively. To analyze the subcellular localization, transmembrane helices, and signal peptide of the proteins, online servers of CELLO v.2.5 (http://cello.life.nctu.edu.tw/) [31], TMHMM (http://www.cbs.dtu.dk/services/TMHMM-2.0/) [32], and signalP-5.0 (http://www.cbs.dtu.dk/services/SignalP-5.0/) [33] were used, respectively. The antigenicity of the proteins was also analyzed using the Vaxijen-v2 server (http://www.ddg-pharmfac.net/vaxijen/VaxiJen/VaxiJen.html) [34] with a threshold of 0.5.

### Immunoinformatics analysis

#### Potential B-cell epitope prediction

For the prediction of linear B-cell epitopes, antigenicity, surface accessibility, flexibility, β-turn, and hydrophilicity of proteins, the amino acid sequences were analyzed using a collection of methods in the IEDB server (https://www.iedb.org/) [35] according to sequence characteristics of the antigen and BCpred (http://ailab.ist.psu.edu/bcpred/predict.html) [35] server. BCpred server uses support vector machine (SVM) classifiers for linear B-cell epitope prediction [35]. Since the three-dimensional (3D) structural template of PspA proteins did not exist in the Protein Data Bank (PDB) database, the prediction of the 3D structure of each PspA clade was performed using the I-TASSER server as a template-based fragment assembly simulator (https://zhanglab.ccmb.med.umich.edu/I-TASSER/) [36]. After prediction, the best-modeled structure for each clade was selected according to the model’s ranking scores. The acceptable confidence score (towards + 2), template modeling score (toward a score of 1), and a lower Root Mean Square Deviation score (RMSD) as a degree of similarity between the modeled protein and the other were considered. Then the selected model was refined using the Galaxy Refine server (http://galaxy.seoklab.org/cgi-bin/submit.cgi?type=REFINE) [37]. This structure refinement server carries out repeated structural disruptions by reconstruction of the side chains and subsequent overall structural relaxations through molecular dynamics simulation [37]. The final model was validated with a score of similarity to native proteins as a Z-score in the ProSA server (https://prosa.services.came.sbg.ac.at/prosa.php/) [38], distribution of residues in most favored, allowed, and disallowed regions of Ramachandran plot in the PROCHECK server, and quality percentage of structure in the ERRAT from Saves server (https://saves.mbi.ucla.edu/) [39]. Lastly, we used the refined and validated PspA clade 3D structure in the prediction of conformational B-cell epitopes using the Ellipro server (http://tools.iedb.org/ellipro/) [40].

#### Helper T-cell (HTL) epitope prediction

Several servers such as IEDB (https://www.iedb.org/) [41], RANKPEP (http://imed.med.ucm.es/Tools/rankpep.html) [42], and MHCPred (http://www.ddg-pharmfac.net/mhcpred/MHCPred/) [43] servers were used for predicting MHC-II binding epitopes. The prediction of (15 mer) peptide binding affinities to eight human HLA-II super type alleles HLA-DR B* (01:01-03:01-04:01-07:01-08:01-11:01-13:01-15:01), as well as mouse MHC-II H2 alleles I-Ad, I-Ab, and I-Ed, were applied to have strong T cell responses in human and BALB/c mice [44–47]. RANKPEP server utilizes protein sequence/s or sequence alignments by Position Specific Scoring Matrices (PSSMs) to predict peptide binders to MHCII molecules [42], and the MHCPred server employs the additive method for prediction of the MHCII binding affinity of molecules and to the Transporter associated with Processing (TAP) [43]. The predicted epitope sequences were analyzed for the presence or absence of similarity with the human proteome using the PIR peptide matching program (https://research.bioinformatics.udel.edu/peptidematch/index.jsp) [48].

### Construction of chimeric PspA_1-5c+p_

The truncated domain from five PspA clades composing overlapping immunodominant B and T-cell epitopes in CDR sequence and the motif sequences from each of three PRD groups with the highly conserved and immunogenic region of NPB [21] were used in the formulation of chimeric PspA_1-5c+p_ construct. The above regions were assessed to fuse as a multi-component vaccine using an EAAAK linker [49] to achieve the best three-dimensional structure with the least interaction between each part. The antigenicity and toxicity were predicted for the chimeric PspA_1-5c+p_ construct using Vaxijen-v2 [25] with a threshold of 0.5 and ToxinPred servers (https://webs.iiitd.edu.in/raghava/toxinpred/index.html) [50], respectively.

### Physicochemical properties and solubility prediction

The Protparam online server (https://web.expasy.org/protparam/) [51] was used to evaluate the physicochemical properties of the PspA_1-5c+p_ construct. SOLpro server (http://scratch.proteomics.ics.uci.edu/explanation.html#SOLpro/) was applied to predict the solubility of PspA_1-5c+p_ construct upon overexpression in *E.coli* [51, 52].

### Prediction of secondary and tertiary structures

The prediction of the PspA_1-5c+p_ secondary structure was performed by GORV (https://npsa-prabi.ibcp.fr/cgi-bin/npsa_automat.pl?page=/NPSA/npsa_gor4.html/), PSIPRED 4.0 (http://bioinf.cs.ucl.ac.uk/psipred/) [47], and RaptorX Property web servers (http://raptorx.uchicago.edu/StructurePropertyPred/predict/). Using I-TASSER (https://zhanggroup.org/I-TASSER/) server [36], the tertiary structure of the PspA_1-5c+p_ was built. PyMol software v.2.5 was applied to visualize a high-quality image of the predicted model [53]. The best-modeled structure of the PspA_1-5c+p_ construct was selected according to the C-score, TM-score, and RMSD-score.

### Tertiary structure refinement and validation

The selected 3D structure model of the PspA_1-5c+p_ was refined using the 3DRefine (http://sysbio.rnet.missouri.edu/3Drefine/index.html/) [54] and then Galaxy Refine (http://galaxy.seoklab.org/cgi-bin/submit.cgi?type=REFINE/) [37]. 3Drefine has refined the 3D model to optimize the hydrogen-bonding network and minimize the atomic energy of the model. Galaxy Refine server refined the model by molecular dynamics simulation. The final model of the PspA_1-5c+p_ was validated with ProSA (https://prosa.services.came.sbg.ac.at/prosa.php/) [38], PROCHECK, and ERRAT from the Saves server (https://saves.mbi.ucla.edu/) [39], to recognize the errors in the generated 3D model. We also used the refined and validated 3D structure of the PspA_1-5c+p_ to predict conformational B-cell epitopes using the Ellipro server (http://tools.iedb.org/ellipro/) [55].

### Molecular dynamic simulation

The molecular dynamic (MD) simulation was carried out to study the stability of the PspA_1-5c+p_ protein model using GROMACS 2018 [56, 57]. The structure was simulated for 85 ns with optimized potential for liquid simulations (OPLS) force field in a 10 A° cubic simulation box of the simple point charge (SPC) as a water molecule type. The neutralization of the system charge was done by adding Na^+^‏ and Cl^−^ ions. Then simulation system was minimized by the steepest descent minimization integrator and the maximum force was less than 100 kJ.mol^−1^ nm^−1^ with 5000 minimization steps. Afterward, the equilibration of the simulation system was performed with NVT (constant number of particles, volume, and temperature) and NPT (constant number of particles, pressure, and temperature with a leap-frog integrator for 100 picoseconds (ps). All bond constraints were organized with the linear constraint solver (LINCS) algorithm. The electrostatic interaction of the particle mesh Ewald (PME) algorithm was calculated through a 1.0 nm radius cut-off and grid spacing of 0.16 nm. The equilibrated system was subjected to MD simulation with two femtoseconds (fs) time steps, which continued until the system was stable. The output trajectory analysis includes Root Mean Square Deviation (RMSD) and Root Mean Square Fluctuation (RMSF) [57].

### Molecular docking

For performing docking simulations, the 3D structure of HLA-DR1 (DRB1*0101) Human class II histocompatibility protein (PDB id: 1AQD) [58] was retrieved from RCSB (www.rcsb.org) server. The PDB structure of 1AQD was refined by removing the present ligand from the crystal structure using UCSF Chimera v.1.14 software. The ClusPro 2.0 server (http://nrc.bu.edu/cluster/) was used for PspA_1-5c+p_-HLA-DRB1 docking [46]. Finally, the model of the ClusPro with the largest cluster size and the lowest binding free energy was checked for the interaction of the amino acids using the DimPlot tool in LigPlot + v.2.2.4 software and PYMOLv. 2.5 software [46]. Because the CDR region of the PspA molecule as a lactoferrin binding domain can attach to the N-lobe of the human lactoferrin to block surface accessibility of this bactericidal peptide [59], we performed a docking simulation between the PspA_1-5c+p_ construct and human lactoferrin N-lobe (PDB id: 2PMS) to furthermore validation of the 3D structure of the modeled PspA_1-5c+p_. The details of the interaction of the control PspA (Clade 2) with lactoferrin will help us to compare our designed construct with it in order to find out whether this designed PspA_1-5c+p_ is able to bind to lactoferrin like the original structure of PspA and maintain its 3D structure as in the natural state or not? The mentioned docking steps were repeated for molecular docking and further analysis of the PspA_1-5c+p_ construct and human lactoferrin N-lobe.

### In-silico immune response simulation

IL-4, IL-10, and IFN-γ inducing peptide construction of PspA_1-5c+p_ were predicted via the IL-4pred server (https://webs.iiitd.edu.in/raghava/il4pred/scan.php), IL-10Pred server (https://webs.iiitd.edu.in/raghava/il10pred/predict3.php), and IFNepitope server (https://webs.iiitd.edu.in/raghava/ifnepitope/predict.php), respectively. These servers can generate overlapping peptides of the query protein/antigen sequence and predict the cytokine-inducting ability of these antigenic regions. Furthermore, *in-silico* mammalian immune simulations against PspA_1-5c+p_ were designed utilizing the C-ImmSim server (http://150.146.2.1/C-IMMSIM/index.php). This immune response simulator server uses machine learning methods and a position-specific score matrix (PSSM) to predict immune interactions. According to the literature, three injections of the PspA_1-5c+p_ construct were administered at intervals of 4 weeks, on days 1, 30, and day 60. For this purpose, the simulation parameters were set as follows: vaccine injection not containing LPS, time steps at 1, 84, and 168; the entire simulation ran 1400 time steps (about 15 months), the random seed of 12,345, and the simulation volume of 10. Each step is 8 h. HLA alleles of parameters were also set based on predominant human HLA alleles (HLA-A*1101, HLA-B*3501, and HLA-DRB1*0101) [55, 60].

### Codon adaptation, in-silico cloning, and RNA structure

Using the Genscript (https://www.genscript.com/tools/rare-codon-analysis/) and NovoPro servers (https://www.novoprolabs.com/tools/codon-optimization/) [61], the codon adaptation of the PspA_1-5c+p_ sequence was carried out according to *E. coli K12* codon usage. We used the mFold server (http://www.unafold.org/mfold/applications/rna-folding-form.php/) [62] and Visual Gene Developer software [63] to analyze the mRNA secondary PspA_1-5c+p_ structure mainly by using thermodynamic methods (the Gibbs free energy). *In-silico* cloning of the designed PspA_1-5c+p_ sequence was carried out in pET-28a using the SnapGene 6.0 software. Finally, Biomatik Corporation (Cambridge, Ont., Canada) synthesized the optimized sequence of PspA_1-5c+p_ [53, 60].

### Expression and purification of recombinant PspA_1-5c+p_

The recombinant (PspA_1-5c+p_ -pET28a) vector was transformed into the chemically prepared competent *E. coli BL21* (DE3) cell via heat shock transformation. Positive clones were recognized by restriction enzyme digestion and colony PCR with universal T7 primers (https://www.addgene.org/mol-bio-reference/sequencing-primers/). Expression of the recombinant PspA_1-5c+p_ was induced by adding 1 mM Isopropyl-β-D, Thiogalactopyranoside (IPTG) (Thermo Fisher Scientific, USA) in Luria–Bertani Broth (LB) medium (Sigma Aldrich, USA) at 37 °C and incubated for 16 h, then was evaluated by 12% SDS-PAGE, and using HRP-conjugated anti-His tag antibody (Sigma, USA) confirmed by western blot analysis. By the manufacturer's instructions (Qiagen, Hilden, Germany), the recombinant PspA_1-5c+p_ was purified using a Ni–NTA column (Qiagen, Hilden, Germany) under native conditions. The purified PspA_1-5c+p_ was dialyzed with dialysis tubing (cutoff 12KDa) overnight at 4 °C against PBS (Sigma, USA) and measured by the Bradford protein assay [47]. A Limulus amebocyte lysate (LAL) test was done to assess LPS contamination using the LAL kit (Lonza QCL-1000 ®, Basel, Switzerland).

### Animal and immunization

Six to eight-week-old male BALB/c mice were purchased from the Pasteur Institute of Iran (Karaj, Iran) and immunized intraperitoneally three times at 14-day intervals with 10 μg of recombinant PspA_1-5c+p_ construct in PBS solution plus the Alum adjuvant (Imject TM Alum, Thermo Fisher Scientific, USA) at 1:1 (v/v) in a final volume 200 μl per mouse. The control group was injected with PBS and Alum. Before injections and two weeks after the last injection, sera from blood samples in each group were collected and stored at − 20 °C [47, 64].

#### Ethics

All animal experiments were done in accordance with the Institutional Animal Care and Use Committee’s guidelines Animals (Scientific Procedures) Act of Pasteur Institute of Iran and Islamic Azad University-Science and Research Branch. Ethical approval was obtained from the Institutional Research Ethics Committee, Islamic Azad University-Science and Research Branch (approval ID: IR.IAU.SRB.REC.1398.065).

#### Assessment of immune response

The presence of specific IgG antibodies was analyzed in the experimental sera using indirect ELISA. Briefly, the 96-well ELISA plate (Nanc MaxiSorp, Thermo Fisher, USA) was coated with 100 μl of the recombinant PspA_1-5c+p_ (1 μg/well) in coating buffer (0.05 M carbonate bicarbonate buffer, pH 9.6) overnight at 4 °C, then blocked with 5% bovine serum albumin (BSA; Sigma, USA) in PBST (PBS containing 0.05% Tween20). Following, three times wash with PBST; 100 μl of 0.001 diluted sera in blocking buffer was added to the plate and incubated for one hour at 37 °C. Afterward, the 1:10,000 dilution of HRP-conjugated anti-mouse total IgG (Sigma, USA) was used and incubated for one hour at 37 °C. After washing, the plate was incubated with the tetramethylbenzidine (TMB) substrate (Thermo Fisher Scientific, USA) to evaluate antibody reactivity at 450 nm using an Epoch absorbance microplate reader (BioTek Company) [47, 65].

#### Cross-reactivity analysis of anti-PspA_1-5c+p_ using whole cell ELISA

The sera were analyzed for investigation of the cross-reactivity of the anti-PspA_1-5c+p_ IgG against three strains of the pneumococcus, representing two families of the PspA including strains ATCC 49619 (Clade 1), ATCC 6305 (Clade 2), and ATCC 700678 (Clade 5) using the whole-cell ELISA test according to the method described by Ahmadi et al. [7]. Briefly, the 96-well ELISA plate was coated with 100 μl of the whole cell of bacterial suspensions overnight at 4 °C, which were grown to log phase in BHI broth (10^7^ cells/well) and then blocked with 10% BSA in PBST. The practical steps continued as described above (indirect ELISA).

#### Serum bactericidal assay (SBA)

The SBA assay was performed to evaluate the complement-mediated killing features of the anti-PspA_1-5c+p_ antibody against three strains of the pneumococcus expressing two families of the PspA. For this purpose, Thermo Scientific Nunc™ 96-Well Polystyrene Round Bottom microwell plates were coated with 12.5 μl of the three strains of pneumococcus at 10^5^ CFU/ml (based on the standard of 0.5 McFarland) separately, and 12.5 μl of diluted inactivated serum sample at 56 °C for 30 min (1:2 to 1:64). Afterward, fresh infant rabbit serum (4%) was added to each well as a source of the complement. At two intervals (0 and 2 h), the sample from each well was cultured in blood agar media. After 18–24 h incubation at 37 °C in 5% CO_2_, the colony-forming unit of the bacteria was counted. The wells containing bacteria and rabbit complement were used as a negative control [64, 65].

#### Opsonophagocytic killing activity (OPK)

The serum of immunized BALB/C with the PspA_1-5c+p_ construct was evaluated for Opsonophagocytic killing (OPK) activity of the anti-PspA_1-5c+p_ antibody using phagocyte cells against three strains of the pneumococcus, strain ATCC 49619, ATCC 6305, and ATCC 700678. Pneumococcus strains were prepared at 10^7^ CFU/ml (based on the standard of 0.5 McFarland). For macrophage cell collection from the peritoneal cavity of the naïve mice, 10 ml of the RPMI and FBS 10% were inoculated intraperitoneally in anesthetized mice. Afterward, the aspirated contents of the peritoneum were washed with RPMI and 10% FBS, and finally, live phagocyte cells were measured by the Neubauer slide. For OPK assay, the 100 μl of inactivated sera were incubated with 100 μl of the pneumococcus strains, and then 100 μl of the phagocyte cells (1 × 10^6^ cells/ml) and infant rabbit serum (4%) were added. Subsequently, 25 μl of the sample was cultured on a blood agar plate at two intervals (0 and 90 min). After 18–24 h incubation at 37 °C in 5% CO_2_, the colony-forming unit of bacteria was counted. The assessment of the opsonic activity of the anti-PspA antibody against the three pneumococcus strains compared to the PBS group was measured using the following formula:

Percentage of killed bacteria = [1 − (CFU of immune serum/CFU of pre-immune serum)] × 100 [47, 65].

### Statistical analysis

Statistical analysis was conducted using GraphPad Prism 6 software. One and two-way analysis of the variances (ANOVA) followed by Tukey’s multiple comparison test was performed for the analysis of immune responses. All experiments were performed in triplicate and expressed as the average ± S.D. P-values of less than 0.05 were considered statistically significant.

## Results

### Sequence retrieval

Additional file 1: Table S1 showed the results of sequence retrieval of five PspA clade types from the NCBI server, the subcellular localization, transmembrane helices, and signal peptide of the proteins. The results of the subcellular localization showed PspA proteins in five clades are extracellular or cytoplasmic localization. The results of the online server TMHMM showed the PspA proteins have a maximum of one transmembrane helices. The transmembrane helices prediction can help us to predict the state of the cloning, expression, and purification of the recombinant protein. The higher antigenic proteins with zero or one transmembrane helices were selected for the development of a vaccine, and proteins with multiple transmembrane helices should be eliminated due to their difficult cloning, expression, or purification [32]. The online server signalP-5.0 also showed PspA proteins have one signal peptide at positions 31 and 32.

### Immunoinformatics analysis

#### Defining B‑cell epitopes

The schematic results of the IEDB server are shown in Additional file 1: Fig. S1. The immunodominant overlapped predicted linear B-cell epitopes with high antigenicity, surface accessibility, flexibility, and hydrophilicity in CDR regions of each PspA were selected using BCPred and IEDB servers and provided in Additional File1 Table S2. According to the B-cell epitope prediction servers, the cross-reactive regions of each clade had at least five B-cell epitope sequences of ~ 6–25 mer in length with VaxiJen scores of 0.5 to 1. Some of the predicted epitopes had antigenicity scores of 2 to 3.3. According to the results of the 3D structure predictions of the PspA clade using the I-TASSER server, Model 1 with the highest C-score was chosen for refinement with the GalaxyRefine server. After the refinement of the predicted structure of each clade, Ramachandran plot analysis and ProSA validation are shown in Additional File1 Figure S2. Analysis of the predicted models for each PspA clade with C-score values, TM-score, Galaxy-refine scores, Rama favored score, ERRAT score, MolProbity score, and ProSA Z-score are shown in Additional file 1: Table S3. The continuous predicted B-cell epitopes in each PspA clade are also shown in Additional file 1: Fig. S1 and Table S4.

#### Helper T-cell (HTL) epitopes

The results of predicted helper T-cell epitopes from PspA CDRs using IEDB (percentile rank < 20), RANKPEP server, and MHCPred server (IC50 ≤ 100 nM) are shown in Additional file 1: Tables S5–S7. Eight human HLA-DRB1 alleles (DRB1*01:01, DRB1*03:01, DRB1*04:01, DRB1*07:01, DRB1*08:01, DRB1*11:01, DRB1*13:01, and DRB1*15:01) and three mouse alleles (H2-IAb, H2-IAd, and H2-IEd) had been considered for predictions [44–47]. The results of the PIR peptide-matching program showed that the predicted epitope sequences had no similarity with the human proteome. Some predicted B-cell epitopes were also predicted as HTL epitopes.

### Subunit PspA_1-5c+p_ vaccine construction

**Fig. 2:**
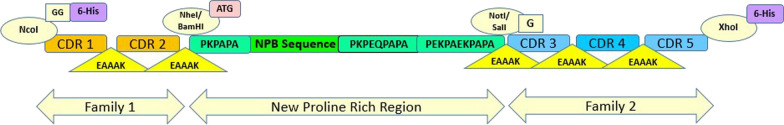
Schematic representation of the final PspA_1-5c+p_ vaccine candidate. To cover the maximum cross-reactivity between PspA two families and the diversity of all PRD groups, the 614 amino acid long peptide sequence contains immunodominant B-cell and T-cell epitopes as the truncated domain of the CDRs and the highly conserved region of NPB and repetitive motifs of PRD group. The CDR regions of the PspA family1 (orange) at the amino-terminal end are connected to the multi-epitope sequence of the PRD (green) via an EAAAK linker (yellow). PspA family 2 CDR regions (blue) are also linked using an EAAAK linker (yellow) together with PRD in the carboxy-terminal of the construct. Two 6 × His tags are added to the amino and carboxyl terminus of the construct for purification and identification purposes. For the cloning of the PspA_1-5c+p_ construct into the pET28a vector, the restriction enzyme sites (*Nco*I and *Xho*I) are considered

The chosen sequences from each PspA clade contain overlapped immunodominant regions of the surface and high antigenic epitopes of B and T-cells, as the truncated domain of PspA residues from each clade. Position 193 to 294 AA from Clade 1 and position 223 to 318 AA of Clade2 PspA were located in the N-terminal of the PspA_1-5c+p_ construct as a representative of the truncated domains of PspA Family1. Based on Mukerji’s study [21], we selected sequences representing each PRD group’s repetitive motif. The new region of the proline-rich domain with the highly conserved and immunogenic region of NPB and PR epitopes was located in the center of the PspA_1-5c+p_ construct. Finally, the truncated domain of PspA from Clades 3, 4, and 5 was located in the C-terminal of the PspA_1-5c+p_ construct, representing the PspA Family2. Position 346 to 444 AA of Clade 3, position 276 to 374 AA of Clade 4, and position 273 to 392 AA of Clade 5 were chosen. The above regions were fused with the EAAAK linker. In addition, a 6xHis tag was added to the N and C terminus for easy protein purification (Fig. 2). The final PspA_1-5c+p_ consisting of 614 amino acid residues was antigen and non-toxic. The amino acid sequence of the final PspA_1-5c+p_ construct has been shown in Table 1.

**Table 1:** The amino acid sequence of the final PspA_1-5c+p_ construct

Amino acid sequence	
MGHHHHHHLEKALKEIDESDSEDYVKEGLRAPLQFELDVKQAKLSKLEELSDKIDELDAEIAKLEKDVEDFKNSDGEQAGQYLAAAEEDLVAKKAELEKTEADLKKAVNEEAAAKLKEIDESESEDYAKEGFRAPLQSKLDAKKAKLSKLEELSDKIDELDAEIAKLEDQLKAAEENNNVEDYFKEGLEKTIAAKKAELEKTEADLKKAVNEAAAKGSASMPKPAPAQQAEEDYARRSEEEYNRLTQQQPKPEQPAPAPEKPAEKPAPAVDAAAEAAAKLEKLLDSLDPEGKTQDELDKEAEEAELDKKADELQNKVADLEKEISNLEILLGGADSEDDTAALQNKLATKKAELEKTQKELDAALNELGPDGDEEETEAAAKLEDAELELEKVLATLDPEGKTQDELDKEAAEAELNEKVEALQNQVAELEEELSKLEDNLKDAETNNVEDYIKEGLEEAIATKKAELEKTQKELDAALNEEAAAKLEDAELELEKVLATLDPEGKTQDELDKEAAEDANIEALQNKVADLENKVAELDKEVTRLQSDLKDAEENNVEDYVKEGLEKALTDKKVELNNTQKALDTAPKALDTALNELGPDGDEEETLE	

#### Physicochemical properties and solubility prediction

Using the ProtParam server, the molecular weight (MW) of the final PspA_1-5c+p_ construct was predicted to be 67.93 kDa. The theoretical isoelectric point value (pI) was 4.39. The instability index (II) was calculated to be 40.12. The aliphatic index and grand average of the hydropathicity (GRAVY) were estimated to be 82.23 and -0.997, respectively. The half-life was assessed to be 30 h in mammalian reticulocytes in vitro, > 20 h in yeast, and > 10 h in *E. coli in-vivo* [51]. Using the Solpro server, the PspA_1-5c+p_ construct was predicted to be soluble upon overexpression in *E. coli* with a solubility probability score of 0.905.

#### Prediction of the secondary tertiary structures of PspA_1-5c+p_

**Fig. 3:**
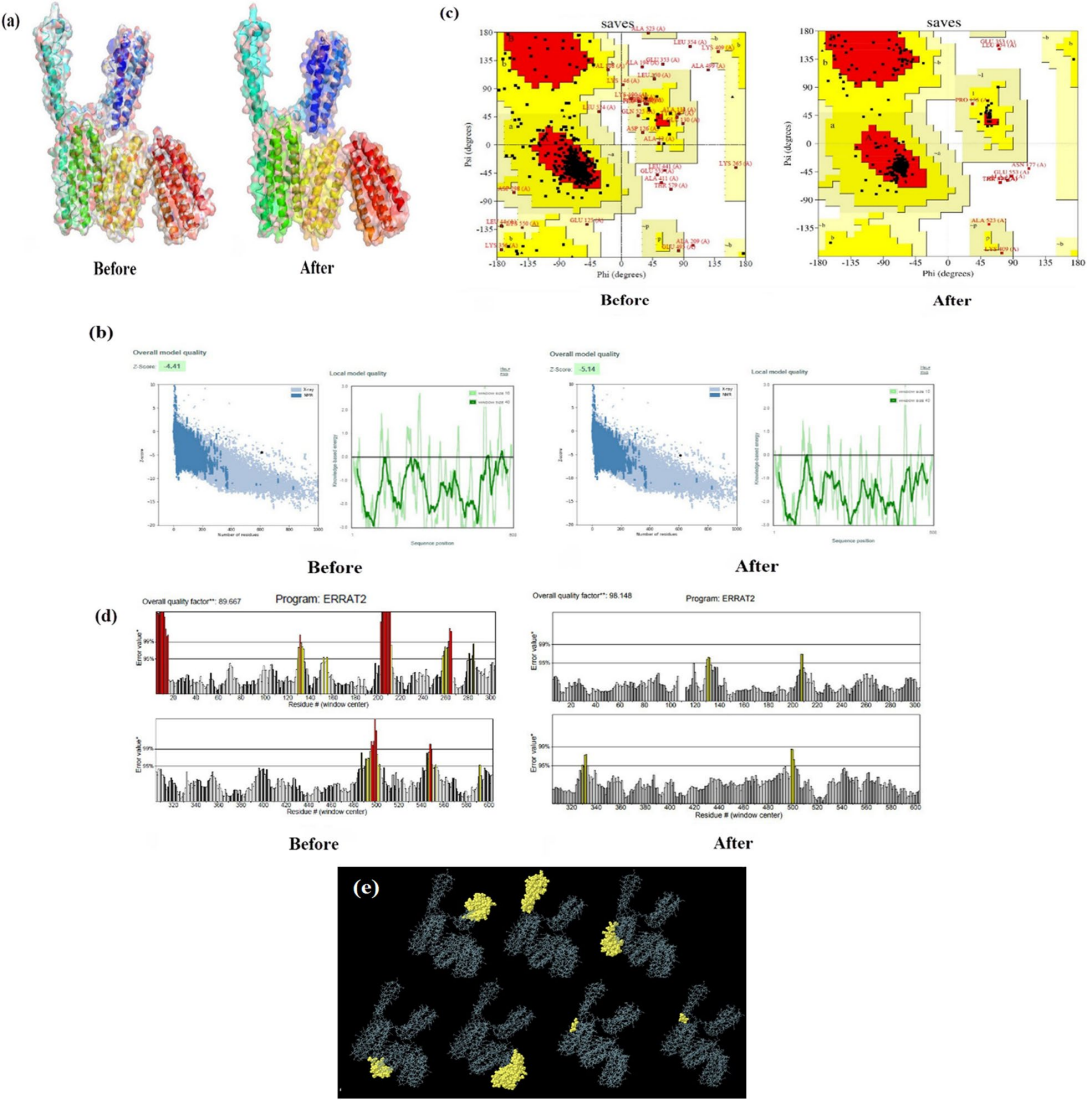
PspA_1-5c+p_ modeling, refinement, validation, and prediction of the conformational B-cell epitopes. **a** The I-Tasser 3D homology modeling of the PspA_1-5c+p_ construct before and after refinement was displayed by PyMol v.2.5 software. Each domain is indicated in color. Validation of the model before and after refinement using **b** ProSA Web, **c** Ramachandran plot, and **d** ERRAT plot. The ProSA Web analysis shows the z-scores of − 4.41 and − 5.14 before and after refinement, respectively, and the plot of the residue scores showing local model quality by plotting energies as a function of amino acid sequence position is also shown. Ramachandran plot analysis after refinement showed 92.4%, 6.4%, and 1.2% of PspA_1-5c+p_ protein residues were in preferred, valid, and non-valid (outlier) regions, respectively. In general, positive values correspond to problematic or erroneous parts of the input structure. The overall quality score of the selected model before and after refinement is 89.66% and 98.14%, respectively, using the ERRAT2 server. These values are expressed as the percentage of the protein for which the estimated error value falls below the 95% rejection limit. Two lines in the error axis reveal the confidence with which it is possible to eliminate areas that exceed this error value. Good high-resolution structures generally produce values around 95% or higher. **e** The conformational B-cell epitopes using the Elipro server on a refined and validated final 3D PspA_1-5c+p_ model were predicted to be located in seven conformational B-cell epitopes. The conformational B-cell epitopes are shown in yellow and the gray parts are the rest of the residues

The GOR V prediction server reported that the final PspA_1-5c+p_ secondary structure contains 83.22% alpha-helix, 0.49% extended strand, and 16.28% random coil. The secondary structure of the final PspA_1-5c+p_ using PSIPRED prediction is shown in Additional file 1: Fig. S3. RaptorX Property server reported 199 residue positions (32%) as disordered. The I-TASSER server predicted five models of tertiary structure for the PspA_1-5c+p_ construct based on 10 threading templates. The five predicted models for the PspA_1-5c+p_ construct had C-score values between − 3.65 and − 0.58. Model 1, with the highest C-score of − 0.58, was chosen for further refinement (Fig. 3a). This model had an estimated TM-score and RMSD of 0.64 ± 0.13 and 9.1 ± 4.6 Å, respectively.

#### Tertiary structure refinement and validation

We used two servers for refinement. The optimal final model from Galaxy Refine was found to be based on various parameters including Ramachandran plot score (94.6%), RMSD (0.244), MolProbity (1.892), GDT-HA score (0.9926), clash score (10), and poor rotamers score (0.6). This model was selected as the final PspA_1-5c+p_ vaccine candidate model for further investigation. The quality and potential errors in the 3D model were verified by ProSA-web and ERRAT. The PspA_1-5c+p_ protein fell close to the range of scores commonly found in native proteins of comparable size with the ProSA-web Z-score of − 5.14 (Fig. 3b). The Ramachandran plot analysis of the Saves server revealed that, among the 608 residues, 526 (92.4%) and 36 (6.4%) in the protein were in the most favored and allowed regions, respectively. There were only seven residues (1.2%) in the disallowed region, indicating that the predicted model is acceptable (Fig. 3c). The overall quality factor of the chosen model after the last refinement was 98.14% (Fig. 3d). Then, using the Elipro server, the prediction of conformational B-cell epitopes was performed on a refined and validated 3D final PspA_1-5c+p_ model (Fig. 3e). The results revealed that a total of 608 residues were situated in seven conformational B-cell epitopes, with scores ranging from 0.514 to 0.818. The conformation epitopes ranged in size from three to 91 residues. The details of conformational B-cell epitope prediction are presented in Table 2. The PspA_1-5c+p_ final construct was also analyzed to be predicted as non-toxic and immunogenic, with an antigenicity score of 0.77.

**Table 2:** The conformational B-cell epitope prediction for the final PspA_1-5c+p_ construct using EliPro server

No	Residues	Number of residues	Score
1	A:K115, A:L116, A:K117, A:E118, A:I119, A:D120, A:E121, A:S122, A:E123, A:S124, A:E125, A:D126, A:Y127, A:A128, A:K129, A:E130, A:G131, A:F132, A:R133, A:A134, A:P135, A:L136, A:Q137, A:S138, A:K139, A:L140, A:D141, A:A142, A:K143, A:K144, A:A145, A:K146, A:L147, A:S148, A:K149, A:L150, A:E151, A:E152, A:L153, A:S154, A:D155, A:K156, A:I157, A:D158, A:E159, A:L160, A:D161, A:A162, A:E163, A:I164, A:A165, A:K166, A:L167, A:E168, A:D169, A:Q170, A:L171, A:K172	58	0.816
2	A:M1, A:G2, A:H3, A:H4, A:H5, A:H6, A:H7, A:H8, A:L9, A:E10, A:K11, A:A12, A:L13, A:K14, A:E15, A:I16, A:D17, A:E18, A:S19, A:D20, A:S21, A:E22, A:D23, A:Y24, A:V25, A:K26, A:A59, A:E60, A:I61, A:A62, A:K63, A:L64, A:E65, A:K66, A:D67, A:V68, A:E69, A:D70, A:F71, A:K72, A:N73, A:S74, A:D75, A:G76, A:E77, A:Q78, A:A79, A:G80, A:Q81, A:Y82, A:L83, A:A84, A:A85, A:A86, A:E87, A:E88, A:D89, A:L90, A:V91, A:A92, A:K93, A:A95, A:E96, A:K99	64	0.78
3	A:D489, A:A490, A:E491, A:L492, A:E493, A:L494, A:E495, A:K496, A:V497, A:L498, A:A499, A:T500, A:L501, A:D502, A:P503, A:E504, A:G505, A:K506, A:T507, A:Q508, A:D509, A:E510, A:L511, A:D512, A:K513, A:E514, A:A515, A:A516, A:D518, A:A519, A:N520, A:I521, A:E522, A:A523, A:N526, A:K527, A:D530, A:N533, A:K534, A:V535, A:A536, A:E537, A:L538, A:D539, A:K540, A:E541, A:V542, A:T543, A:R544, A:L545, A:Q546, A:S547, A:D548, A:L549, A:K550, A:D551, A:A552, A:E553, A:E554, A:N555, A:N556, A:V557, A:E558, A:D559, A:Y560, A:V561, A:K562, A:E563, A:G564, A:L565, A:P587, A:K588, A:A589, A:L590, A:D591, A:T592, A:A593, A:L594, A:N595, A:E596, A:L597, A:G598, A:P599, A:D600, A:G601, A:D602, A:E603, A:E604, A:E605, A:T606, A:L607	91	0.738
4	A:Y183, A:E186, A:G187, A:E189, A:K190, A:T191, A:I192, A:A193, A:A194, A:K195, A:K196, A:A197, A:E198, A:L199, A:E200, A:K201, A:T202, A:E203, A:A204, A:D205, A:L206, A:K207, A:K208, A:A209, A:V210, A:N211, A:E212, A:Q254, A:P255, A:A256, A:P257, A:A258, A:P259, A:E260, A:K261, A:P262, A:A263, A:E264, A:K265, A:P266, A:A267, A:P268, A:A269, A:V270, A:D271, A:A273	46	0.709
5	A:L331, A:G332, A:G333, A:D373, A:E374, A:E376, A:T377, A:E378, A:A379, A:A380, A:A381, A:E432, A:E433, A:S435, A:K436, A:L437, A:E438, A:D439, A:N440, A:L441, A:K442, A:D443, A:A444, A:E445, A:T446, A:N447, A:N448, A:V449, A:E450, A:D451	30	0.647
6	A:N179, A:D182, A:K185	3	0.548
7	A:A173, A:A174, A:E175, A:E176	4	0.514

### Molecular dynamic simulation

**Fig. 4:**
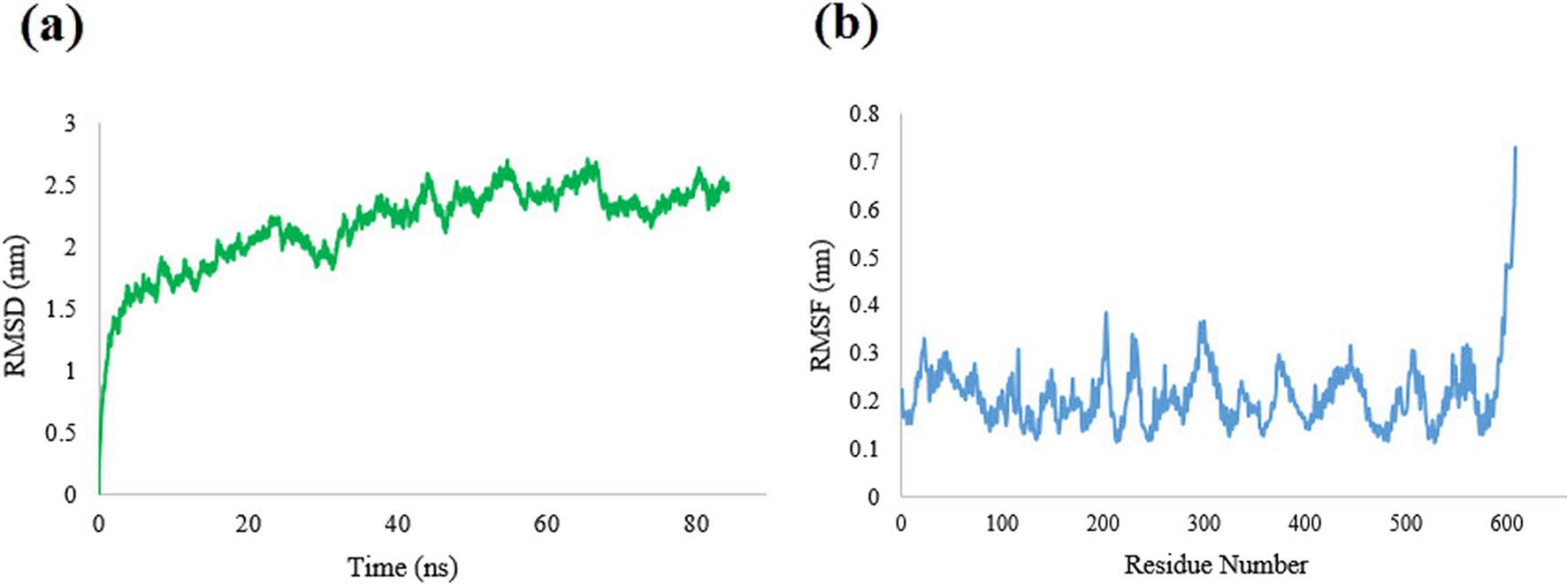
Molecular Dynamics simulation of the PspA_1-5c+p_ protein. **a** The RMSD plot of the PspA_1-5c+p_ showed the steady state of the model at 50 ns. **b** The RMSF plot of the PspA_1-5c+p_ protein revealed the low flexibility for residues, except for the C-terminal region

To assess the stability and dynamics of the designed PspA_1-5c+p_ vaccine candidate, molecular dynamic simulation was performed until the protein structure reached the stability state (Fig. 4). To find PspA_1-5c+p_ conformational changes or stability against the initial structure, the root mean square deviation (RMSD) based on the structure of the backbone was applied. The RMSD plot analysis revealed that the protein structure deviated until 50 ns and then reached a plateau with a maximum RMSD value of 2.45 nm. To evaluate the fluctuated residues of PspA_1-5c+p_ protein, root mean square fluctuation (RMSF) was measured. The plot of RMSF showed the RMSF values less than 0.35 nm for almost residues, indicating low changes in the structure of PspA_1-5c+p_ protein. But the C-terminal residues of PspA_1-5c+p_ showed more flexibility with a RMSF value of 0.7 nm (Fig. 4b).

### Molecular docking results

**Fig. 5:**
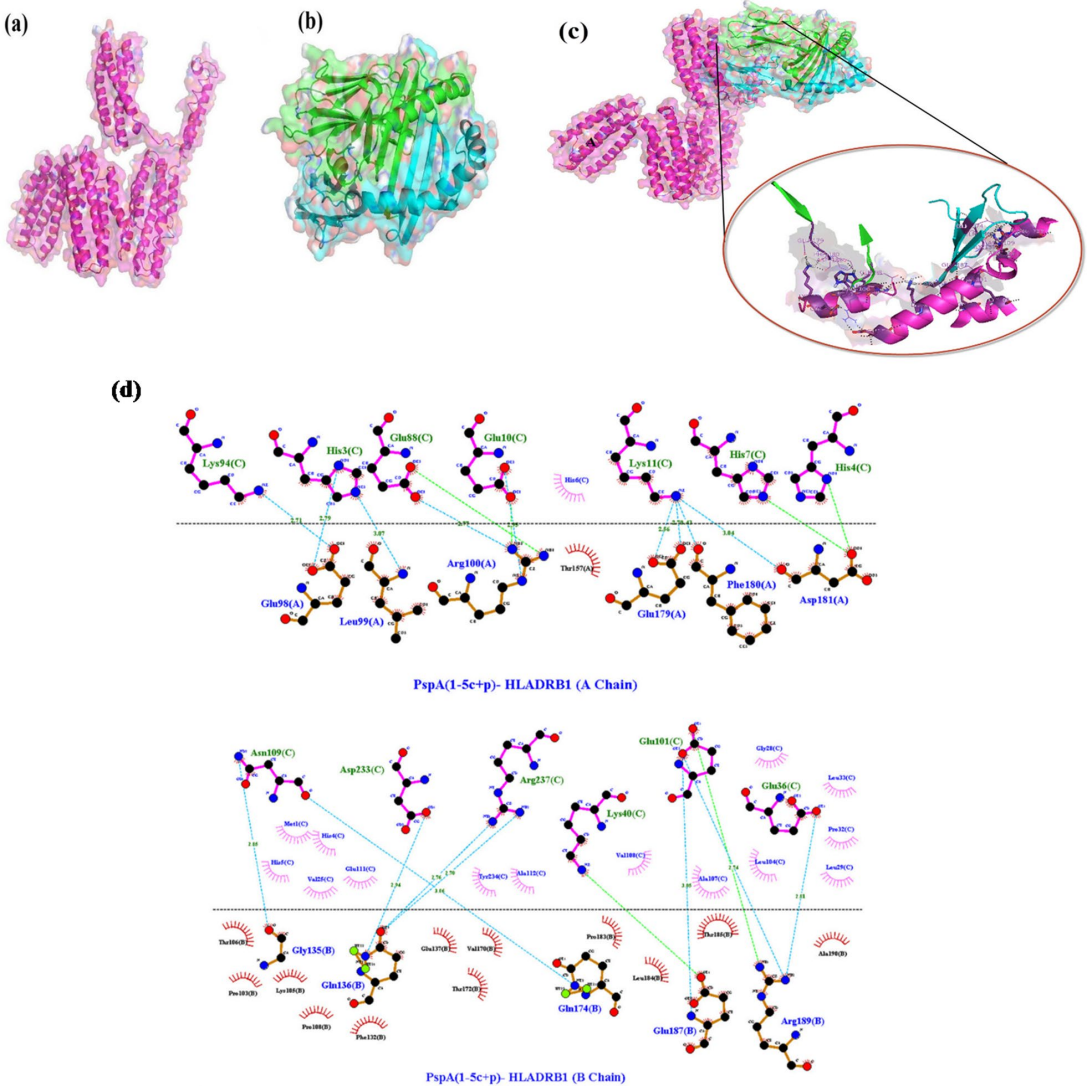
Molecular docking of the PspA_1-5c+p_ and HLADRB1*01:01 (Chains A and B). **a** The 3D structure of the PspA_1-5c+p_ before docking. **b** The 3D structure of the HLADRB1*01:01 (Chains A and B) (PDB ID: 1AQD) before docking. **c** Docked complex of the PspA_1-5c+p_ with HLADRB1*01:01. The cartoon representation of the PspA_1-5c+p_-HLADRB1*01:01 complex is illustrated using the PyMOL software. The PspA_1-5c+p_, chains A and B of the HLADRB1*01:01 are shown in magenta, green, and cyan, respectively. The lowest energy value of this complex model was -744.3 kcal.mol^−1^, indicating good binding affinity. **d** Dimplot interaction diagram between PspA_1-5c+p_ residues and the HLADRB1*01:01 molecule in the docked complex. A number of 7 and 6 residues of PspA_1-5c+p_ were paired with 6 residues from chain A and 5 residues from chain B of the HLA-DRB1_01:01 molecule, respectively. PspA_1-5c+p_ residues, HLADRB1*01:01 residues, hydrogen bonds, salt bridges, and unbound residues are exhibited in green, blue, blue dashed lines, green dashed lines, and red/pink eyelashes, respectively

**Fig. 6:**
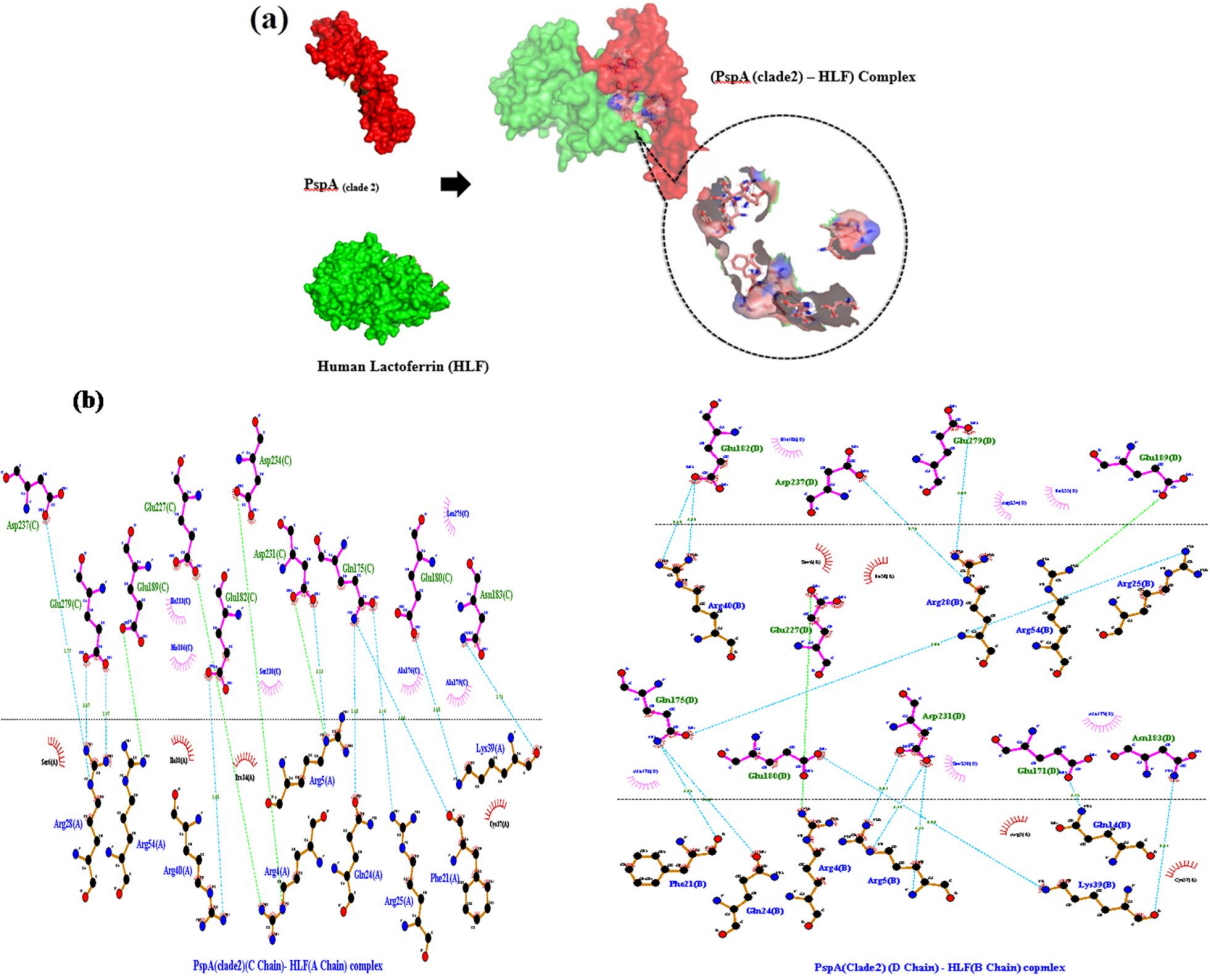
**a** Molecular docking of the PspA (clade2) and HLF as control of docking. Two 3D structures of the docked complex of the PspA with HLF have been shown. The surface depictions of the PspA-HLF complex are illustrated using the PyMOL software. The PspA and HLF have been shown in red and green, respectively. **b** Dimplot interaction plot between PspA (clade 2) residues and HLF molecule in the docked complex as a PDB:2PMS control complex. PspA (Clade2) residues, HLA residues, hydrogen bonds, salt bridge, and non-bonded residues are exhibited in green, blue, blue dashed lines, green dashed lines, and red/pink eyelashes, respectively

**Fig. 7:**
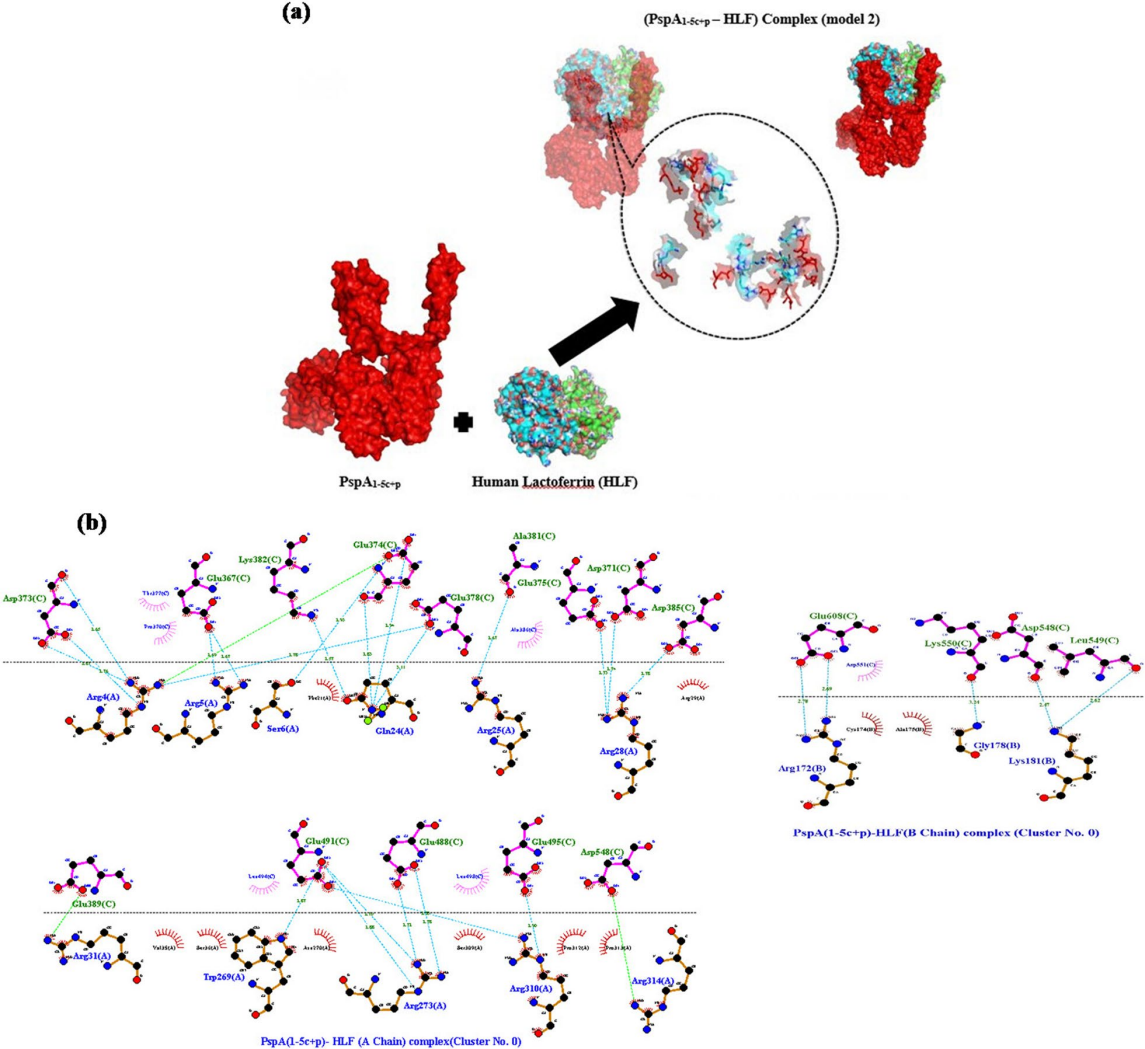
**a** Molecular docking of the PspA_1-5c+p_ (Family1 domain) with HLF (Chain A and B). The surface depictions of the PspA_1-5c+p_-HLF complex are illustrated using the PyMOL software. The PspA_1-5c+p_, chains A and B of the HLF, have been shown in red, green, and cyan, respectively**.** **b** Dimplot interaction plot between PspA_1-5c+p_ residues (Family1 domain) with HLF molecule in the docked complex (Cluster No. 0.0). PspA_1-5c+p_ residues, HLA residues, hydrogen bonds, salt bridge, and non-bonded residues are exhibited in green, blue, blue dashed lines, green dashed lines, and red/pink eyelashes, respectively

**Fig. 8:**
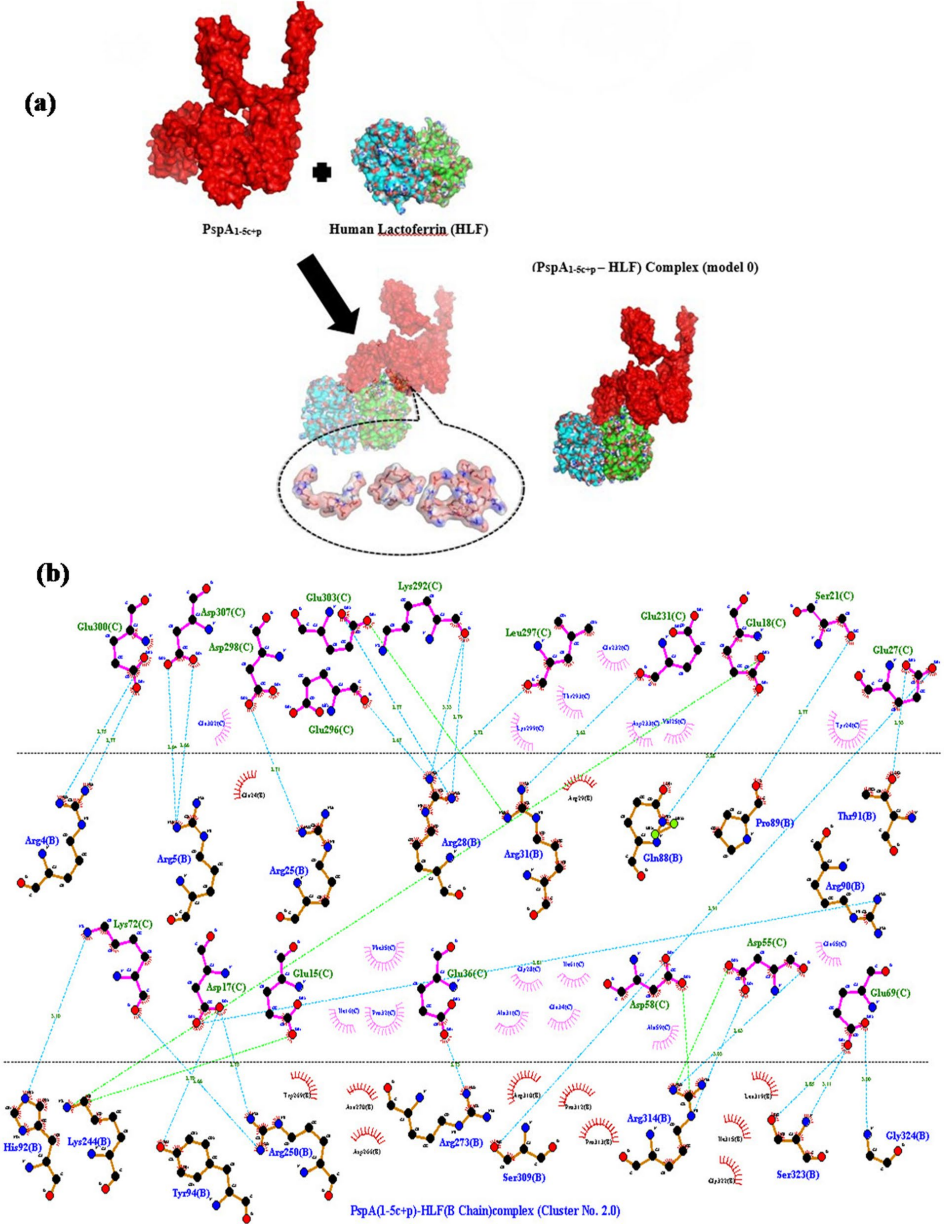
**a** Molecular docking of the PspA_1-5c+p_ (Family2 domain) with HLF (Chains A and B). The surface depictions of the PspA_1-5c+p_-HLF complex are illustrated using the PyMOL software. The PspA_1-5c+p_, chains A and B of the HLF, have been shown in red, green, and cyan, respectively**.** **b** Dimplot interaction plot between PspA_1-5c+p_ residues (Family2 domain) with HLF molecule in docked complex (Cluster No. 2.0). PspA_1-5c+p_ residues, HLA residues, hydrogen bonds, salt bridge, and non-bonded residues are exhibited in green, blue, blue dashed lines, green dashed lines, and red/pink eyelashes, respectively

ClusPro online server performed molecular protein–protein docking between refined PspA_1-5c+p_ and HLADRB1*01:01 (the most common binding allele in the Iran population). Cluster No. 0.00 of PspA_1-5c+p_-HLADRB1 docked complex with 64 members having the lowest energy of -744.3 kcal.mol^−1^ were selected for further analysis. The interaction surface residues of the docked complex were checked with Dimpolt tools in LigPlot^+^ software and visualized using PyMol software (Fig. 5). A total of 7 and 6 PspA_1-5c+p_ residues coupled with 6 and 5 residues of A and B chains from HLADRB1*01:01 molecule, respectively. Altogether, a number of 16 hydrogen bonds and 6 salt bridges, and many hydrophobic bonds, were formed between the PspA_1-5c+p_ residues and HLADRB1*01:01 molecule (Fig. 5 and Table 3). We docked the PspA_1-5c+p_ construct with Human Lactoferrin N-lobe (HLF) to furthermore 3D structure validation of modeled PspA_1-5c+p_ and analysis of 3D structure conformation preserving of each CDR region in the vaccine formulation. The structure of the PspA (clade2)-HLF docked complex with PDB id: 2PMS was used as a control. The output result of the ClusPro server exhibit 30 clusters for the docked complex ranked (0–29) according to cluster members with weighted scores of the cluster energies. Cluster No. 0.00 and 2.00 of the PspA_1-5c+p_-HLF docked complexes were chosen, which had different CDR binding positions to HLF, and maximum cluster members of 80 and 58 with the lowest energy of -987.2 and -1128.9 kcal.mol^−1^, respectively. The interaction surface residues of the PspA_1-5c+p_-HLF docked complex and PspA (clade2)-HLF control docked complex were analyzed with Dimpolt tools in LigPlot + software and visualized using PyMol software (Fig. 6 and Table 4). The results of comparisons between the PspA_1-5c+p_-HLF docked complex with PspA (clade2)-HLF control docked complex showed that PspA_1-5c+p_ construct could be connected to HLF molecule via both regions representing PspA families 1 and 2 in PspA_1-5c+p_ construct same as in a control docked complex. In coordination with the docked control molecule, in two models of the PspA_1-5c+p_-HLF docked complex, the most residues of the HLF that have been in contact with CDR residues include Arg4, Arg5, Arg25, Arg28, Arg31, Arg40, Gln14, Gln24, and lys39. The details of the number of hydrogen bonds and salt bridges, the name and number of residues involved in the interactions, and altogether the details of interaction-docked complexes are shown in Figs. 7 and 8, and Table 4. These results can be shown the good 3D structure conformation preserving of the CDR region in the PspA_1-5c+p_ vaccine formulation.

**Table 3:** Analysis of Dimplot 2D-interaction plot between PspA_1-5c+p_ residues and HLADRB1*01:01 molecule in the docked complex

PspA_1-5c+p_-HLADRB1*01:01 docked complex							
Atom name	Res. No.	Res. name	H-Bond Distance (A°)	Atom name	Res. No.	Res. name	H-bond distance (A°)
PspA_1-5c+p_				HLADRB1*01:01 (A chain)			
OE2	10	Glu	2.95	NE	100	Arg	2.95
OE1	88	Glu	2.77	NH2	100	Arg	2.77
ND1	3	His	2.79	OE2	98	Glu	2.79
NZ	94	Lys	2.71	OE1	98	Glu	2.71
NZ	11	Lys	2.7	OE1	179	Glu	2.7
NZ	11	Lys	2.56	OE2	179	Glu	2.56
NZ	11	Lys	2.43	O	180	Phe	2.43
NZ	11	Lys	3.04	O	181	Asp	3.04
PspA_1-5c+p_				HLADRB1*01:01 (B chain)			
O	109	Asn	3.06	N	174	Gln	3.06
ND2	109	Asn	2.85	O	135	Gly	2.85
OE2	101	Glu	2.74	NH2	189	Arg	2.74
OE1	36	Glu	2.81	Nh2	189	Arg	2.81
OE2	101	Glu	3.05	N	187	Glu	3.05
OD1	233	Asp	2.94	NE2	136	Gln	2.94
NH2	237	Arg	2.7	OE1	136	Gln	2.7
NH1	237	Arg	2.76	OE1	136	Gln	2.76

**Table 4:** Dimplot interaction plot analysis between PspA_1-5c+p_ residues and HLF molecule in the docked complex and control. **a** The interaction detail between PspA (clade2) residues and HLF A and B chains (as a docked control model). **b** The interatin detail between model 0 of PspA_1-5c+p_ residues (Family 2 Domain) and HLF A and B chains. And **c** The interaction detail between model 2 of PspA_1-5c+p_ residues (Family 1Domain) and HLF A and B chains

(a)							
PspA(clade2)-HLF docked complex (2PMS)							
Atom name	Res. No.	Res. name	H-bond distance (A°)	Atom name	Res. No.	Res. name	H-bond distance (A°)
PspA (clade2) (C chain)				HLF (A chain)			
ND2	183	Asn	2.76	O	39	Lys	2.76
OE1	180	Glu	3.05	NZ	39	Lys	3.05
NE2	175	Gln	3.04	O	21	Phe	3.04
OE1	175	Gln	3.14	NH1	25	Arg	3.14
NH2	175	Gln	2.68	OE1	24	Gln	2.68
OD1	231	Asp	3.23	NE	5	Arg	3.23
OE2	182	Glu	2.45	NH1	40	Arg	2.45
OE1	279	Glu	2.97	NH2	28	Arg	2.97
OE2	279	Glu	3.07	NH1	28	Arg	3.07
OD2	237	Asp	2.77	NE	28	Arg	2.77
PspA (clade2) (C chain)				HLF (A chain)			
ND2	183	Asn	2.81	O	39	Lys	2.81
OE1	180	Glu	2.92	N2	39	Lys	2.92
OE2	171	Glu	3.15	NE2	14	Gln	3.15
OD1	231	Asp	3.15	N	5	Arg	3.15
OD1	231	Asp	3.14	NE	5	Arg	3.14
OD2	231	Asp	2.91	NB2	5	Arg	2.91
NE2	175	Gln	2.53	OE1	24	Gln	2.53
NE2	175	Gln	3.25	O	21	Phe	3.25
OE2	279	Glu	3.02	NH1	28	Arg	3.02
OE1	175	Gln	3.06	NB1	25	Arg	3.06
OD2	237	Asp	2.76	NE	28	Arg	2.76
OE2	182	Glu	3.28	NB2	40	Arg	3.28
OE2	182	Glu	2.52	NB1	40	Arg	2.52

(b)							
PspA_1-5c+p_-HLF docked complex (Model 0)							
Atom name	Res. No.	Res. name	H-bond distance (A°)	Atom name	Res. No.	Res. name	H-bond distance (A°)
PspA_1-5c+p_ (C chain)				HLF (A chain)			
OD2	385	Asp	2.78	NB1	28	Arg	2.78
OD2	371	Asp	2.74	NB2	28	Arg	2.74
OE1	375	Glu	2.73	NB2	28	Arg	2.73
O	381	Ala	2.67	NB1	25	Arg	2.67
OE2	378	Glu	3.11	N	24	Gln	3.11
OE2	378	Glu	2.78	NB1	4	Arg	2.78
OE1	374	Glu	2.94	N	24	Gln	2.94
OE2	374	Glu	2.93	OC	6	Ser	2.93
O	374	Glu	2.83	N	24	Gln	2.83
N2	382	Lys	2.57	OE1	24	Gln	2.57
OE1	367	Glu	2.67	NB1	5	Arg	2.67
OE1	367	Glu	2.69	NB2	5	Arg	2.69
O	373	Asp	2.65	NB2	4	Arg	2.65
OD1	373	Asp	2.76	NE	4	Arg	2.76
OD2	373	Asp	2.67	NB2	4	Arg	2.67
OE1	495	Glu	2.9	NE	310	Arg	2.9
OE1	491	Glu	2.83	NB1	310	Arg	2.83
OE2	488	Glu	2.78	NB1	273	Arg	2.78
OE1	488	Glu	2.72	NB2	273	Arg	2.72
OE2	491	Glu	2.7	NE	273	Arg	2.7
OE2	491	Glu	2.88	NE1	273	Arg	2.88
OE2	491	Glu	2.87	NB1	269	Trp	2.87
PspA_1-5c+p_ (C chain)				HLF (B chain)			
O	549	Leu	2.62	NZ	181	Lys	2.62
O	548	Asp	2.47	NZ	181	Lys	2.47
O	550	Lys	3.24	N	178	Gly	3.24
OE2	608	Glu	2.69	NB1	172	Arg	2.69
OE1	608	Glu	2.78	NB2	172	Arg	2.78

(c)							
PspA_1-5c+p_ -HLF docked complex (Model 2)							
Atom name	Res. no	Res. name	H-bond distance (A°)	Atom name	Res. No.	Res. name	H-bond distance (A°)
PspA_1-5c+p_ (C chain)				HLF (A chain)			
–	–	–	–	–	–	–	–
PspA_1-5c+p_ (C chain)				HLF (B chain)			
OE2	27	Glu	2.93	OC1	91	Thr	2.93
OE1	27	Glu	2.91	OD1	309	Ser	2.91
OC	21	Ser	2.77	O	89	Pro	2.77
OE1	18	Glu	3.28	NE2	88	Gln	3.28
O	231	Glu	2.62	NB1	31	Arg	2.62
O	297	Leu	2.72	NB1	28	Arg	2.72
O	292	Lys	2.79	NB2	28	Arg	2.79
O	292	Lys	3.33	NB1	28	Arg	3.3
OE2	303	Glu	2.77	NB2	28	Arg	2.77
O	296	Glu	2.67	NB1	28	Arg	2.67
OD2	298	Asp	2.71	NB2	25	Arg	2.71
OD1	307	Asp	2.66	NB2	5	Arg	2.66
OD2	307	Asp	2.64	NB2	5	Arg	2.64
OE2	300	Glu	2.75	NB2	4	Arg	2.75
OE1	300	Glu	2.77	NB1	4	Arg	2.77
OD2	17	Asp	2.81	NB2	90	Arg	2.81
O	55	Asp	2.63	NB2	314	Arg	2.63
OD1	55	Asp	3.03	NE	314	Arg	3.03
OE1	36	Glu	2.73	NB2	273	Arg	2.73
OD1	17	Asp	2.75	NB1	250	Arg	2.75
O	72	Lys	2.66	NB2	250	Arg	2.66

### Codon adaptation, in-silico cloning, and RNA structure

**Fig. 9:**
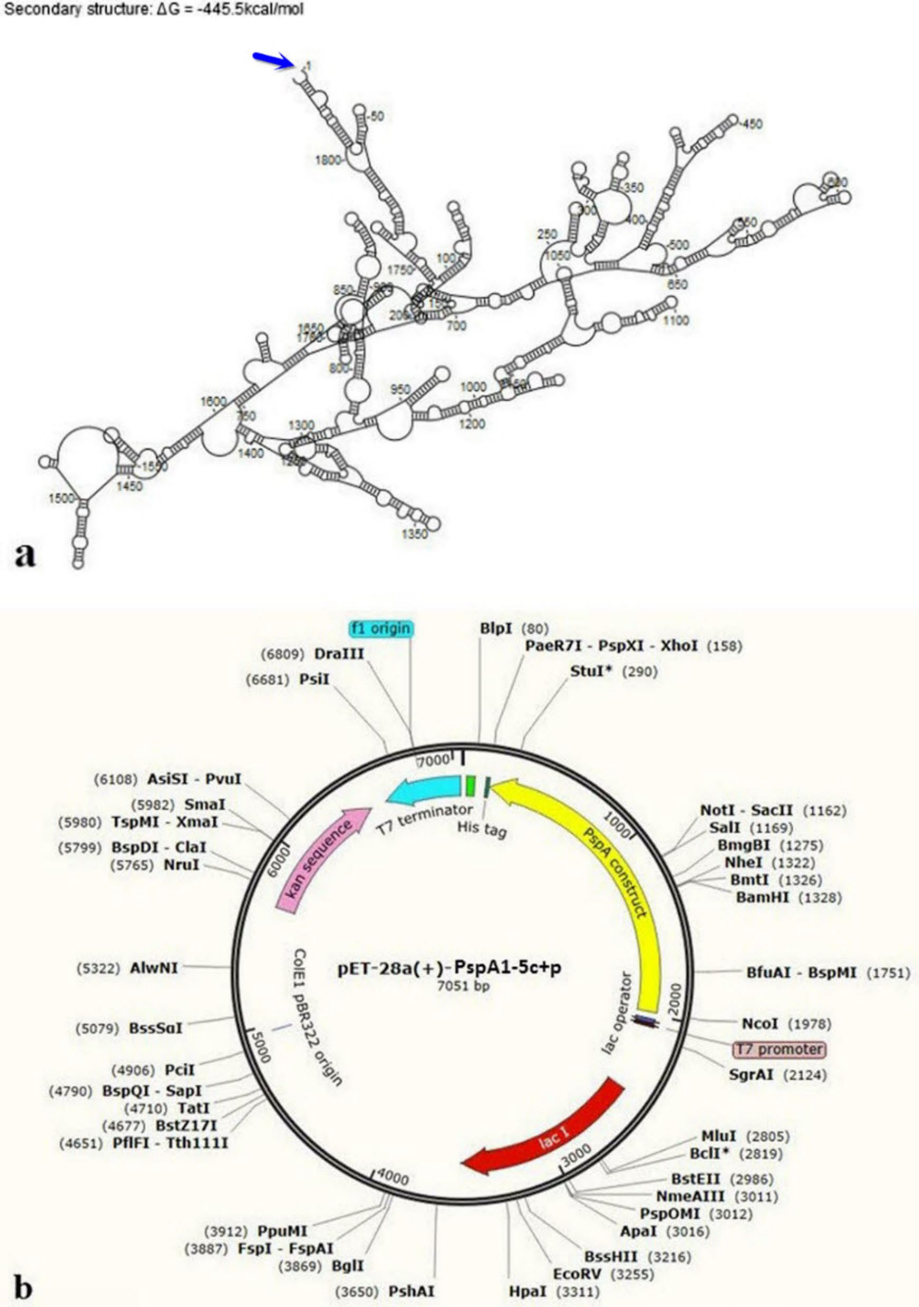
The mRNA folding analysis and PspA_1-5c+p_ construct *in-silico* cloning into the pET28a( +) expression vector. **a** The mRNA secondary structure of the PspA_1-5c+p_ construct with Gibbs free energy of -445.5 kcal.mol^−1^. **b** *In-silico* cloning of the final PspA_1-5c+p_ construct sequence into the pET28a ( +) expression vector. The yellow part represents the gene encoding PspA_1-5c+p_, and the black circle represents the vector backbone

Codon Adaptation Index (CAI) and GC content of the optimized codon sequence of PspA_1-5c+p_ with a length of 1826 bp in *E. coli* (strain K12) were 0.84 and 42.97%, respectively. These results showed good efficiency of the final vaccine candidate transcription and translation in the *E. coli* host. After the codon optimization, we analyzed the PspA_1-5c+p_ mRNA secondary structure (Fig. 9a). There was no observed unsuitable pseudoknot or loop at 5’ for transcription. The Gibbs free energy after sequence optimization for PspA_1-5c+p_ construct mRNA was − 445.5 kcal.mol^−1^ showing the lowest free energy and stable structure. Finally, using SnapGene 6.0 software, we inserted the optimized codon sequence into the pET28a ( +) vector between *Nco*I (1978) and *Xho*I (158), forming a clone with a total length of 7051 bp (Fig. 9b).

### In-silico immune response simulation

**Fig. 10:**
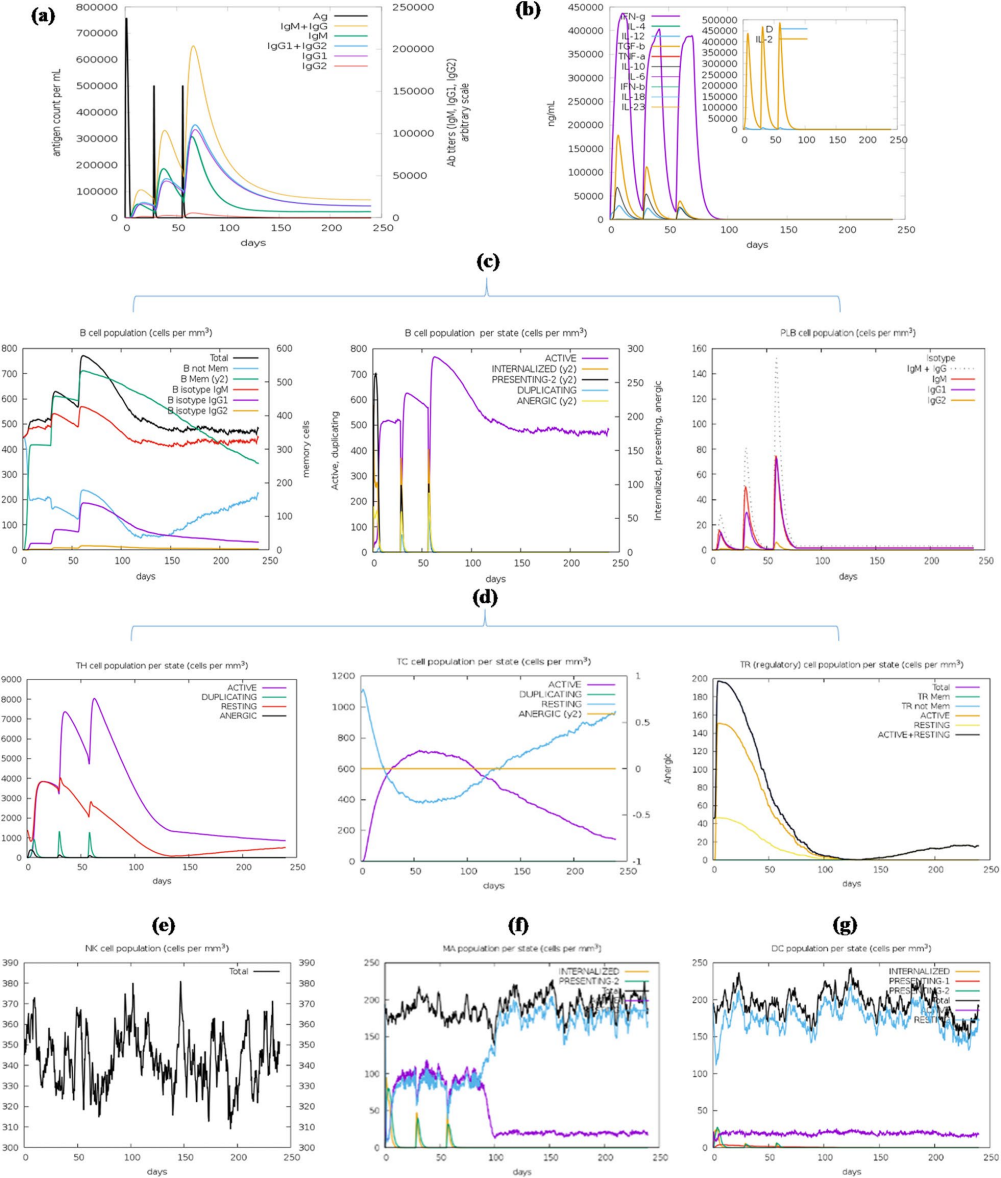
In-silico simulation of the immune response with PspA_1-5c+p_ vaccine. **A** Diversity of the immunoglobulin production in response to PspA_1-5c+p_ injections (PspA_1-5c+p_ antigen, and IgM, IgG1, and IgG2 subclasses are shown as black and colored peaks, respectively). **B** Cytokines and interleukin levels. The inset graph shows the level of IL-2 with the Simpson index, D shown by the dotted line, as a measure of diversity and danger signal along with the leukocyte growth factor IL-2. **C** The evolution of B-cell and plasma cell populations after the three administrations. **D** The evolution of T-helper, T-cytotoxic cell, and T regulatory cell populations after the injections. The cells not presented with the antigen were defined as being in the resting state, while the anergic state indicates the tolerance of the T-cells to the PspA due to repeated exposure. The evolution of the Natural killer cell population (**E**), Macrophage population (**F**), and Dendritic cell population (**G**)

Using the IL-4pred server, overlapping peptides of the query PspA_1-5c+p_ sequence were generated, and antigenic regions of PspA_1-5c+p_ that have a potential for inducing IL-4 were predicted and shown in Additional file 1: Table S8. The results of the IL-10pred web server showed that the PspA_1-5c+p_ construct with a score of 0.99, was predicted as an IL-10 inducer. Using the IFNepitope server, the PspA_1-5c+p_ construct was scanned and predicted to have many IFN-γ inducing MHC class II binder peptides throughout its sequence. The maximum and minimum scores of IFN-γ inducer peptides were 2.2 and 0.3, respectively. Furthermore, the graph of the humoral and cellular response of the mammalian immune system against the PspA_1-5c+p_ vaccine administrations using the C-ImmSim server showed an increase in the IgM titer characterizing a primary response. Following injection of the booster dose of PspA_1-5c+p_, an increase in B cell populations, isotype switching, and the formation of memory cells as well as an increase in immunoglobulin expression (IgG1 + IgG2, IgM, and IgG + IgM) were observed as secondary and tertiary reactions. In addition, an increase in Th (helper) and TC (cytotoxic) cells with memory development, natural killer cells, and dendritic cell responses was found. High levels of macrophage activity are also identified. In parallel, the immune simulation also showed that IFN-γ and IL-2 production were stimulated after immunization, resulting in increased macrophage activity. IL-10 as an immunosuppressive cytokine and the secreted factor by established Th2 cells was also found (Fig. 10). The results of the IL-4pred, IL-10pred, and IFNepitope predictions were consistent with the results of the C-ImmSim simulation and showed that the PspA_1-5c+p_ construct potentially increases the production of both cytokines in both humoral and cellular immune pathways.

### Expression, purification, and confirmation of recombinant PspA_1-5c+p_

**Fig. 11:**
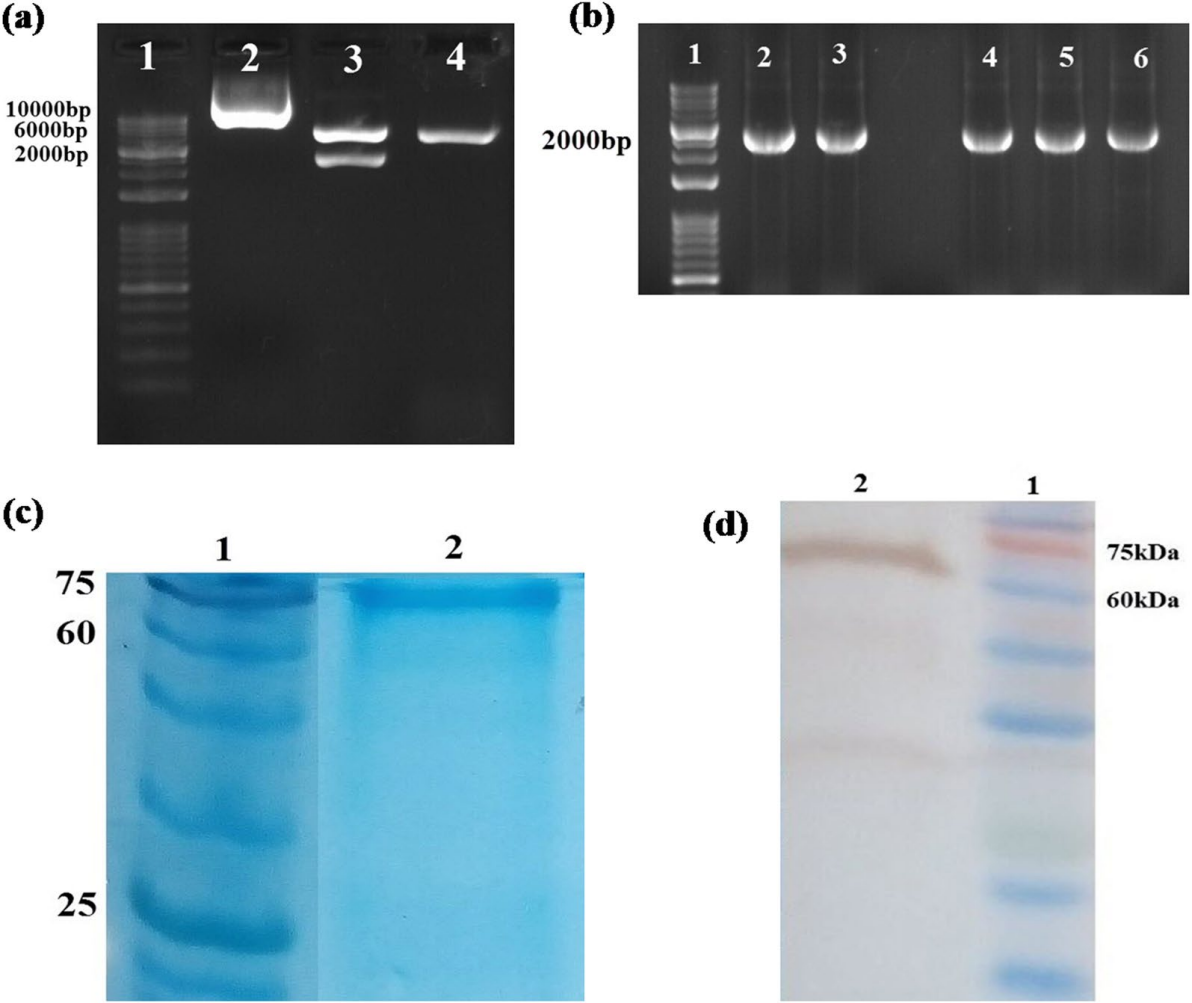
**a** Double digestion of recombinant plasmid pET28a-PspA_1-5c+p_ with *Nco*I and *Xho*I restriction enzymes. Lane1. 1kB DNA Ladder marker, 2. Undigested plasmid, 3. Double digestion of the plasmid with *Nco*I and *Xho*I, resulting in two bands of the PspA_1-5c+p_ (~ 1826 bp) and pET28a (~ 5639 bp). 3. Monodigestion of plasmid with *Nco*I. **b** Colony PCR on transformed *E. coli BL21* colonies with universal T7 primers. Lane1. 1kB DNA Ladder marker, lanes 2 to 6. Positive colonies with a band size of the PspA_1-5c+p_ (~ 1826 bp). **c** Purification of recombinant PspA_1-5c+p_ protein by Ni–NTA chromatography. **d** Western blot analysis of the purified recombinant PspA_1-5c+p_

The positive transformed *E. coli BL21* clones containing recombinant plasmid were confirmed using restriction enzyme digestion with *Nco*I and *Xho*I (resulting in two bands with sizes of approximately 1826 bp and 5369 bp) and colony PCR with universal T7 primers (resulting in a single band with the size of about 1826 bp) (Fig. 11a, b). Then, the expression of the recombinant PspA_1-5c+p_ protein was carried out with IPTG and analyzed with 12% SDS-PAGE. The results of the SDS-PAGE revealed the presence of a 67 kDa recombinant PspA_1-5c+p_ band, as expected by MW calculations. The purification of the recombinant PspA_1-5c+p_ was applied by Ni–NTA affinity chromatography under native conditions (Fig. 11c). In accordance with the solubility bioinformatics analysis of the PspA_1-5c+p_ construct, the experimental analysis showed that the PspA_1-5c+p_ construct was soluble. The expression of the PspA_1-5c+p_ construct was verified by the western blot on PspA_1-5c+p_ using an anti-His tag antibody (Fig. 11d). The LAL test showed an imperceptible level of LPS (< 0.5 EU/ml) in PspA_1-5c+p_ solution.

### Assessment of immune responses

**Fig. 12:**
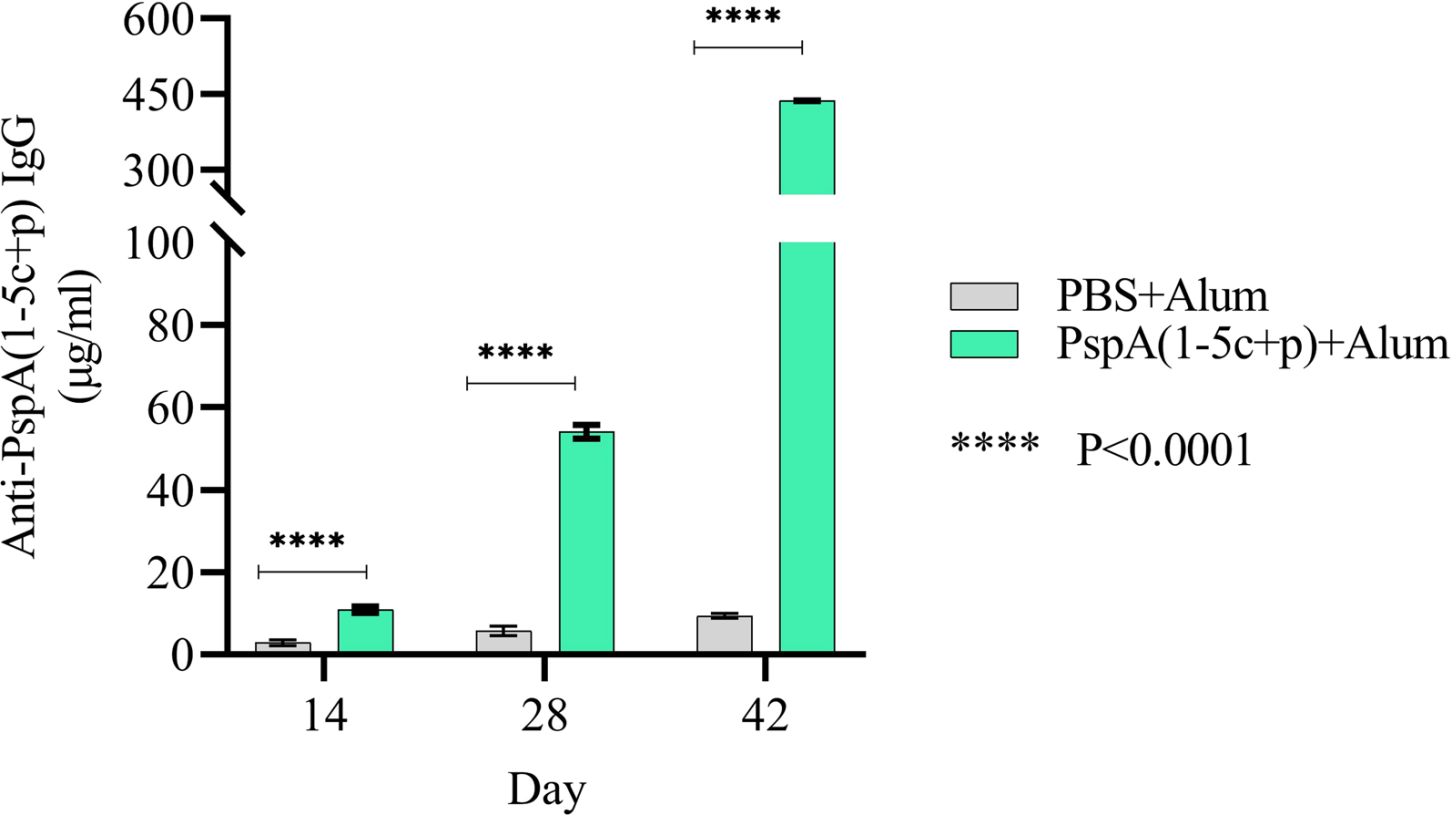
Evaluation of immune response in immunized mice. The booster effect of PspA_1-5c+p_ administration and specific IgG levels at different times of the injections were shown. A significant increase was observed in the mice immunized with PspA_1-5c+p_ at a dilution of 1:1000 compared with the control group. The error bar is representative of the mean ± standard deviation

Two weeks after the last immunization, the specific IgG level against the PspA_1-5c+p_ construct was analyzed using ELISA. The group of mice that were immunized with the PspA_1-5c+p_ construct and Alum revealed that the specific IgG level significantly increased compared to the control group (p < 0.0001) at different times of administration (Fig. 12). The results showed that the immunization of mice with this construct could stimulate the immune system response.

### Whole-cell ELISA analysis

**Fig. 13:**
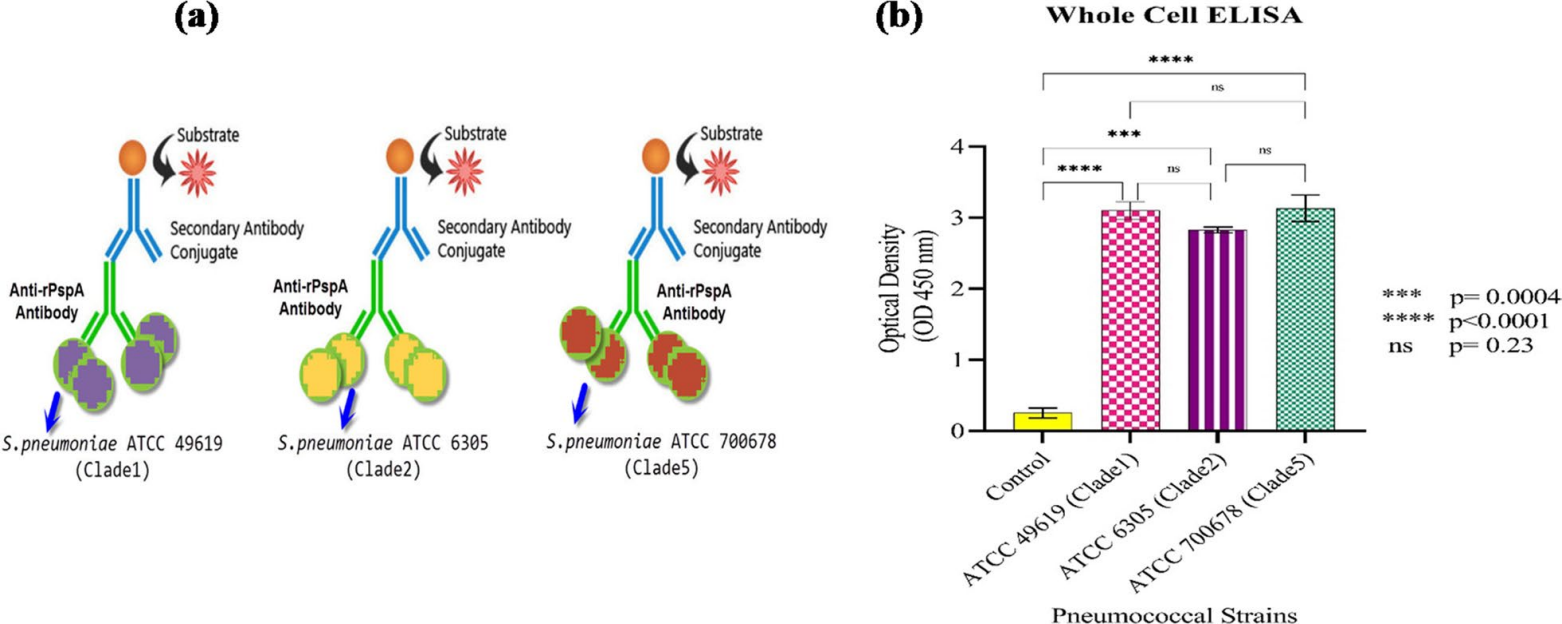
**a** Evaluation of cross-reactivity of anti-PspA_1-5c+p_ antibody using the Whole-cell ELISA. **b** The binding ability of anti-PspA_1-5c+p_ IgG against the surface of three pneumococcus strains, representing two families of PspA as cross-reactivity response. The error bar is representative of the mean ± standard deviation

The results of whole-cell ELISA analyzing the cross-reactivity and binding ability of anti-PspA_1-5c+p_ IgG showed that anti-PspA_1-5c+p_ IgG reacted strongly to the surface of all three pneumococcal strains (Fig. 13). No significant difference was seen between anti-PspA_1-5c+p_ antibody cross-reactivity optical densities against all three pneumococcus strains representing two PspA families (p-value = 0.2).

### SBA analysis

**Fig. 14:**
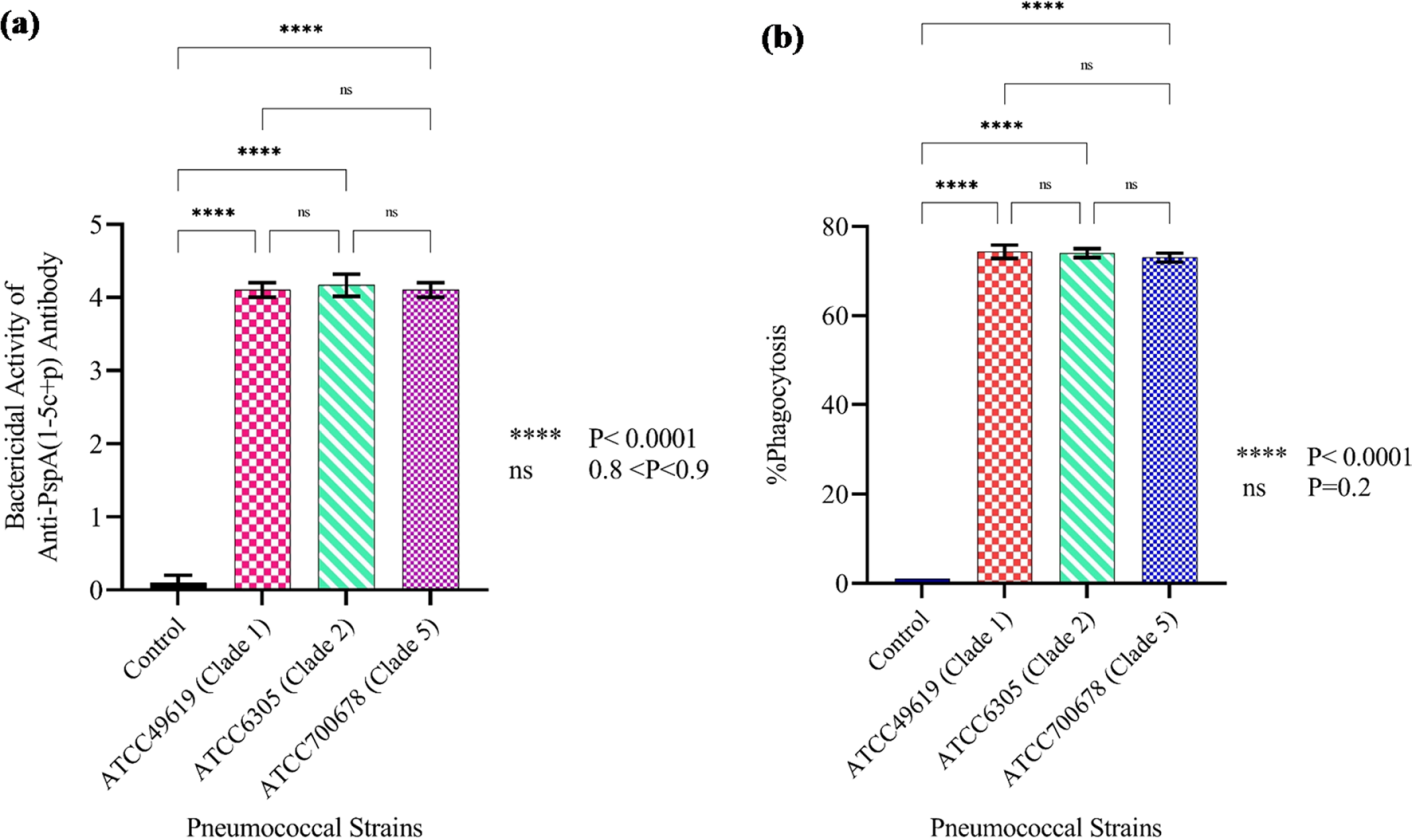
Evaluation of the functional activity of the anti-PspA_1-5c+p_ antibody. **a** The complement-mediated killing feature of anti-PspA_1-5c+p_ antibody against pneumococcal strains. **b** Phagocytosis-mediated killing feature of anti-PspA_1-5c+p_ antibody against pneumococcal strains. The error bar is representative of the mean ± standard deviation

The complement-mediated killing feature of the anti-PspA_1-5c+p_ antibody against three strains of pneumococcus, expressing two PspA families, was assessed using serial dilutions of the prepared serum up to 1:64. The results of the SBA are shown in Fig. 14a. The highest bactericidal activity was detected in 1:4 dilution. This dilution showed the ability of the anti-PspA_1-5c+p_ antibody to kill more than 50% of pneumococci compared to the control group. No significant difference was seen between the complement-mediated killing feature of the anti-PspA_1-5c+p_ antibody against three strains of pneumococcus expressing three clades of PspA (0.8 < p-value < 0.9). No bactericidal effects were reported in the negative controls.

### OPA analysis

The phagocytic killing capacity of mouse peritoneal macrophages and phagocyte cells exposed to anti-PspA_1-5c+p_ antibody revealed a significant increase compared to the control group (p < 0.0001) leading to a more than 50% reduction in the number of bacteria. In addition, no significant difference was found between the phagocytosis-mediated killing feature of the anti-PspA_1-5c+p_ antibody against pneumococcus strains ATCC6305, ATCC700678, or ATCC49619 (p = 0.2). No opsonic killing activity was observed in the PBS group. The data indicated that antibodies raised against PspA_1-5c+p_ act as a good opsonin for killing pneumococcus strains representing both PspA families (Fig. 14b).

## Discussion

A successful serotype-independent PspA-based vaccine against pneumococcus is a vaccine that comprises multiple immunological surface components with high cross-reactivity feature including various N-terminal domains of the PspA families [24, 66]. So, the purpose of designing this study was first to improve the PspA-based vaccine potency and efficacy using immunoinformatics tools as the first line of vaccine design. Then make experimentally a new construct with highly conserved and variable regions with high antigenic binding epitopes of B- and T-cell, with emphasis on cross-reactive regions of PspA N-terminal to evaluate the covering immune response against pneumococcal PspA clades.

To our knowledge, this is the first study on the immunoinformatics-based design of PspA families-based vaccines. We first modeled, refined, and validated the 3D structure of five different PspA clades using computational approaches. Then, we also predicted linear and conformational B-cell, and T-cell epitopes, especially at cross-reactive regions of PspA families 1 and 2 using various databases. According to B-cell epitope prediction servers (BCPred, IEDB, and Ellipro), the cross-reactive regions of each clade were analyzed. These regions had at least five B-cell epitope sequences of ~ 6–25mer in length with VaxiJen scores of 0.5 to 1. Some of the predicted epitopes had antigenicity scores of 2 to 3.3. The antigenicity score, surface accessibility, flexibility, hydrophilicity, beta-turn features, and conformational B-cell epitopes of predicted immunodominant regions were also considered suitable for designing cross-reactive PspA-based vaccines. As not all antibodies against PspA are protective; therefore, understanding which epitopes can elicit a protective response is critical [67]. So epitope mapping of PspA is one of the most widely used methods for identifying these epitopes. McDaniel et al. (1994) showed that the protection-eliciting regions of PspA were localized at 192–260 amino acid regions of PspA from the strain Rx1 using four of the nine monoclonal antibodies [68]. In accordance with McDaniel, we analyzed the B-cell epitopes of strain Rx1 as a clade 2 using immunoinformatics databases, and our results showed that the most predicted epitopes were located in the predicted region by McDaniel, which has thirteen sequences of 7–104 mer in length with VaxiJen scores of 0.5 to 1. Findings from McDaniel’s experimental study have the potential to support our predictions. Therefore, we also used this immunoinformatics prediction method for epitope mapping of other PspA clades. In agreement with Singh et al. [69] we used MHC-II binding epitopes prediction servers. Then the predicted epitopes for strain 435/96 (clade1) were compared with the predicted epitopes by Singh and showed almost similar results that could be considered for inducing IFN-γ and IL-4 production. So, we analyzed other PspA clades for MHC-II binding epitope prediction. Some predicted HTL epitopes were also predicted as B-cell epitopes, so we selected the immunodominant truncated CDR regions of each clade.

Mukerji et al. classified the proline-rich domain (PRD) of the PspA into three relatively distinct groups [21]. On the other hand, these PRD regions, especially the PKPEQP motif and non-proline block (NPB) sequence, can elicit protection against pneumococcal infection. As Daniels et al. indicated that when mice were immunized by group 2 PRD, they have been shown protection against the challenge test by a pneumococcal strain with group 3 PRD. These results showed cross-protection against epitopes shared by different groups of PRD [21, 70]. PRD group’s motifs have also been reported to be linear epitopes, and human antibodies can recognize all three PRD groups [21]. In completing the study by Mukerji et al. [21], we used the repetitive motif sequences from all three PRD groups and NPR sequence as a highly conserved and immunogenic domain in PRD of PspA to cover all diversity and cross-protection of the PRD groups. Finally, the designed construct has been named PspA_1-5c+p_ representing the CDR and proline regions of five PspA clades. We used the rigid linker EAAAK between each truncated domain to make the least interaction between domains and maintain the best three-dimensional structure and accessible B-cell conformational epitopes. According to the literature, many natural linkers have alpha-helical structures, which are stable and rigid spacers to keep a fixed distance that is used for separating the functional domains. Another advantage of rigid linkers compared to flexible linkers is that the flexible linkers lead to low expression yields with loss of biological activity[49].

The designed PspA_1-5c+p_ construct was assessed for its physicochemical characteristics. The PspA_1-5c+p_ construct was expected to be acidic in nature, depending on the theoretical isoelectric point. The aliphatic index (indicating thermostability) and grand average of hydropathicity (GRAVY) were estimated at 82.23 and -0.997, respectively. The negative GRAVY value means that the protein has a hydrophilic nature and may interact with water molecules. The *in-vivo* half-life, as an estimation of time for destroying half the amount of protein after synthesis in the cell, was estimated at 30, 20, and 10 h in mammalian, yeast, and *E. coli,* respectively. Although the instability index was computed at 40.12, which categorizes the protein as unstable (II of > 40 indicates instability), the experimental result of the recombinant PspA_1-5c+p_ expression and purification showed that this protein was stable [51]. The molecular weight of the PspA_1-5c+p_ construct was 67.93 kDa. The SDS-PAGE and western bot results of recombinant PspA_1-5c+p_ expression confirmed the estimated molecular weight of PspA_1-5c+p_ construct. It has been reported that proteins with a molecular weight of less than 100 kDa are suitable for vaccine design due to their easy expression and purification steps [46]. Therefore, this designed protein was an acceptable vaccine candidate. Also, the results of the codon adaptation index (CAI) and GC content of 0.84 and 42.97%, respectively, showed a good efficiency of the final vaccine transcription and translation in the *E. coli* host. So that, the Gibbs free energy after sequence optimization for PspA_1-5c+p_ construct mRNA was -445.5 kcal.mol^−1^, showing the lowest free energy and stable structure, and there was no observed unsuitable pseudo-knot or loop at 5’of mRNA. These computational results were confirmed with the expression of PspA_1-5c+p_ in *E. coli BL21* using 1 mM IPTG. The presence of a 67 kDa recombinant PspA_1-5c+p_ sharp band by 12% SDS-PAGE showed acceptable expression and codon optimization. The computationally predicted overexpression and soluble feature of PspA_1-5c+p_ using the SOLpro prediction were validated by purification of recombinant PspA_1-5c+p_ under the native condition in Ni–NTA affinity chromatography with a high concentration of 0.8 mg/ml. The expression of the PspA_1-5c+p_ construct was verified by the Western blot on PspA_1-5c+p_ using an anti-His tag antibody.

Understanding the secondary and tertiary structures of the target protein is critical to vaccine design. The secondary structure of PspA_1-5c+p_ contained 83.22% alpha-helix, 0.49% extended strand, and 16.28% random coil using the GOR V prediction server. It has been reported that the important shapes of “structural antigens” are natively unfolded protein regions and alpha-helical coiled-coil peptides. Both structural forms can be retreated into their native structure and therefore be identified by antibodies naturally induced in response to infection [55]. The PspA_1-5c+p_ 3D structure was modeled using the I-TASSER server. This server is one of the best and most widely used servers for designing three-dimensional protein structures. I-TASSER server uses the multiple threading alignments from PDB to identify structural templates and designs the 3D structures using repetitive fragment assembly simulations [71]. According to many recent papers which have cited to I-TASSER server for protein 3D structure predictions [72–75], the I-TASSER server was ranked as the No 1 server for protein structure prediction in recent community-wide CASP7, CASP8, CASP9, CASP10, CASP11, CASP12, CASP13, and CASP14 experiments. It was also ranked as the best for function prediction in CASP9. The server is in active development with the goal to provide the most accurate protein structure and function predictions using state-of-the-art algorithms [36]. Using structural refinement servers, we could improve the overall quality factor of the initial PspA_1-5c+p_ 3D model predicted by I-TASSER from 89.66% to 98.14%, and in the Ramachandran plot, disallowed region residues were reduced from 1.6% to 1.2% after the refinement process. Ramachandran plot also revealed that most of the residues are located in the favored and allowed regions (98.8%), demonstrating that the overall model quality is satisfactory. The structural refinement servers optimized the hydrogen-bonding network, minimized the atomic energy of the model, and improved the 3D structure by molecular dynamics simulation. In this study, the MD simulation was applied to verify the stability and flexibility of the structure of the designed PspA_1-5c+p_ protein. Analysis of the MD simulation trajectory revealed that the designed structure of the PspA_1-5c+p_ reaches a stable state with low deviations from 50 to 85 ns. This can indicate the stability of the 3D structure. In addition, using the RMSF plot, we found that the C-terminal of PspA_1-5c+p_ protein is the fluctuating region of the protein. During the simulation, the fluctuation of this region occurred around 0.7 nm. Nonetheless, the rest of the protein had a fluctuating value of less than 0.35 nm. These residues (C-terminus region of the protein) have more freedom of action in the environment due to the coil structure. Furthermore, the ClusPro and DimPlot results of PspA_1-5c+p_ and HLA-DRB1*01:01 (the most common binding allele in the Iran population [46]) docking complex showed the lowest energy binding of -744.3 kcal.mol^−1^ and 64 cluster members indicating good binding affinity and coupling of this protein with human MHCII via sixteen hydrogen bonds and six salt bridges. However, in order to improve and examine the precise interaction between the protein and HLA-DRB1*01:01, the docking between the T-cell epitope placed in the groove of HLA-DRB1*01:01 chains with the T lymphocyte receptor (TCR) [76] or docking of the human ternary complex of the T-cell receptor, peptide-MHCII molecule, and CD4 are recommended [77]. Since the PspA has a lactoferrin binding domain in the CDR region[59], to furthermore validation of the 3D structure of the modeled PspA_1-5c+p_, we docked the PspA_1-5c+p_ protein with human lactoferrin N-lobe (HLF). We demonstrated the PspA_1-5c+p_ protein can be attached to HLF molecules effectively via both regions representing PspA Families 1 and 2 in PspA_1-5c+p_ protein with the lowest energy binding of -1128.9 and -987.2 kcal.mol^−1^ and maximum cluster members of 58 and 80, respectively. In coordination with the docked control molecule (PDB id: 2PMS) [59], in two models of the PspA_1-5c+p_-HLF docked complex, most residues of HLF that have been in contact with CDR residues include Arg4, Arg5, Arg25, Arg28, Arg31, Arg40, Gln14, Gln24, and lys39. It has been reported that the negatively charged surface of PspA helices can interact with the highly cationic lactoferricin moiety of lactoferrin and inhibit its bactericidal effect against *pneumococci*. Our results were in line with the study conducted by Senkovich et al. and could show a good 3D structure of the CDR region in the PspA_1-5c+p_ vaccine formulation that could bind to HLF correctly. Senkovich et al. also suggested that inhibition of this interaction using small molecules or antibodies may permit lactoferrin’s natural bactericidal effects to preserve the host from pneumococcal colonization and infection and can be used for designing therapeutic strategies for the prevention and treatment of pneumococcal diseases [59]. Therefore, further studies can be performed to evaluate the binding of antibodies generated against PspA_1-5c+p_ to PspA on the surface of the different pneumococcal strains in the presence of the labeled human lactoferrin.

The results of predicting the conformational B-cell epitopes of the PspA_1-5c+p_ construct showed that after designing the structure, the conformational B-cell epitopes of each clade with a score of > 0.5 could be identified by the ElliPro server. These results can be indicated by the high potential of the PspA_1-5c+p_ to stimulate humoral immunity with the help of antibodies. One of the first steps in confirming a vaccine candidate is immunoreactivity detection using the serological test. According to the antigenicity score of 0.77 for the final PspA_1-5c+p_ construct from the Vaxijen server, this protein was considered a good antigen to stimulate the immune system. The experimental results confirmed and validated the computational antigenicity analysis of this protein. This protein was able to raise anti-PspA_1-5c+p_ IgG titers in immunized mice with PspA_1-5c+p_ construct compared to the control group (p < 0.0001) at different times of administration (Fig. 12). In addition, using immunoinformatics predictions, PspA_1-5c+p_ was considered a non-toxic, and non-allergen. So that, in the experimental results, this protein provided a very good and effective immunological response without causing any allergenicity or toxicity in the animal model. So that, after injection of the PspA_1-5c+p_ construct, we did not observe any increase in body temperature, weight loss, allergic reaction, sensitivity, or restlessness in the animal model. As in past studies conducted on the PspA protein, there were no reports of any deleterious nature of PspA. Sanofi Pasteur has also studied phase 1 of the clinical trial of PspA [9, 74, 75]. In this study, we demonstrated that anti-PspA_1-5c+p_ IgG reacted strongly with no significant difference (p-value = 0.2) against the surface of all three pneumococcal strains representing both PspA families. These results can indicate the high coverage of the cross-reactivity and binding ability of the anti-PspA_1-5c+p_ IgG among different used PspA clades, and cover the limitation of different cross-reaction levels in the PspA-based construct designed so far. In this context, Akbari et al. demonstrated that an antibody against the PspAB_1-5_ antigen containing the single B region from all clades compared to PspA_4_ABC could increase the cross-reactivity against pneumococcus strains representing Clades 1, 2, and 5. However, the strong binding ability of the anti-PspAB_1-5_ antibody was against strain ATCC 6305 (Clade 2) with an optical density of ~ 2.1. Although, they suggested that for the construction of a PspA-based vaccine, the B region from all clades should be included [7] but is not sufficient due to the significant difference observed between optical densities of the cross-reactivity ability of the anti-PspAB_1-5_ antibody against all three pneumococcus strains [7]. In this study, no different cross-reactivity ability of the anti-PspA_1-5c+p_ antibody was seen against two PspA families. In contrast to Akbari et al., our whole-cell ELISA results showed the optical density of the cross-reactivity ability of the anti-PspA_1-5c+p_ antibody was the same between three stains (Clades 1, 2, and 5) and increased to 3. This increase in the tendency of anti-PspA_1-5c+p_ antibody to bind to the bacterial surface may be due to two factors: the use of all cross-reactive truncated domain of CDRs together with highly conserved NPB region and using repetitive proline-rich motifs that cover the diversity of each clade. This study was also able to solve problems related to cross-reactivity differences in the studies of other research that used the various recombinant PspA proteins consisting of N-terminal and proline-rich regions from two PspA families or each region alone [23, 25, 78–80].

We also applied the Opsonophagocytosis test to assess the in vitro potential protective effects of PspA-based vaccines against pneumococcus strains representing both PspA families. The gold standard *in-vitro* test for assessing the polysaccharide-base pneumococcal vaccine effectiveness is the Opsonophagocytosis assay [16, 81]. Opsonophagocytosis is thought to be considered an important function in the host defense for the elimination of pneumococci. This process is started by complement activation in the presence of antibodies that are attached to the surface of pneumococci. Then, using phagocytic cells, pneumococci are swallowed and killed [16, 81]. The results showed that the anti-PspA_1-5c+p_ antibodies act as a good opsonin for killing pneumococcal strains and can attach to the native protein from each PspA clade on the surface of pneumococcal strains representing both PspA families.

We also analyzed the complement-mediated killing activity of anti-PspA_1-5c+p_ antibody as a serum bactericidal assay against three strains of pneumococcus, expressing two PspA families. The highest bactericidal activity was detected at a 1:4 dilution in order to kill more than 50% of pneumococci compared to the control group. No significant difference was seen between the antibody’s activities against three strains of pneumococcus*.* These results suggest that this antibody not only has a high titer with strong and uniform cross-reactivity coverage against three pneumococcal strains but also has high bioactivity for pneumococcal clearance using complement or phagocytic cells. Goulart et al. reported that the level of complement-mediated antibody-dependent phagocytosis depends on the similarity between anti-PspA antibodies and PspA that are expressed on the pneumococcal surface [80].

In addition, according to immune simulation servers, PspA_1-5c+p_ was predicted to compose antigenic regions that have the potency to induce IL-4 and IL-10 cytokines. Furthermore, the PspA_1-5c+p_ construct was predicted to have many IFN-γ inducing MHC class II binding peptides throughout its sequence. These bioinformatics results showed that PspA_1-5c+p_ might induce both humoral and cellular immune pathways. Overall, these results show the success of the bioinformatics tool in designing a PspA-based vaccine candidate to cover the cross-reactivity of the vaccine candidate against all used PspA clades. As the efficacy and reliability of the immunoinformatics approach have been proven in a lot of pioneering work regarding the design and development of epitope-based vaccines [26, 27, 47, 82, 83]. Our results are in accordance with these studies. In this context, Ahmadi et al. designed a novel Hla-MntC-SACOL0723 fusion protein using immunoinformatics tools. They then showed that this fusion protein could elicit high specific IgG titer with high opsonophagosytosis’s killing activity against *S. aureus* resulting in a decrease in the bacterial burden in the spleen and kidneys [47]. Hasanzadeh et al. also demonstrated that the computational design of their epitope‑based vaccine candidate could induce immune responses and provide high potency in the protection of the urinary tract against uropathogenic *Escherichia coli* (UTEC) [83].

The limitation of this study was the lack of access to standard pneumococcal strains expressing other clades of PspA for assessing the full cross-reactive feature of the anti-PspA_1-5c+p_ IgG. In the future, we will resolve the mentioned limitation and also analyze the profile of subclasses of specific IgG1 and IgG2a against PspA_1-5c+p_ construct immunization, levels of the IL-4 and IFN- γ cytokines, and the protection ability of this construct in immunized groups against pneumococcal infections to confirm our computational immune simulation results.

## Conclusion

Our experimental data revealed that immunoinformatics helps us to design protective serotype-independent vaccine candidates. Experimental assessments on three clades of PspA showed promising results with a strong cross-reactivity feature that should be further investigated in vitro and in vivo experiments with other pneumococcal clades to confirm the full cross-reactivity and cross-protection.

## Supplementary Information

**Additional file 1: Figure S1.** The schematic results of IEDB server for antigenicity, surface accessibility, flexibility, hydrophilicity, beta turn, linear and continuous predicted epitope analysis in PspA proteins. **Figure S2.** 3D modeling and validation of PspA clades. **Figure S3. **Graphical representation of features of secondary structure of the final pspA_1-5C+P_ construct sequence using PSIPRED server. **Table S1.** PspA candidates accession numbers and characteristics. **Table S2.** Predicted linear B-cell epitopes for PspA proteins using BCPred, IEDB, and Ellipro servers. **Table S3.** Assessment of refined and validated scores for 3D modelling of PspA clades structures. **Table S4. **Predicted conformational B-cell epitopes for PspA clades using Ellipro server. **Table S5.** Predicted helper T-cell epitopes for PspA Proteins using IEDB server (Percentile Rank ≤ 20). **Table S6.** Predicted helper T-cell epitopes for PspA proteins using RANKPEP server. **Table S7.** Predicted helper T-cell epitopes for PspA Proteins with IC50 value ≤ 100 (nM) using MHCPred server. Table S8. The best IL4 inducing analog/peptide from PspA_1-5c+p_ construct.

## Data Availability

Not applicable.

## Abbreviations

PspA: Pneumococcal surface protein A
IPD: Invasive pneumococcal disease
PPV: Pneumococcal protein-based vaccine
CDR: Clade-defining region
PRD: Proline-rich domain
NPB: Non-proline block
NCBI: National Center for Biotechnology Information
SVM: Support vector machine
PDB: Protein data bank
C-score: Confidence score
RMSD: Root Mean Square Deviation score
TM-score: Template modeling score
HTL: Helper T-cell
HLF: Human lactoferrin
PSSM: Position-specific score matrix
IPTG: Isopropyl-β-D Thiogalactopyranoside
LB: Luria–Bertani broth
LAL: Limulus amebocyte lysate
TMB: Tetramethylbenzidine
ELISA: Enzyme-linked immunosorbent assay
SBA: Serum Bactericidal Assay
OPK: Opsonophagocytic killing activity
ANOVA: Analysis of variances
GRAVY: Grand average of hydropathicity
MW: Molecular weight
pI: Isoelectric point
II: Instability index
CAI: Codon adaptation index
MD: Molecular dynamic
