# Supplementary material for: In-silico design and evaluation of an epitope-based serotype-independent promising vaccine candidate for highly cross-reactive regions of pneumococcal surface protein A
Source: J Transl Med. 2023 Jan 10;21:13. doi: 10.1186/s12967-022-03864-z (PMC9830136; doi:10.1186/s12967-022-03864-z)
Supplement: Supplementary file 1 — Additional file 1: Figure S1. The schematic results of IEDB server for antigenicity, surface accessibility, flexibility, hydrophilicity, beta turn, linear and continuous predicted epitope analysis in PspA proteins. Figure S2. 3D modeling and validation of PspA clades. Figure S3. Graphical representation of features of secondary structure of the final pspA1-5C+P construct sequence using PSIPRED server. Table S1. PspA candidates accession numbers and characteristics. Table S2. Predicted linear B-cell epitopes for PspA proteins using BCPred, IEDB, and Ellipro servers. Table S3. Assessment of refined and validated scores for 3D modelling of PspA clades structures. Table S4. Predicted conformational B-cell epitopes for PspA clades using Ellipro server. Table S5. Predicted helper T-cell epitopes for PspA Proteins using IEDB server (Percentile Rank ≤ 20). Table S6. Predicted helper T-cell epitopes for PspA proteins using RANKPEP server. Table S7. Predicted helper T-cell epitopes for PspA Proteins with IC50 value ≤ 100 (nM) using MHCPred server. Table S8. The best IL4 inducing analog/peptide from PspA1-5c+p construct. [file 12967_2022_3864_MOESM1_ESM.docx]

**
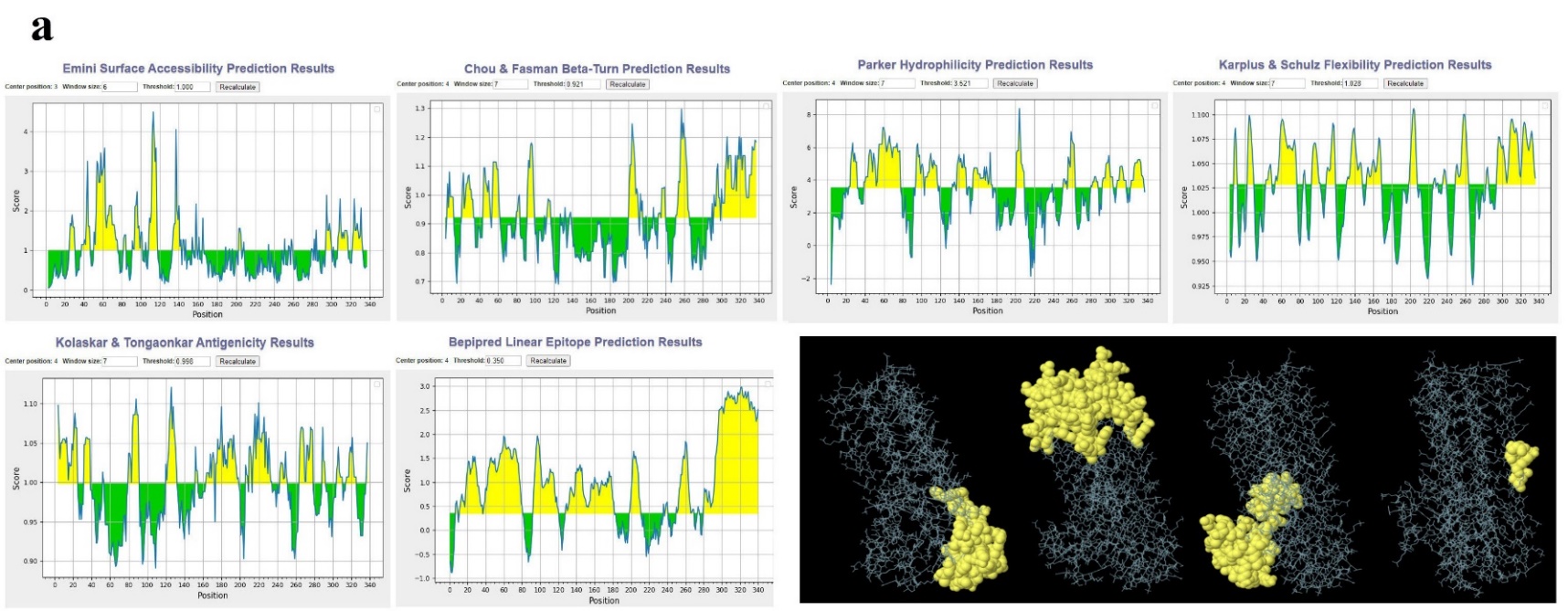
Additional file 1: Figures**


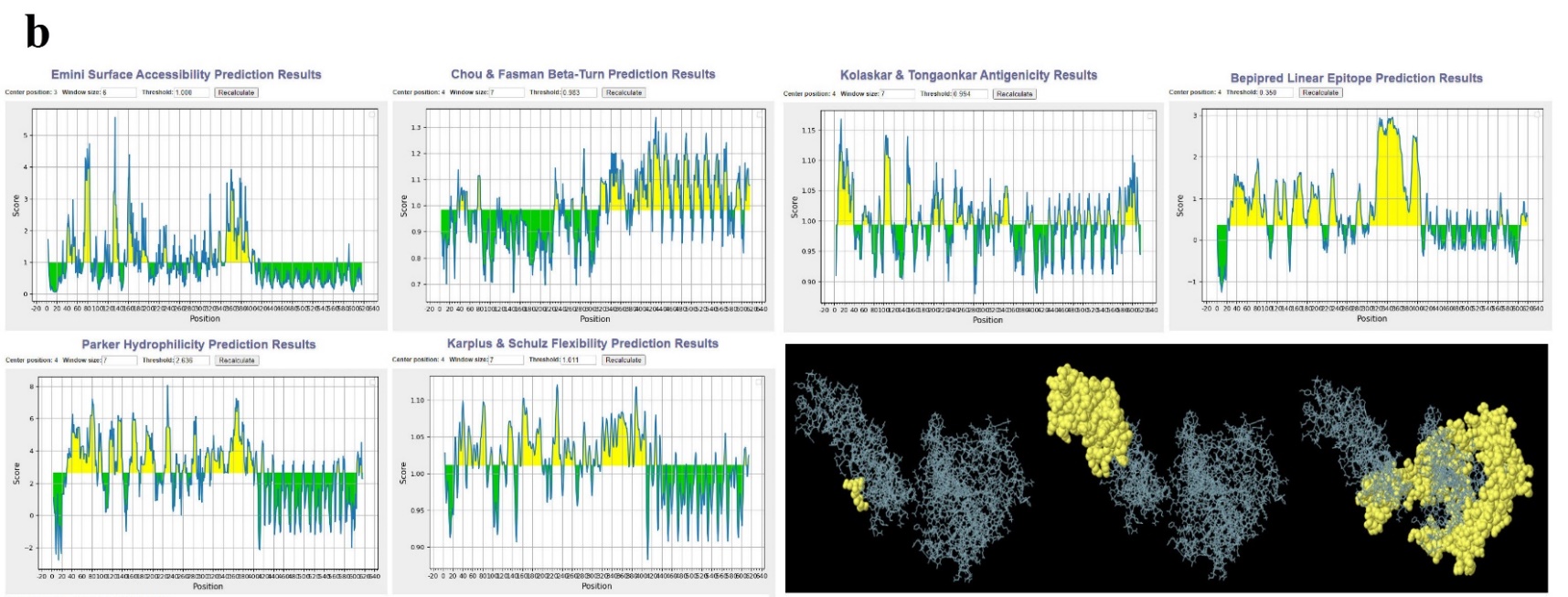


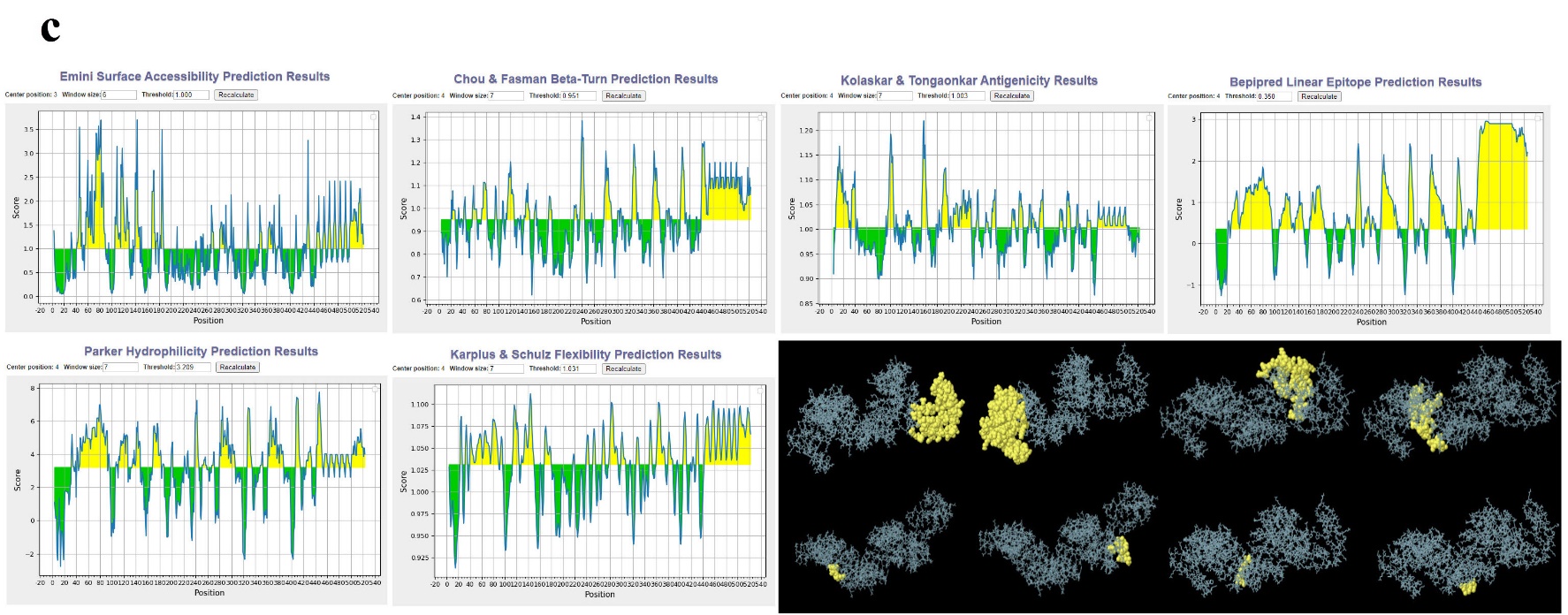


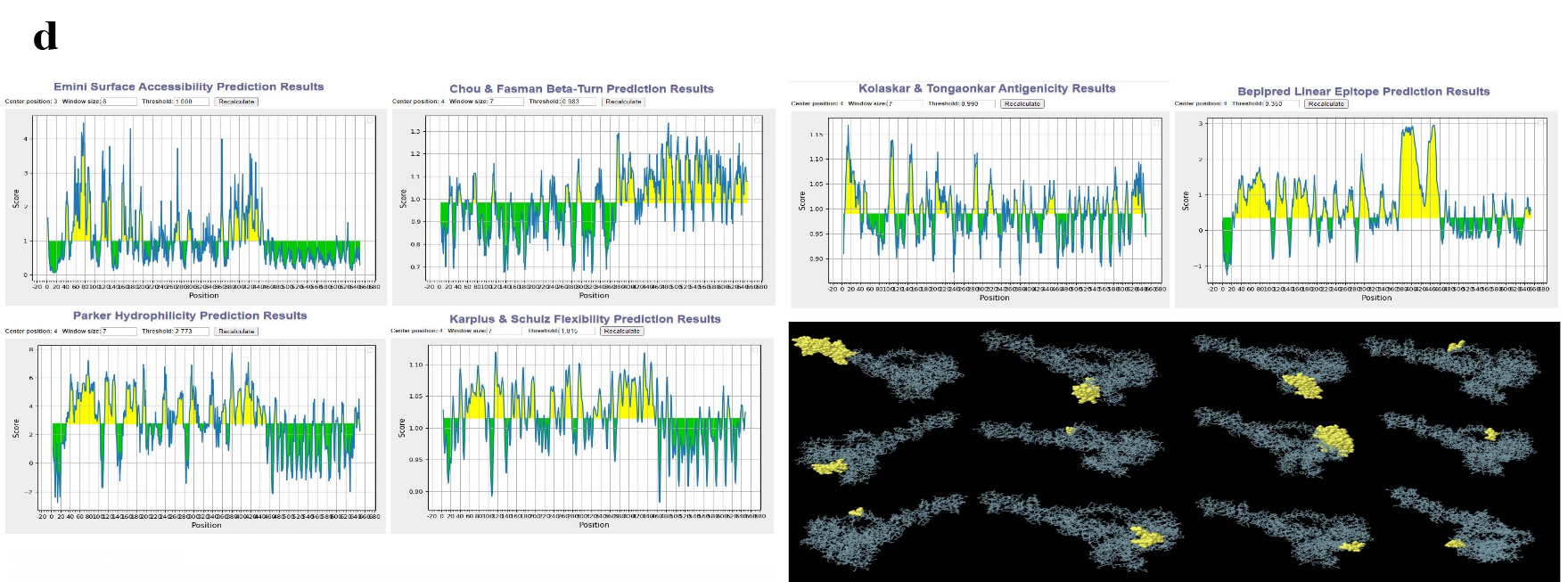


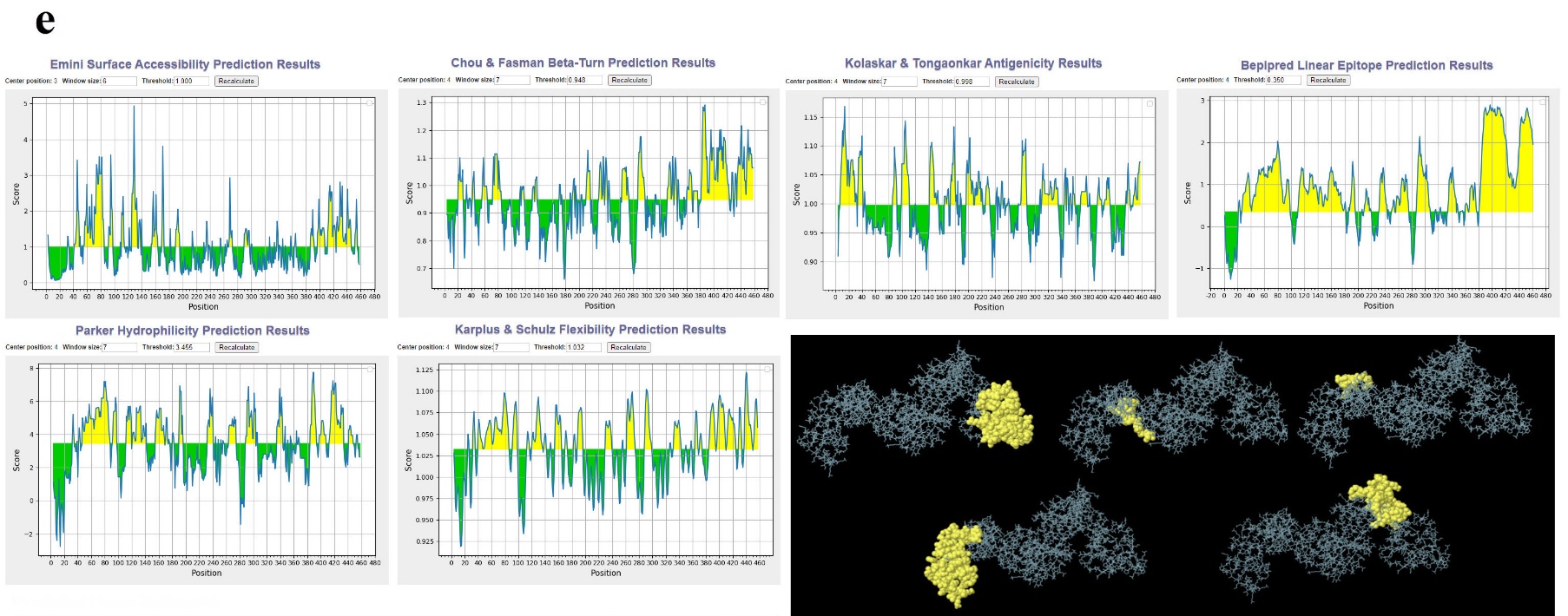


Figure S1: The schematic results of IEDB server for antigenicity, surface accessibility, flexibility, hydrophilicity, beta turn, linear and continuous predicted epitope analysis in PspA proteins. a) PspA family 1 (clade1), b) PspA family 1 (clade2), c) PspA family 2 (clade3), d) PspA family 2 (clade4), e) PspA family 2 (clade5). The residues with scores above the threshold (default value is 0.5) are predicted to be part of an epitope and are colored yellow on the graph (where y-axes depicts residue scores and x-axes residue positions in the sequence).


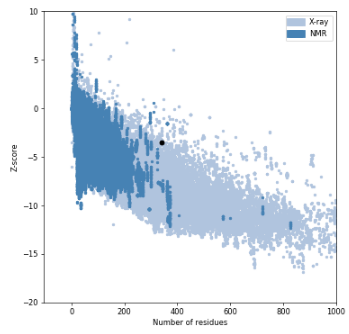

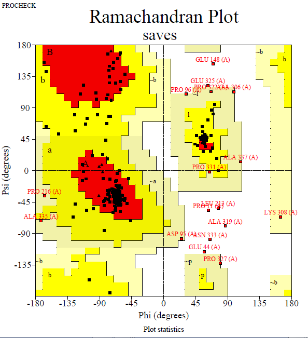

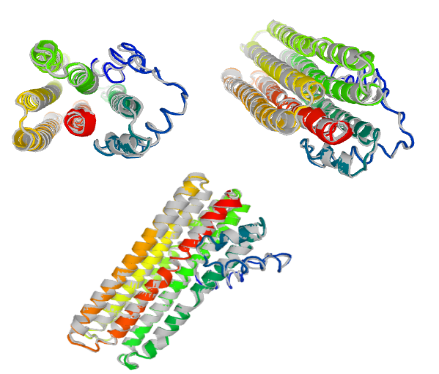


**(c)**

**(b)**

**(a)**

**(1)**

**
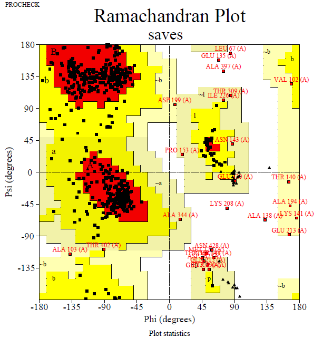
**
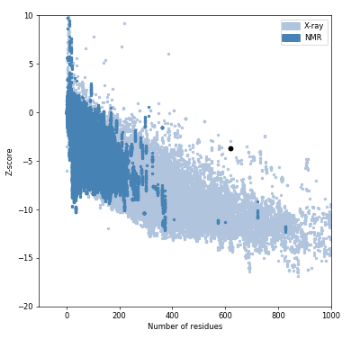
**
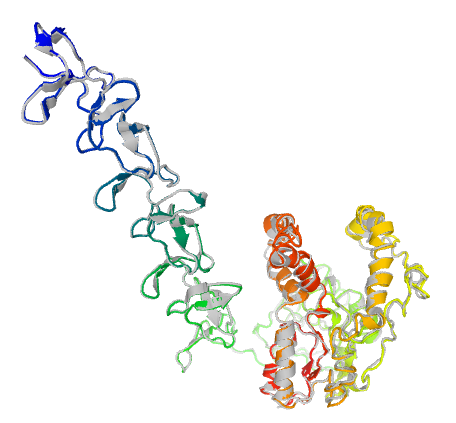
**

**(2)**


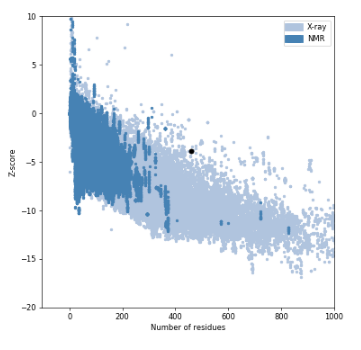

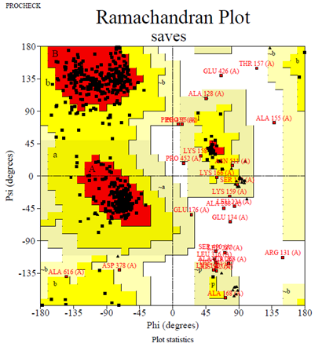


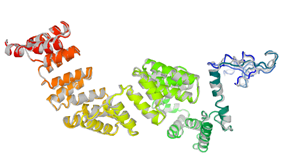


**(3)**


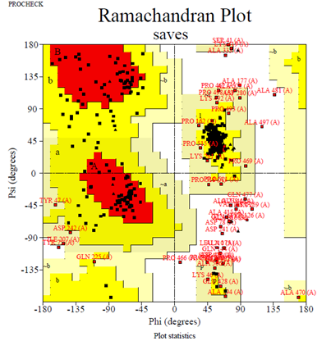

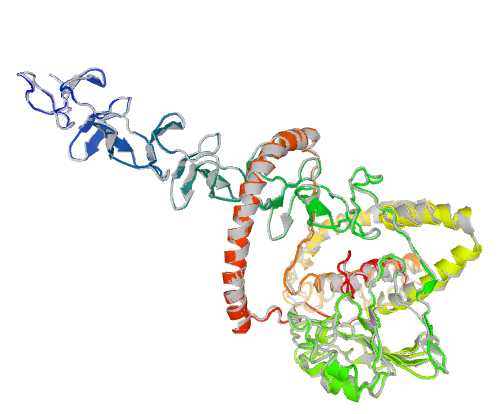


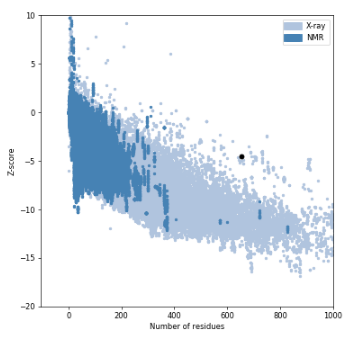


**(4)**


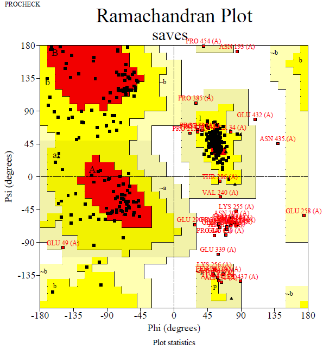


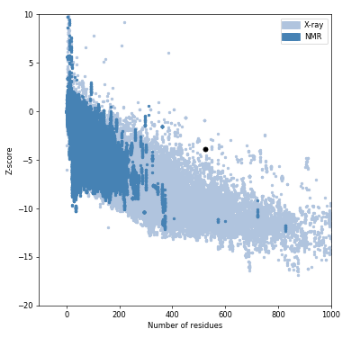
**
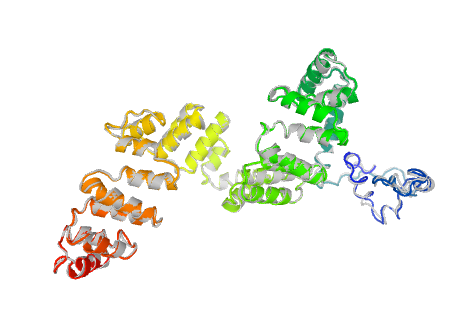
**

**(5)**

Figure S2. 3D modeling and validation of PspA clades. The numbers on the left of the figures represent respective PspA clade number. Columns a, b and c columns indicate the predicted 3D modeling by I-TASSER, Ramachandran plot, and ProSA validation for each PspA clades structure, respectively.


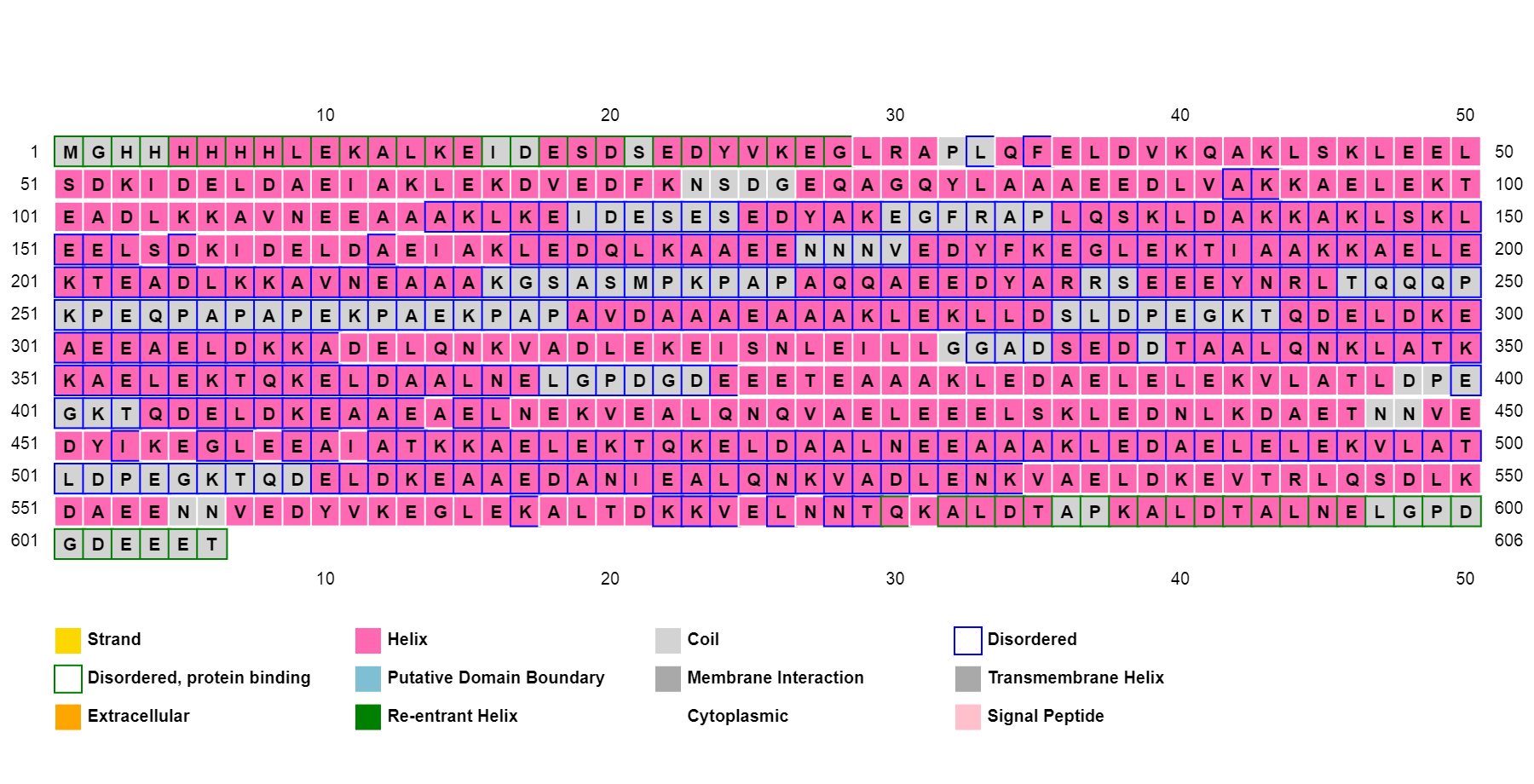


Figure S3. Graphical representation of features of secondary structure of the final pspA_1-5C+P_ construct sequence using PSIPRED server. The protein is predicted to comprise alpha helices (83.22%), beta strands (0.49%) and coils (16.28%). The alpha helix residues are pink, the beta strand residues are yellow and the coil residues are gray.

**Human Lactoferrin (HLF)**

**Additional file 1: Tables**

Table S1. PspA candidates accession numbers and characteristics.

| Pneumococcal  Strain | PspA Family  Type | VaxiJen Score | Amino acid sequence | Trans-membrane Helices | Sub-cellular Localization | Signal Peptide | Accession Number (GeneBank) | Accession Number (Uniprot) |
| --- | --- | --- | --- | --- | --- | --- | --- | --- |
| St 435/96 | Family 1 (Clade1) | 0.8250 | ILGAGFVTSQPTFVRAEEAPVASQSKAEKDYDAAVKKYEAAKKEYEDGKAAQKKYEDDQKKTEEKAEEERKASEEEQAANLKYQQELVKYIRENDPTKKAEAKKAMDEAEKEYKKKQTEFAEVRAKVIPSAEELKKTRQKAEEAKAKEAELTKKVEEAEKKVTEAKQKVDAEHAEEVAPQAKIAELEHEVQKLEKALKEIDESDSEDYVKEGLRAPLQFELDVKQAKLSKLEELSDKIDELDAEIAKLEKDVEDFKNSDGEQAGQYLAAAEEDLVAKKAELEKTEADLKKAVNEPEKPAEETPAPAPKPEQPAEQPKPAPAPQPEKPAEEPENPASAPQP | 0 | Cytoplasmic | - | **AAL92492.1** | **Q8KQK5** |
| RX1 | Family 1 (Clade2) | 0.5847 | MNKKKMILTSLASVAILGAGFVASQPTVVRAEESPVASQSKAEKDYDAAKKDAKNAKKAVEDAQKALDDAKAAQKKYDEDQKKTEEKAALEKAASEEMDKAVAAVQQAYLAYQQATDKAAKDAADKMIDEAKKREEEAKTKFNTVRAMVVPEPEQLAETKKKSEEAKQKAPELTKKLEEAKAKLEEAEKKATEAKQKVDAEEVAPQAKIAELENQVHRLEQELKEIDESESEDYAKEGFRAPLQSKLDAKKAKLSKLEELSDKIDELDAEIAKLEDQLKAAEENNNVEDYFKEGLEKTIAAKKAELEKTEADLKKAVNEPEKPAPAPETPAPEAPAEQPKPAPAPQPAPAPKPEKPAEQPKPEKTDDQQAEEDYARRSEEEYNRLTQQQPPKAEKPAPAPKTGWKQENGMWYFYNTDGSMATGWLQNNGSWYYLNSNGAMATGWLQYNGSWYYLNANGAMATGWAKVNGSWYYLNANGAMATGWLQYNGSWYYLNANGAMATGWAKVNGSWYYLNANGAMATGWLQYNGSWYYLNANGAMATGWAKVNGSWYYLNANGAMATGWVKDGDTWYYLEASGAMKASQWFKVSDKWYYVNGLGALAVNTTVDGYKVNANGEWV | 1 | Extracellular | Sec/SPI  (Cleavage site between pos. 31 and 32) | **AAA27018.1** | **Q54972** |
| EF3296 | Family 2 (Clade3) | 0.7792 | MNKKKMILTSLASVAILGAGLVTSQPTFVRAEESPQVVEKSSLEKKYEEAKAKADTAKKDYETAKKKAEDAQKKYEDDQKRTEEKARKEAEASQKLNDVALVVQNAYKEYREVQNQRSKYKSDAEYQKKLTEVDSKIEKARKEQQDLQNKFNEVRAVVVPEPNALAETKKKAEEAKAEEKVAKRKYDYATLKVALAKKEVEAKELEIEKLQYEISTLEQEVATAQHQVDNLKKLLAGADPDDGTEVIEAKLKKGEAELNAKQAELAKKQTELEKLLDSLDPEGKTQDELDKEAEEAELDKKADELQNKVADLEKEISNLEILLGGADPEDDTAALQNKLAAKKAELAKKQTELEKLLDSLDPEGKTQDELDKEAEEAELDKKADELQNKVADLEKEISNLEILLGGADSEDDTAALQNKLATKKAELEKTQKELDAALNELGPDGDEEETPAPAPQPEQPAPAPKPEQPAPAPKPEQPAPAPKPEQPAPAPKPEQPAPAPKPEQPAKPEKPAEEPTQPEKPATPKT | 1 | Cytoplasmic | Sec/SPI  (Cleavage site between pos. 31 and 32) | **AAF27712.1** | **Q9LAX9** |
| EF5668 | Family 2 (Clade4) | 0.6102 | MNKKKMILTSLASVAILGAGFVASSPTFVRAEEAPVANQSKAEKDYDAAVKKSEAAKKDYETAKKKAEDAQKKYDEDQKKTEAKAEKERKASEKIAEATKEVQQAYLAYLQASNESQRKEADKKIKEATQRKDEAEAAFATIRTTIVVPEPSELAETKKKAEEATKEAEVAKKKSEEAAKEVEVEKNKILEQDAENEKKIDVLQNKVADLEKGIAPYQNEVAELNKEIARLQSDLKDAEENNVEDYIKEGLEQAITNKKAELATTQQNIDKTQKDLEDAELELEKVLATLDPEGKTQDELDKEAAEAELNEKVEALQNQVAELEEELSKLEDNLKDAETNNVEDYIKEGLEEAIATKKAELEKTQKELDAALNELGPDGDEEETPAPAPQPEKPAEEPENPAPAPKPEKSADQQAEEDYARRSEEEYNRLTQQQPPKAEKPAPAPQPEQPAPAPKIGWKQENGMWYFYNTDGSMATGWLQNNGSWYYLNSNGAMATGWLQYNGSWYYLNANGAMATGWLQYNGSWYYLNANGAMATGWLQYNGSWYYLNANGDMATGWLQYNGSWYYLNANGDMATGWAKVHGSWYYLNANGSMATGWVKDGETWYYLEASGSMKANQWFQVSDKWYYVNGLGSLSVNTTVDGYKVNANGEWV | 1 | Extracellular | Sec/SPI  (Cleavage site between pos. 31 and 32) | **AAC62252.1** | **O34097** |
| ATCC 6303 | Family 2 (Clade5) | 0.7503 | MNKKKMILTSLASVAILGTGFVASSPTFVRAEESPQVVEKSSLEKKYEEAKAKADTAKKDYETAKKKAEDAQKKYDEDQKKTEDKAKAVKKVDEERQKANLAVQKAYVEYREAKDKASAEKKIEEAKRKQKEANKKFNEEQAKVVPEAKELAATKQKAEKAKKDAEVAKEKYDKAVQEVEVEKNKILEQDAENEKKIDVLQNKVADLEKGIAPYQNKVAELNKEIARLQSDLKDAEENNVEDYIKEGLEQAIADKKAELATTQQNIDKTQKDLEDAELELEKVLATLDPEGKTQDELDKEAAEDANIEALQNKVADLENKVAELDKEVTRLQSDLKDAEENNVEDYVKEGLEKALTDKKVELNNTQKALDTAPKALDTALNELGPDGDEEETPAPAPKPEQPAEQPKPAPAPKPEKTDDQQAEEDYARRSEEEYNRLPQQQPPKAEKPAPAPKPEQPVPAP | 1 | Cytoplasmic | Sec/SPI  (Cleavage site between pos. 31 and 32) | **AAF27715.1** | **Q9LAX6** |

Table S2. Predicted linear B-cell epitopes for PspA proteins using BCPred, IEDB, and Ellipro servers.

| PspA | position | Peptide | Score |
| --- | --- | --- | --- |
| Clade1 | 174-198  163-183  199-210  214-234  250-269  264-278  291-311 | AEEVAPQAKIAELEHEVQKLEKALK  TEAKQKVDAEHAEEVAPQAK  EIDESDSEDYVK  RAPLQFELDVKQAKLSKLEEL  KDVEDFKNSDGEQAGQYLAA  GQYLAAAEEDLVAKK  AVNEPEKPAEETPAPAPKPE | 0.4  1.07  0.72  0.88  0.96  0.4  0.52 |
| Clade2 | 146-237  213-233  224-242  229-235  240-260  269-280  278-290  281-287  280-300  317-337  304-407 | RAMVVPEPEQLAETKKKSEEAKQKAPELTKKLEEAKAKLEEAEKKATEAKQKVDAEEVAPQAKIAELENQVHRLEQELKEIDESESEDYAKE  ENQVHRLEQELKEIDESESE  KEIDESESEDYAKEGFRAP  SESEDYA  RAPLQSKLDAKKAKLSKLEEL  AEIAKLEDQLKA  LKAAEENNNVEDY  AEENNNV  AAEENNNVEDYFKEGLEKTI  VNEPEKPAPAPETPAPEAPA  AELEKTEADLKKAVNEPEKPAPAPETPAPEAPAEQPKPAPAPQPAPAPKPEKPAEQPKPEKTDDQQAEEDYARRSEEEYNRLTQQQPPKAEKPAPAPKTGWKQE | 0.73  0.97  0.8  0.63  0.61  0.5  0.84  0.99  0.99  1  0.79 |
| Clade3 | 329-353  345-349  360-388  363-370  363-383  407-415  425-434  434-440  386-446 | TRLQSDLKDAEENNVEDYVKEGLEK  ELAKK  LDPEGKTQDELDKEAEEAELDKKADELQN  EGKTQDEL  EGKTQDELDKEAEEAELDKKADELQN  ADSEDDTAA  AELEKTQKEL  LDAALNE  DGDEEETPAPAPKPEQPAEQPKPAPAPKPEKTDDQQAEEDYARRSEEEYNRLPQQQPPKAE | 0.81  0.4  1.35  1.83  1.55  1.4  0.45  0.85  0.81 |
| Clade4 | 260-278  278-290  290-310  293-301  292-312  327-344  332-345  353-371  359-368  362-367  368-374 | AELATTQQNIDKTQKDLED  DAELELEKVLATL  LDPEGKTQDELDKEAAEAELN  EGKTQDELD  PEGKTQDELDKEAAEAELNE  LSKLEDNLKDAETNNVED  DNLKDAETNNVEDY  AIATKKAELEKTQKELDAA  AELEKTQKEL  EKTQKE  LDAALNE | 0.57  0.58  1.42  1.58  1.23  0.67  0.78  0.67  0.45  2.11  0.85 |
| Clade5 | 257-275  275-287  287-307  290-297  334-346  333-353  344-366  387-392  394-400 | AELATTQQNIDKTQKDLED  DAELELEKVLATL  LDPEGKTQDELDKEAAEDANI  EGKTQDEL  DLKDAEENNVEDY  SDLKDAEENNVEDYVKEGLE  AELAKKQTELEKLLDSLDPEGKT  GDEEET  APAPKPE | 0.57  0.58  1.32  1.83  1  0.99  0.59  3.3  1.03 |

Table S3. Assessment of refined and validated scores for 3D modelling of PspA clades structures.

| Model of PspA | ITASSER  C-Score | ITASSER  TM-Score | GDT-HA | RMSD | MolProbity | Clash score | Poor rotamers | Rama favored | ERRAT  Score | ProSA  Z-score |
| --- | --- | --- | --- | --- | --- | --- | --- | --- | --- | --- |
| Clade1 | -2.64 | 0.41±0.14 | 0.9059 | 0.566 | 2.568 | 28.1 | 1.1 | 87.6 | 82.53% | -3.73 |
| Clade2 | -2.94 | 0.38±0.13 | 0.8845 | 0.637 | 2.545 | 28.6 | 0.4 | 87.8 | 68.92% | -3.67 |
| Clade3 | -1.44 | 0.54±0.15 | 0.8796 | 0.632 | 2.655 | 22.8 | 0.5 | 73.9 | 51.12% | -3.83 |
| Clade4 | -2.45 | 0.43±0.14 | 0.8978 | 0.582 | 2.325 | 19.3 | 0.9 | 90.2 | 79% | -4.5 |
| Clade5 | -0.77 | 0.62±0.14 | 0.8973 | 0.598 | 2.885 | 23.4 | 2.1 | 75.6 | 40.92 | -3.89 |

Table S4. Predicted conformational B-cell epitopes for PspA clades using Ellipro server.

| PspA Clades | No. | Residues | Number of residues | Score |
| --- | --- | --- | --- | --- |
| Clade 1 | 1 | A:A40, A:A41, A:K42, A:K43, A:E44, A:Y45, A:E46, A:D47, A:G48, A:K49, A:A50, A:A51, A:Q52, A:K53, A:K54, A:Y55, A:E56, A:D57, A:D58, A:Q59, A:K60, A:K61, A:E63, A:E64, A:E133, A:K136, A:T137, A:R138, A:Q139, A:K140, A:A141, A:E142, A:E143, A:A144, A:K145, A:A146, A:K147, A:E148, A:A149, A:E150, A:L151, A:T152, A:K153, A:K154, A:E156, A:E157, A:K160, A:K230, A:E233, A:L234, A:S235, A:D236, A:K237, A:I238, A:D239, A:E240, A:L241, A:D242, A:A243, A:E244, A:I245, A:A246, A:K247, A:L248, A:E249, A:K250, A:E253 | 67 | 0.763 |
|  | 2 | A:I1, A:L2, A:G3, A:A4, A:G5, A:K82, A:Q85, A:E86, A:L87, A:V88, A:K89, A:Y90, A:I91, A:R92, A:E93, A:N94, A:D95, A:P96, A:T97, A:K98, A:K99, A:A100, A:E101, A:K103, A:K104, A:H173, A:A174, A:E175, A:V177, A:A178, A:P179, A:A181, A:K182, A:I183, A:A184, A:E185, A:L186, A:E187, A:H188, A:E189, A:V190, A:Q191, A:K192, A:L193, A:E194, A:K195, A:A196, A:L197 | 48 | 0.718 |
|  | 3 | A:A279, A:E280, A:E282, A:K283, A:T284, A:E285, A:A286, A:D287, A:L288, A:K289, A:K290, A:A291, A:V292, A:N293, A:E294, A:P295, A:K297, A:P298, A:A299, A:E300, A:E301, A:T302, A:P303, A:A304, A:P305, A:A306, A:P307, A:K308, A:P309, A:E310, A:Q311, A:P312, A:A313, A:E314, A:Q315, A:P316, A:K317, A:P318, A:A319, A:P320, A:A321, A:P322, A:Q323, A:P324, A:E325, A:K326, A:P327, A:A328, A:E329, A:E330, A:P331, A:E332, A:N333 | 53 | 0.687 |
|  | 4 | A:E67, A:E68, A:K71, A:E74 | 4 | 0.549 |
| Clade 2 | 1 | A:A521, A:T522, A:G523, A:W524, A:L525, A:Q526, A:Y527, A:N528, A:G529, A:S530, A:W531, A:Y532, A:Y533, A:M540, A:A541, A:T542, A:G543, A:W544, A:A545, A:K546, A:V547, A:N548, A:G549, A:S550, A:W551, A:Y552, A:Y553, A:L554, A:N555, A:A556, A:N557, A:G558, A:A559, A:M560, A:A561, A:T562, A:G563, A:W564, A:V565, A:K566, A:D567, A:G568, A:D569, A:T570, A:W571, A:Y572, A:Y573, A:L574, A:E575, A:A576, A:S577, A:G578, A:A579, A:M580, A:K581, A:A582, A:S583, A:Q584, A:W585, A:F586, A:K587, A:V588, A:S589, A:D590, A:K591, A:W592, A:Y593, A:Y594, A:V595, A:N596, A:G597, A:L598, A:G599, A:A600, A:L601, A:A602, A:V603, A:N604, A:T605, A:T606, A:V607, A:D608, A:G609, A:Y610, A:K611, A:V612, A:N613, A:A614, A:N615, A:G616, A:E617, A:W618, A:V619 | 93 | 0.833 |
|  | 2 | A:M1, A:N2, A:K3, A:K4, A:A23, A:S24, A:Q25, A:P26, A:T27, A:V28, A:V29, A:R30, A:A119, A:A120, A:K121, A:D122, A:A123, A:A124, A:K126, A:M127, A:I128, A:D129, A:E130, A:A131, A:K132, A:K133, A:R134, A:E135, A:E136, A:E137, A:A138, A:K139, A:T140, A:K141, A:F142, A:N143, A:T144, A:R146, A:A147, A:M148, A:V149, A:V150, A:P151, A:E152, A:P153, A:E154, A:Q155, A:L156, A:A157, A:E158, A:T159, A:K160, A:K161, A:S163, A:E164, A:E165, A:A166, A:K167, A:K169, A:A170, A:P171, A:E172, A:L173, A:T174, A:K175, A:K176, A:L177, A:E178, A:E179, A:A180, A:K181, A:A182, A:K183, A:L184, A:E185, A:E186, A:A187, A:E188, A:K190, A:A191, A:T192, A:E193, A:A194, A:K195, A:Q196, A:K197, A:V198, A:D199, A:A200, A:E201, A:E202, A:V203, A:A204, A:P205, A:Q206, A:A207, A:K208, A:I209, A:A210, A:E211, A:L212, A:E213, A:N214, A:Q215, A:V216, A:H217, A:R218, A:L219, A:E220, A:Q221, A:E222, A:L223, A:K224, A:E225, A:I226, A:D227, A:E228, A:S229, A:E230, A:S231, A:E232, A:D233, A:Y234, A:A235, A:K236, A:P242, A:L243, A:Q244, A:S245, A:K246, A:L254, A:L257, A:E258, A:E259, A:L260, A:S261, A:D262, A:A280, A:A281, A:E282, A:E283, A:K302, A:A326, A:P327, A:E328, A:T329, A:A334, A:E337, A:A342, A:P343, A:A344, A:P345, A:Q346, A:P347, A:A348, A:P349, A:A350, A:P351, A:K352, A:P353, A:E354, A:K355, A:P356, A:A357, A:E358, A:Q359, A:P360, A:K361, A:P362, A:E363, A:K364, A:T365, A:D366, A:D367, A:Q368, A:Q369, A:A370, A:E372, A:D373, A:Y374, A:A375, A:R376, A:S378, A:E379, A:E380, A:E381, A:Y382, A:N383, A:R384, A:L385, A:T386, A:Q387, A:Q388, A:Q389, A:P390, A:P391, A:K392, A:A393, A:E394, A:K395, A:P396, A:A397, A:P398, A:A399, A:P400, A:K401, A:T402, A:G403, A:W404, A:K405, A:Q406, A:E407, A:N408, A:G409, A:M410, A:W411, A:Y412, A:F413, A:Y414, A:N415, A:T416, A:D417, A:G418, A:S419, A:M420, A:A421, A:G438, A:A439, A:M440, A:A441, A:N455, A:A456, A:N457, A:G458, A:A459 | 235 | 0.669 |
|  | 3 | A:K466, A:N468, A:G469 | 3 | 0.579 |
| Clade 3 | 1 | A:L441, A:G442, A:P443, A:D444, A:G445, A:D446, A:E447, A:E448, A:E449, A:T450, A:P451, A:A452, A:P453, A:A454, A:P455, A:Q456, A:P457, A:E458, A:Q459, A:P460, A:A461, A:P462, A:A463, A:P464, A:K465, A:P466, A:E467, A:Q468, A:P469, A:A470, A:P471, A:A472, A:P473, A:K474, A:P475, A:E476, A:Q477, A:P478, A:A479, A:P480, A:A481, A:P482, A:K483, A:P484, A:E485, A:Q486, A:P487, A:A488, A:P489, A:A490, A:P491, A:K492, A:P493, A:E494, A:Q495, A:P496, A:A497, A:P498, A:A499, A:P500, A:K501, A:P502, A:E503, A:Q504, A:P505, A:A506, A:K510, A:P511, A:A512, A:E513, A:E514, A:P515, A:T516, A:Q517, A:P518, A:E519, A:K520, A:P521, A:A522, A:T523, A:P524, A:K525, A:T526 | 83 | 0.794 |
|  | 2 | A:M1, A:N2, A:K3, A:K4, A:K5, A:M6, A:I7, A:L8, A:T9, A:S10, A:L11, A:A12, A:S13, A:V14, A:A15, A:I16, A:L17, A:G18, A:A19, A:G20, A:L21, A:V22, A:T23, A:S24, A:Q25, A:P26, A:T27, A:F28, A:V29, A:R30, A:A31, A:E32, A:E33, A:S34, A:P35, A:Q36, A:V37, A:V38, A:E39, A:K40, A:S41, A:S42, A:L43, A:E44, A:K45, A:K46, A:Y47, A:E48, A:E49, A:A50, A:K51, A:A52, A:K53, A:A54, A:D55, A:A57, A:K58, A:K59, A:D60, A:E62, A:T63, A:A64, A:K65, A:K66, A:K67, A:A68, A:E69, A:D70, A:A71, A:Q72, A:K73, A:K74, A:Y75, A:E76, A:D77, A:D78, A:Q79, A:R81, A:K95, A:D98, A:V99, A:A100, A:L101, A:V102, A:V103, A:Q104, A:N105, A:A106, A:Y107, A:K108, A:Y110 | 91 | 0.77 |
|  | 3 | A:N337, A:K338, A:L339, A:A340, A:A341, A:A344, A:E345, A:A347, A:K348, A:K349, A:Q350, A:T351, A:E352, A:E354, A:K355, A:L356, A:D358, A:S359, A:D361, A:P362, A:E363, A:G364, A:K365, A:T366, A:Q367, A:D371, A:A374, A:E375, A:A377, A:E378, A:L379, A:D380, A:K381, A:K382, A:A383, A:D384, A:E385, A:L386, A:Q387, A:N388, A:K389, A:V390, A:A391, A:D392, A:L393, A:E394, A:K395, A:E396, A:I397, A:N399, A:L400, A:L403, A:L404 | 53 | 0.666 |
|  | 4 | A:H226, A:Q227, A:N230, A:L234 | 4 | 0.568 |
|  | 5 | A:E132, A:S135, A:K136, A:E138, A:K139, A:K142, A:E143, A:P160, A:P162, A:N163, A:A166, A:E167, A:K169, A:K170, A:A172, A:E173, A:E174, A:A175, A:K176, A:A177, A:E178, A:A202, A:L205, A:E206, A:I207, A:E208 | 26 | 0.566 |
|  | 6 | A:V193, A:A194, A:L195, A:K198, A:E201 | 5 | 0.559 |
|  | 7 | A:E410, A:D412, A:T413, A:A414, A:A415, A:L416, A:Q417, A:N418, A:K419, A:L420, A:A421, A:K423 | 12 | 0.554 |
|  | 8 | A:E109, A:R111, A:A140, A:R141, A:Q144 | 5 | 0.548 |
| Clade 4 | 1 | A:T556, A:G557, A:W558, A:L559, A:Q560, A:Y561, A:N562, A:G563, A:S564, A:W565, A:Y566, A:Y567, A:M574, A:A575, A:T576, A:G577, A:W578, A:A579, A:K580, A:V581, A:H582, A:G583, A:S584, A:W585, A:Y586, A:Y587, A:L588, A:N589, A:A590, A:N591, A:G592, A:S593, A:M594, A:A595, A:T596, A:G597, A:W598, A:V599, A:K600, A:D601, A:G602, A:E603, A:T604, A:W605, A:Y606, A:Y607, A:L608, A:E609, A:A610, A:S611, A:G612, A:S613, A:M614, A:K615, A:A616, A:N617, A:Q618, A:W619, A:F620, A:Q621, A:V622, A:S623, A:D624, A:K625, A:W626, A:Y627, A:Y628, A:V629, A:N630, A:G631, A:L632, A:G633, A:S634, A:L635, A:S636, A:V637, A:N638, A:T639, A:T640, A:V641, A:D642, A:G643, A:Y644, A:K645, A:V646, A:N647, A:A648, A:N649, A:G650, A:E651, A:W652, A:V653 | 92 | 0.852 |
|  | 2 | A:I228, A:A229, A:R230, A:L231, A:Q232, A:S233, A:D234, A:L235, A:K236, A:D237, A:A238, A:E239, A:E240, A:N241, A:N242, A:V243, A:E244, A:D245, A:Y246, A:I247, A:K248 | 21 | 0.751 |
|  | 3 | A:V182, A:K186, A:I189, A:L190, A:Q192, A:D193, A:A194, A:E195, A:N196, A:E197, A:K198, A:K199, A:I200, A:D201, A:V202, A:L203, A:Q204, A:N205, A:K206, A:V207, A:A208, A:D209, A:L210, A:E211, A:K212, A:G213, A:I214, A:A215, A:P216, A:N219, A:N225 | 31 | 0.741 |
|  | 4 | A:A121, A:D122, A:K123, A:K124, A:I125, A:K126, A:E127, A:A128, A:T129, A:Q130, A:R131, A:K132, A:D133, A:E134, A:A135, A:E136, A:A137, A:A138, A:F139, A:A140, A:T141, A:I142, A:R143, A:T144, A:T145, A:I146, A:V147, A:V148, A:P149, A:E150, A:P151, A:S152, A:E153, A:L154, A:A155, A:E156, A:T157, A:K158, A:K159, A:K160, A:A161, A:E162, A:E163, A:A164, A:T165, A:K166, A:E167, A:A168, A:E169, A:V170, A:K173, A:K174, A:E176 | 53 | 0.722 |
|  | 5 | A:D75, A:E76, A:K79 | 3 | 0.671 |
|  | 6 | A:E282, A:E314, A:A315, A:L316, A:Q317, A:N318, A:Q319, A:E326, A:L327, A:S328, A:K329, A:L330, A:E331, A:D332, A:N333, A:L334, A:K335, A:D336, A:A337, A:E338, A:T339, A:N340, A:N341, A:V342, A:E343, A:D344, A:E348, A:E352, A:A353, A:I354, A:A355, A:T356, A:K357, A:K358, A:A359, A:E360, A:L361, A:E362, A:K363, A:T364, A:Q365, A:K366, A:E367, A:L368, A:D369, A:A370, A:A371, A:P377, A:D378, A:G379, A:D380, A:E381, A:E383, A:T384, A:P385, A:A386, A:P387, A:A388, A:P389, A:Q390, A:P391, A:E392, A:K393, A:P394, A:A395, A:E396, A:E397, A:P398, A:E399, A:S410, A:A411, A:Q413, A:A415, A:E416, A:E417, A:D418, A:Y419, A:A420, A:R421, A:R422, A:S423, A:E424 | 82 | 0.667 |
|  | 7 | A:K65, A:E68, A:D69, A:K72 | 4 | 0.62 |
|  | 8 | A:V36, A:A37, A:N38, A:Q39, A:S40, A:A42, A:E43, A:K44 | 8 | 0.602 |
|  | 9 | A:N268, A:I269, A:D270, A:K271, A:T272, A:K274, A:D275 | 7 | 0.593 |
|  | 10 | A:P26, A:E33, A:L287, A:A288, A:T289, A:L290, A:D291, A:P292, A:E293, A:G294, A:K295, A:T296, A:Q297, A:D298, A:E299, A:L300, A:D301 | 17 | 0.589 |
|  | 11 | A:Y46, A:D47, A:V50, A:K51 | 4 | 0.575 |
| Clade 5 | 1 | A:E391, A:T392, A:P393, A:A394, A:P395, A:A396, A:P397, A:K398, A:P399, A:E400, A:Q401, A:P402, A:A403, A:E404, A:Q405, A:P406, A:K407, A:P408, A:A409, A:P410, A:A411, A:P412, A:K413, A:P414, A:E415, A:K416, A:T417, A:D418, A:D419, A:Q420, A:Q421, A:A422, A:E423, A:E424, A:D425, A:Y426, A:A427, A:R428, A:R429, A:S430, A:E431, A:E432, A:E433, A:Y434, A:N435, A:R436, A:L437, A:P438, A:Q439, A:Q440, A:Q441, A:P442, A:P443, A:K444, A:A445, A:E446, A:P448, A:E455, A:Q456, A:P457, A:V458, A:P459, A:A460, A:P461 | 64 | 0.799 |
|  | 2 | A:K3, A:K4, A:K5, A:M6, A:I7, A:L8, A:T9, A:S10, A:L11, A:A12, A:S13, A:V14, A:A15, A:I16, A:L17, A:G18, A:T19, A:G20, A:F21, A:V22, A:A23, A:S24, A:S25, A:P26, A:T27, A:F28, A:V29, A:R30, A:A31, A:E32, A:E33, A:S34, A:P35, A:Q36, A:V37, A:V38, A:E39, A:K40, A:S41, A:S42, A:L43, A:E44, A:K45, A:Y47, A:E48, A:E49, A:A50, A:K51, A:A52, A:K53, A:A54, A:D55, A:T56, A:A57, A:K58, A:Y61, A:Q72, A:Y75, A:D76, A:E77, A:D78, A:Q79, A:K80, A:K81, A:T82, A:E83, A:D84, A:K85, A:A86, A:K87, A:A88 | 71 | 0.771 |
|  | 3 | A:A302, A:D304, A:A305, A:N306, A:I307, A:E308, A:A309, A:Q311, A:N312, A:A315, A:D316, A:T329, A:R330, A:L331, A:Q332, A:S333, A:D334, A:L335, A:K336, A:D337, A:A338, A:E339, A:E340, A:N341, A:N342, A:V343, A:E344, A:D345, A:Y346, A:V347, A:K348, A:E349, A:G350, A:L351, A:K353, A:T356, A:D357, A:K358, A:K359, A:V360, A:E361, A:L362, A:N363, A:N364, A:T365, A:Q366, A:A368, A:L369 | 48 | 0.7 |
|  | 4 | A:V108, A:E109, A:E112, A:A113, A:K114, A:D115, A:K116, A:A117, A:S118, A:A119, A:K122 | 11 | 0.644 |
|  | 5 | A:E125, A:A126, A:R128, A:K129, A:Q130, A:E132, A:A133, A:N134, A:K135, A:D164, A:A165, A:E166, A:V167 | 13 | 0.542 |

Table S5: Predicted helper T-cell epitopes for PspA Proteins using IEDB server (Percentile Rank ≤20).

| CDR  Antigen (PspA) | HLA-DR  B10101 | HLA-DR  B10301 | HLA-DR  B10401 | HLA-DR  B10801 | H2-IAd | H2-IAb | H2-IEd |
| --- | --- | --- | --- | --- | --- | --- | --- |
| Clade1 | - | LQFELDVKQAKLSKL  PLQFELDVKQAKLSK  APLQFELDVKQAKLS  RAPLQFELDVKQAKL  LRAPLQFELDVKQAK  QFELDVKQAKLSKLE  FELDVKQAKLSKLEE  IAKLEKDVEDFKNSD  AKLEKDVEDFKNSDG  AEIAKLEKDVEDFKN  EIAKLEKDVEDFKNS  DAEIAKLEKDVEDFK | - | AAAEEDLVAKKAELE  AAEEDLVAKKAELEK  AEEDLVAKKAELEKT  DLVAKKAELEKTEAD  EDLVAKKAELEKTEA  EEDLVAKKAELEKTE  LVAKKAELEKTEADL | QFELDVKQAKLSKLE  FELDVKQAKLSKLEE | - | PLQFELDVKQAKLSK |
| Clade2 | - | - | - | - | EGLEKTIAAKKAELE  GLEKTIAAKKAELEK  KEGLEKTIAAKKAEL  FKEGLEKTIAAKKAE  LEKTIAAKKAELEKT | YAKEGFRAPLQSKLD  DYAKEGFRAPLQSKL  AKEGFRAPLQSKLDA  KEGFRAPLQSKLDAK | - |
| Clade3 | - | - | ADLEKEISNLEILLG | ADELQNKVADLEKEI  DELQNKVADLEKEIS  DKKADELQNKVADLE  ELQNKVADLEKEISN  KADELQNKVADLEKE  KKADELQNKVADLEK  LQNKVADLEKEISNL | AALQNKLATKKAELE  TAALQNKLATKKAEL | - | - |
| Clade4 | EKVEALQNQVAELEE  NEKVEALQNQVAELE  LNEKVEALQNQVAEL  ELNEKVEALQNQVAE | LELEKVLATLDPEGK  ELELEKVLATLDPEG | - | - | EGLEEAIATKKAELE  GLEEAIATKKAELEK  KEGLEEAIATKKAEL  DAELELEKVLATLDP  AELELEKVLATLDPE  EDAELELEKVLATLD  IKEGLEEAIATKKAE | - | - |
| Clade5 | ANIEALQNKVADLEN  DANIEALQNKVADLE  EDANIEALQNKVADL  AEDANIEALQNKVAD | VTRLQSDLKDAEENN  EVTRLQSDLKDAEEN  KEVTRLQSDLKDAEE  TRLQSDLKDAEENNV  DKEVTRLQSDLKDAE  RLQSDLKDAEENNVE  LQSDLKDAEENNVED  EGLEKALTDKKVELN  GLEKALTDKKVELNN  LEKALTDKKVELNNT  EKALTDKKVELNNTQ  KEGLEKALTDKKVEL | LELEKVLATLDPEGK  ELELEKVLATLDPEG | ALQNKVADLENKVAE  ANIEALQNKVADLEN  DANIEALQNKVADLE  EALQNKVADLENKVA  IEALQNKVADLENKV  LQNKVADLENKVAEL  NIEALQNKVADLENK | DAELELEKVLATLDP  AELELEKVLATLDPE  EDAELELEKVLATLD | TQKALDTAPKALDTA  LNNTQKALDTAPKAL  NTQKALDTAPKALDT  NNTQKALDTAPKALD  QKALDTAPKALDTAL | - |

Table S6: Predicted helper T-cell epitopes for PspA proteins using RANKPEP server.

| CDR Antigen (PspA) | HLA-DR  B10101 | HLA-DR  B10301 | HLA-DR  B10401 | HLA-DR  B10701 | HLA-DR  B11101 | H2-IAd | H2-IAb | H2-IEd |
| --- | --- | --- | --- | --- | --- | --- | --- | --- |
| Clade1 | YLAAAEEDL | FELDVKQAK | YVKEGLRAP  YLAAAEEDL | - | - | - | AGQYLAAAE  EDLVAKKAE | LDVKQAKLS |
| Clade2 | - | - | AKLEDQLKA  YFKEGLEKT | - | YAKEGFRAP | - | KTIAAKKAE  EGLEKTIAA | - |
| Clade3 | TAALQNKLA | - | AALQNKLAT  ADSEDDTAA | - | - | - | AELEKTQKE  LQNKVADLE | TAALQNKLA |
| Clade4 | VEALQNQVA | - | YIKEGLEEA | - | - | EAIATKKAE  EALQNQVAE | LKDAETNNV | - |
| Clade5 | IEALQNKVA | - | YVKEGLEKA  VELNNTQKA  TRLQSDLKD | YVKEGLEKA | - | LQNKVADLE | - | KVELNNTQK  DYVKEGLEK  IEALQNKVA |

Table S7: Predicted helper T-cell epitopes for PspA Proteins with IC50 value ≤100 (nM) using MHCPred server.

| PspA Clades | HLA-DR  B10101 | HLA-DR  B10401 | HLA-DR  B10701 | H2-IAd | H2-IAb |
| --- | --- | --- | --- | --- | --- |
| Clade1 | [ELDVKQAKL](http://www.ddg-pharmfac.net/mhcpred/scripts/MHCPred_scripts/additive.pl), [KIDELDAEI](http://www.ddg-pharmfac.net/mhcpred/scripts/MHCPred_scripts/additive.pl), [YVKEGLRAP](http://www.ddg-pharmfac.net/mhcpred/scripts/MHCPred_scripts/additive.pl), [ELDAEIAKL](http://www.ddg-pharmfac.net/mhcpred/scripts/MHCPred_scripts/additive.pl), [QYLAAAEED](http://www.ddg-pharmfac.net/mhcpred/scripts/MHCPred_scripts/additive.pl), [DLVAKKAEL](http://www.ddg-pharmfac.net/mhcpred/scripts/MHCPred_scripts/additive.pl), [ELSDKIDEL](http://www.ddg-pharmfac.net/mhcpred/scripts/MHCPred_scripts/additive.pl), [QAKLSKLEE](http://www.ddg-pharmfac.net/mhcpred/scripts/MHCPred_scripts/additive.pl), [KLEELSDKI](http://www.ddg-pharmfac.net/mhcpred/scripts/MHCPred_scripts/additive.pl), [EIDESDSED](http://www.ddg-pharmfac.net/mhcpred/scripts/MHCPred_scripts/additive.pl), [LEKTEADLK](http://www.ddg-pharmfac.net/mhcpred/scripts/MHCPred_scripts/additive.pl), [DVEDFKNSD](http://www.ddg-pharmfac.net/mhcpred/scripts/MHCPred_scripts/additive.pl), [EEDLVAKKA](http://www.ddg-pharmfac.net/mhcpred/scripts/MHCPred_scripts/additive.pl), [KTEADLKKA](http://www.ddg-pharmfac.net/mhcpred/scripts/MHCPred_scripts/additive.pl), [IAKLEKDVE](http://www.ddg-pharmfac.net/mhcpred/scripts/MHCPred_scripts/additive.pl), [EADLKKAVN](http://www.ddg-pharmfac.net/mhcpred/scripts/MHCPred_scripts/additive.pl), [GQYLAAAEE](http://www.ddg-pharmfac.net/mhcpred/scripts/MHCPred_scripts/additive.pl), [QFELDVKQA](http://www.ddg-pharmfac.net/mhcpred/scripts/MHCPred_scripts/additive.pl), [KDVEDFKNS](http://www.ddg-pharmfac.net/mhcpred/scripts/MHCPred_scripts/additive.pl), [PLQFELDVK](http://www.ddg-pharmfac.net/mhcpred/scripts/MHCPred_scripts/additive.pl), [DYVKEGLRA](http://www.ddg-pharmfac.net/mhcpred/scripts/MHCPred_scripts/additive.pl), [IDELDAEIA](http://www.ddg-pharmfac.net/mhcpred/scripts/MHCPred_scripts/additive.pl), [SDKIDELDA](http://www.ddg-pharmfac.net/mhcpred/scripts/MHCPred_scripts/additive.pl), [SDGEQAGQY](http://www.ddg-pharmfac.net/mhcpred/scripts/MHCPred_scripts/additive.pl), [LDVKQAKLS](http://www.ddg-pharmfac.net/mhcpred/scripts/MHCPred_scripts/additive.pl), [DVKQAKLSK](http://www.ddg-pharmfac.net/mhcpred/scripts/MHCPred_scripts/additive.pl), [KAELEKTEA](http://www.ddg-pharmfac.net/mhcpred/scripts/MHCPred_scripts/additive.pl), [QAGQYLAAA](http://www.ddg-pharmfac.net/mhcpred/scripts/MHCPred_scripts/additive.pl), [EGLRAPLQF](http://www.ddg-pharmfac.net/mhcpred/scripts/MHCPred_scripts/additive.pl), [GLRAPLQFE](http://www.ddg-pharmfac.net/mhcpred/scripts/MHCPred_scripts/additive.pl), [EDYVKEGLR](http://www.ddg-pharmfac.net/mhcpred/scripts/MHCPred_scripts/additive.pl), [LSKLEELSD](http://www.ddg-pharmfac.net/mhcpred/scripts/MHCPred_scripts/additive.pl), [KEGLRAPLQ](http://www.ddg-pharmfac.net/mhcpred/scripts/MHCPred_scripts/additive.pl), [ELEKTEADL](http://www.ddg-pharmfac.net/mhcpred/scripts/MHCPred_scripts/additive.pl), [KALKEIDES](http://www.ddg-pharmfac.net/mhcpred/scripts/MHCPred_scripts/additive.pl), [NSDGEQAGQ](http://www.ddg-pharmfac.net/mhcpred/scripts/MHCPred_scripts/additive.pl), [IDESDSEDY](http://www.ddg-pharmfac.net/mhcpred/scripts/MHCPred_scripts/additive.pl), [LQFELDVKQ](http://www.ddg-pharmfac.net/mhcpred/scripts/MHCPred_scripts/additive.pl), [DFKNSDGEQ](http://www.ddg-pharmfac.net/mhcpred/scripts/MHCPred_scripts/additive.pl), [FELDVKQAK](http://www.ddg-pharmfac.net/mhcpred/scripts/MHCPred_scripts/additive.pl), [AGQYLAAAE](http://www.ddg-pharmfac.net/mhcpred/scripts/MHCPred_scripts/additive.pl), [ADLKKAVNE](http://www.ddg-pharmfac.net/mhcpred/scripts/MHCPred_scripts/additive.pl), [LRAPLQFEL](http://www.ddg-pharmfac.net/mhcpred/scripts/MHCPred_scripts/additive.pl) | [VKQAKLSKL](http://www.ddg-pharmfac.net/mhcpred/scripts/MHCPred_scripts/additive.pl)  [GEQAGQYLA](http://www.ddg-pharmfac.net/mhcpred/scripts/MHCPred_scripts/additive.pl)  [QYLAAAEED](http://www.ddg-pharmfac.net/mhcpred/scripts/MHCPred_scripts/additive.pl)  [DVKQAKLSK](http://www.ddg-pharmfac.net/mhcpred/scripts/MHCPred_scripts/additive.pl) | [EEDLVAKKA](http://www.ddg-pharmfac.net/mhcpred/scripts/MHCPred_scripts/additive.pl)  [KKAELEKTE](http://www.ddg-pharmfac.net/mhcpred/scripts/MHCPred_scripts/additive.pl)  [KDVEDFKNS](http://www.ddg-pharmfac.net/mhcpred/scripts/MHCPred_scripts/additive.pl)  [EIAKLEKDV](http://www.ddg-pharmfac.net/mhcpred/scripts/MHCPred_scripts/additive.pl) | [QAGQYLAAA](http://www.ddg-pharmfac.net/mhcpred/scripts/MHCPred_scripts/additive.pl)  [LAAAEEDLV](http://www.ddg-pharmfac.net/mhcpred/scripts/MHCPred_scripts/additive.pl)  [TEADLKKAV](http://www.ddg-pharmfac.net/mhcpred/scripts/MHCPred_scripts/additive.pl)  [AAAEEDLVA](http://www.ddg-pharmfac.net/mhcpred/scripts/MHCPred_scripts/additive.pl)  [GEQAGQYLA](http://www.ddg-pharmfac.net/mhcpred/scripts/MHCPred_scripts/additive.pl)  [AGQYLAAAE](http://www.ddg-pharmfac.net/mhcpred/scripts/MHCPred_scripts/additive.pl) | [YVKEGLRAP](http://www.ddg-pharmfac.net/mhcpred/scripts/MHCPred_scripts/additive.pl)  [LRAPLQFEL](http://www.ddg-pharmfac.net/mhcpred/scripts/MHCPred_scripts/additive.pl)  [TEADLKKAV](http://www.ddg-pharmfac.net/mhcpred/scripts/MHCPred_scripts/additive.pl) |
| Clade2 | [QSKLDAKKA](http://www.ddg-pharmfac.net/mhcpred/scripts/MHCPred_scripts/additive.pl), [KIDELDAEI](http://www.ddg-pharmfac.net/mhcpred/scripts/MHCPred_scripts/additive.pl), [KLDAKKAKL](http://www.ddg-pharmfac.net/mhcpred/scripts/MHCPred_scripts/additive.pl), [ELDAEIAKL](http://www.ddg-pharmfac.net/mhcpred/scripts/MHCPred_scripts/additive.pl), [FRAPLQSKL](http://www.ddg-pharmfac.net/mhcpred/scripts/MHCPred_scripts/additive.pl), [EDQLKAAEE](http://www.ddg-pharmfac.net/mhcpred/scripts/MHCPred_scripts/additive.pl), [LEKTIAAKK](http://www.ddg-pharmfac.net/mhcpred/scripts/MHCPred_scripts/additive.pl), [ELSDKIDEL](http://www.ddg-pharmfac.net/mhcpred/scripts/MHCPred_scripts/additive.pl), [YAKEGFRAP](http://www.ddg-pharmfac.net/mhcpred/scripts/MHCPred_scripts/additive.pl), [KLEELSDKI](http://www.ddg-pharmfac.net/mhcpred/scripts/MHCPred_scripts/additive.pl), [LEKTEADLK](http://www.ddg-pharmfac.net/mhcpred/scripts/MHCPred_scripts/additive.pl), [KLEDQLKAA](http://www.ddg-pharmfac.net/mhcpred/scripts/MHCPred_scripts/additive.pl), [PLQSKLDAK](http://www.ddg-pharmfac.net/mhcpred/scripts/MHCPred_scripts/additive.pl), [KAKLSKLEE](http://www.ddg-pharmfac.net/mhcpred/scripts/MHCPred_scripts/additive.pl), [KTEADLKKA](http://www.ddg-pharmfac.net/mhcpred/scripts/MHCPred_scripts/additive.pl), [EIDESESED](http://www.ddg-pharmfac.net/mhcpred/scripts/MHCPred_scripts/additive.pl), [EADLKKAVN](http://www.ddg-pharmfac.net/mhcpred/scripts/MHCPred_scripts/additive.pl), [YFKEGLEKT](http://www.ddg-pharmfac.net/mhcpred/scripts/MHCPred_scripts/additive.pl), [IDELDAEIA](http://www.ddg-pharmfac.net/mhcpred/scripts/MHCPred_scripts/additive.pl), [TIAAKKAEL](http://www.ddg-pharmfac.net/mhcpred/scripts/MHCPred_scripts/additive.pl), [SDKIDELDA](http://www.ddg-pharmfac.net/mhcpred/scripts/MHCPred_scripts/additive.pl), [EGFRAPLQS](http://www.ddg-pharmfac.net/mhcpred/scripts/MHCPred_scripts/additive.pl), [LEDQLKAAE](http://www.ddg-pharmfac.net/mhcpred/scripts/MHCPred_scripts/additive.pl), [AKKAKLSKL](http://www.ddg-pharmfac.net/mhcpred/scripts/MHCPred_scripts/additive.pl), [IAKLEDQLK](http://www.ddg-pharmfac.net/mhcpred/scripts/MHCPred_scripts/additive.pl), [KAELEKTEA](http://www.ddg-pharmfac.net/mhcpred/scripts/MHCPred_scripts/additive.pl), [LSKLEELSD](http://www.ddg-pharmfac.net/mhcpred/scripts/MHCPred_scripts/additive.pl), [QLKAAEENN](http://www.ddg-pharmfac.net/mhcpred/scripts/MHCPred_scripts/additive.pl), [EKTIAAKKA](http://www.ddg-pharmfac.net/mhcpred/scripts/MHCPred_scripts/additive.pl), [ELEKTEADL](http://www.ddg-pharmfac.net/mhcpred/scripts/MHCPred_scripts/additive.pl), [EDYAKEGFR](http://www.ddg-pharmfac.net/mhcpred/scripts/MHCPred_scripts/additive.pl), [KEGFRAPLQ](http://www.ddg-pharmfac.net/mhcpred/scripts/MHCPred_scripts/additive.pl), [DAEIAKLED](http://www.ddg-pharmfac.net/mhcpred/scripts/MHCPred_scripts/additive.pl), [IDESESEDY](http://www.ddg-pharmfac.net/mhcpred/scripts/MHCPred_scripts/additive.pl), [KLSKLEELS](http://www.ddg-pharmfac.net/mhcpred/scripts/MHCPred_scripts/additive.pl) | - | [QSKLDAKKA](http://www.ddg-pharmfac.net/mhcpred/scripts/MHCPred_scripts/additive.pl)  [KKAELEKTE](http://www.ddg-pharmfac.net/mhcpred/scripts/MHCPred_scripts/additive.pl)  [EENNNVEDY](http://www.ddg-pharmfac.net/mhcpred/scripts/MHCPred_scripts/additive.pl) | [TEADLKKAV](http://www.ddg-pharmfac.net/mhcpred/scripts/MHCPred_scripts/additive.pl)  [KKAKLSKLE](http://www.ddg-pharmfac.net/mhcpred/scripts/MHCPred_scripts/additive.pl) | [KAAEENNNV](http://www.ddg-pharmfac.net/mhcpred/scripts/MHCPred_scripts/additive.pl)  [TEADLKKAV](http://www.ddg-pharmfac.net/mhcpred/scripts/MHCPred_scripts/additive.pl)  [KEGFRAPLQ](http://www.ddg-pharmfac.net/mhcpred/scripts/MHCPred_scripts/additive.pl)  [KLEDQLKAA](http://www.ddg-pharmfac.net/mhcpred/scripts/MHCPred_scripts/additive.pl)  [RAPLQSKLD](http://www.ddg-pharmfac.net/mhcpred/scripts/MHCPred_scripts/additive.pl)  [EGLEKTIAA](http://www.ddg-pharmfac.net/mhcpred/scripts/MHCPred_scripts/additive.pl) |
| Clade3 | [SEDDTAALQ](http://www.ddg-pharmfac.net/mhcpred/scripts/MHCPred_scripts/additive.pl), [ELDAALNEL](http://www.ddg-pharmfac.net/mhcpred/scripts/MHCPred_scripts/additive.pl), [KADELQNKV](http://www.ddg-pharmfac.net/mhcpred/scripts/MHCPred_scripts/additive.pl), [KELDAALNE](http://www.ddg-pharmfac.net/mhcpred/scripts/MHCPred_scripts/additive.pl), [EDDTAALQN](http://www.ddg-pharmfac.net/mhcpred/scripts/MHCPred_scripts/additive.pl), [ELDKKADEL](http://www.ddg-pharmfac.net/mhcpred/scripts/MHCPred_scripts/additive.pl), [KLLDSLDPE](http://www.ddg-pharmfac.net/mhcpred/scripts/MHCPred_scripts/additive.pl), [TQKELDAAL](http://www.ddg-pharmfac.net/mhcpred/scripts/MHCPred_scripts/additive.pl), [LEKLLDSLD](http://www.ddg-pharmfac.net/mhcpred/scripts/MHCPred_scripts/additive.pl), [DDTAALQNK](http://www.ddg-pharmfac.net/mhcpred/scripts/MHCPred_scripts/additive.pl), [ELQNKVADL](http://www.ddg-pharmfac.net/mhcpred/scripts/MHCPred_scripts/additive.pl), [LEKEISNLE](http://www.ddg-pharmfac.net/mhcpred/scripts/MHCPred_scripts/additive.pl), [EISNLEILL](http://www.ddg-pharmfac.net/mhcpred/scripts/MHCPred_scripts/additive.pl), [KTQDELDKE](http://www.ddg-pharmfac.net/mhcpred/scripts/MHCPred_scripts/additive.pl), [DTAALQNKL](http://www.ddg-pharmfac.net/mhcpred/scripts/MHCPred_scripts/additive.pl), [QNKLATKKA](http://www.ddg-pharmfac.net/mhcpred/scripts/MHCPred_scripts/additive.pl), [QNKVADLEK](http://www.ddg-pharmfac.net/mhcpred/scripts/MHCPred_scripts/additive.pl), [TQDELDKEA](http://www.ddg-pharmfac.net/mhcpred/scripts/MHCPred_scripts/additive.pl), [ELDKEAEEA](http://www.ddg-pharmfac.net/mhcpred/scripts/MHCPred_scripts/additive.pl), [LATKKAELE](http://www.ddg-pharmfac.net/mhcpred/scripts/MHCPred_scripts/additive.pl), [DLEKEISNL](http://www.ddg-pharmfac.net/mhcpred/scripts/MHCPred_scripts/additive.pl), [NLEILLGGA](http://www.ddg-pharmfac.net/mhcpred/scripts/MHCPred_scripts/additive.pl), [DSEDDTAAL](http://www.ddg-pharmfac.net/mhcpred/scripts/MHCPred_scripts/additive.pl), [DELDKEAEE](http://www.ddg-pharmfac.net/mhcpred/scripts/MHCPred_scripts/additive.pl), [ILLGGADSE](http://www.ddg-pharmfac.net/mhcpred/scripts/MHCPred_scripts/additive.pl), [QKELDAALN](http://www.ddg-pharmfac.net/mhcpred/scripts/MHCPred_scripts/additive.pl), [KLATKKAEL](http://www.ddg-pharmfac.net/mhcpred/scripts/MHCPred_scripts/additive.pl), [EKTQKELDA](http://www.ddg-pharmfac.net/mhcpred/scripts/MHCPred_scripts/additive.pl), [EILLGGADS](http://www.ddg-pharmfac.net/mhcpred/scripts/MHCPred_scripts/additive.pl), [ELEKTQKEL](http://www.ddg-pharmfac.net/mhcpred/scripts/MHCPred_scripts/additive.pl), [ISNLEILLG](http://www.ddg-pharmfac.net/mhcpred/scripts/MHCPred_scripts/additive.pl), [EKLLDSLDP](http://www.ddg-pharmfac.net/mhcpred/scripts/MHCPred_scripts/additive.pl), [KTQKELDAA](http://www.ddg-pharmfac.net/mhcpred/scripts/MHCPred_scripts/additive.pl), [NKVADLEKE](http://www.ddg-pharmfac.net/mhcpred/scripts/MHCPred_scripts/additive.pl), [EAELDKKAD](http://www.ddg-pharmfac.net/mhcpred/scripts/MHCPred_scripts/additive.pl) | [DELQNKVAD](http://www.ddg-pharmfac.net/mhcpred/scripts/MHCPred_scripts/additive.pl)  [NKLATKKAE](http://www.ddg-pharmfac.net/mhcpred/scripts/MHCPred_scripts/additive.pl)  [AALQNKLAT](http://www.ddg-pharmfac.net/mhcpred/scripts/MHCPred_scripts/additive.pl) | [EISNLEILL](http://www.ddg-pharmfac.net/mhcpred/scripts/MHCPred_scripts/additive.pl)  [KADELQNKV](http://www.ddg-pharmfac.net/mhcpred/scripts/MHCPred_scripts/additive.pl)  [NKLATKKAE](http://www.ddg-pharmfac.net/mhcpred/scripts/MHCPred_scripts/additive.pl)  [KKAELEKTQ](http://www.ddg-pharmfac.net/mhcpred/scripts/MHCPred_scripts/additive.pl) | [KVADLEKEI](http://www.ddg-pharmfac.net/mhcpred/scripts/MHCPred_scripts/additive.pl) | [TAALQNKLA](http://www.ddg-pharmfac.net/mhcpred/scripts/MHCPred_scripts/additive.pl)  [TQKELDAAL](http://www.ddg-pharmfac.net/mhcpred/scripts/MHCPred_scripts/additive.pl)  [KADELQNKV](http://www.ddg-pharmfac.net/mhcpred/scripts/MHCPred_scripts/additive.pl)  [GADSEDDTA](http://www.ddg-pharmfac.net/mhcpred/scripts/MHCPred_scripts/additive.pl)  [SEDDTAALQ](http://www.ddg-pharmfac.net/mhcpred/scripts/MHCPred_scripts/additive.pl)  [KTQKELDAA](http://www.ddg-pharmfac.net/mhcpred/scripts/MHCPred_scripts/additive.pl)  [AALNELGPD](http://www.ddg-pharmfac.net/mhcpred/scripts/MHCPred_scripts/additive.pl)  [KEISNLEIL](http://www.ddg-pharmfac.net/mhcpred/scripts/MHCPred_scripts/additive.pl)  [AALQNKLAT](http://www.ddg-pharmfac.net/mhcpred/scripts/MHCPred_scripts/additive.pl)  [TQDELDKEA](http://www.ddg-pharmfac.net/mhcpred/scripts/MHCPred_scripts/additive.pl) |
| Clade4 | [KVEALQNQV](http://www.ddg-pharmfac.net/mhcpred/scripts/MHCPred_scripts/additive.pl), [KELDAALNE](http://www.ddg-pharmfac.net/mhcpred/scripts/MHCPred_scripts/additive.pl), [KVLATLDPE](http://www.ddg-pharmfac.net/mhcpred/scripts/MHCPred_scripts/additive.pl), [NEKVEALQN](http://www.ddg-pharmfac.net/mhcpred/scripts/MHCPred_scripts/additive.pl), [LEKVLATLD](http://www.ddg-pharmfac.net/mhcpred/scripts/MHCPred_scripts/additive.pl), [YIKEGLEEA](http://www.ddg-pharmfac.net/mhcpred/scripts/MHCPred_scripts/additive.pl), [ELDKEAAEA](http://www.ddg-pharmfac.net/mhcpred/scripts/MHCPred_scripts/additive.pl), [ELEEELSKL](http://www.ddg-pharmfac.net/mhcpred/scripts/MHCPred_scripts/additive.pl), [TQKELDAAL](http://www.ddg-pharmfac.net/mhcpred/scripts/MHCPred_scripts/additive.pl), [IATKKAELE](http://www.ddg-pharmfac.net/mhcpred/scripts/MHCPred_scripts/additive.pl), [KTQDELDKE](http://www.ddg-pharmfac.net/mhcpred/scripts/MHCPred_scripts/additive.pl), [KLEDNLKDA](http://www.ddg-pharmfac.net/mhcpred/scripts/MHCPred_scripts/additive.pl), [ELELEKVLA](http://www.ddg-pharmfac.net/mhcpred/scripts/MHCPred_scripts/additive.pl), [NLKDAETNN](http://www.ddg-pharmfac.net/mhcpred/scripts/MHCPred_scripts/additive.pl), [LEEAIATKK](http://www.ddg-pharmfac.net/mhcpred/scripts/MHCPred_scripts/additive.pl), [TQDELDKEA](http://www.ddg-pharmfac.net/mhcpred/scripts/MHCPred_scripts/additive.pl), [QNQVAELEE](http://www.ddg-pharmfac.net/mhcpred/scripts/MHCPred_scripts/additive.pl), [LSKLEDNLK](http://www.ddg-pharmfac.net/mhcpred/scripts/MHCPred_scripts/additive.pl), [LDKEAAEAE](http://www.ddg-pharmfac.net/mhcpred/scripts/MHCPred_scripts/additive.pl), [NQVAELEEE](http://www.ddg-pharmfac.net/mhcpred/scripts/MHCPred_scripts/additive.pl), [LEDNLKDAE](http://www.ddg-pharmfac.net/mhcpred/scripts/MHCPred_scripts/additive.pl), [DELDKEAAE](http://www.ddg-pharmfac.net/mhcpred/scripts/MHCPred_scripts/additive.pl), [QKELDAALN](http://www.ddg-pharmfac.net/mhcpred/scripts/MHCPred_scripts/additive.pl), [EAIATKKAE](http://www.ddg-pharmfac.net/mhcpred/scripts/MHCPred_scripts/additive.pl), [LEDAELELE](http://www.ddg-pharmfac.net/mhcpred/scripts/MHCPred_scripts/additive.pl), [DAELELEKV](http://www.ddg-pharmfac.net/mhcpred/scripts/MHCPred_scripts/additive.pl), [AAEAELNEK](http://www.ddg-pharmfac.net/mhcpred/scripts/MHCPred_scripts/additive.pl), [EEELSKLED](http://www.ddg-pharmfac.net/mhcpred/scripts/MHCPred_scripts/additive.pl), [LKDAETNNV](http://www.ddg-pharmfac.net/mhcpred/scripts/MHCPred_scripts/additive.pl), [ELSKLEDNL](http://www.ddg-pharmfac.net/mhcpred/scripts/MHCPred_scripts/additive.pl), [EKTQKELDA](http://www.ddg-pharmfac.net/mhcpred/scripts/MHCPred_scripts/additive.pl), [QDELDKEAA](http://www.ddg-pharmfac.net/mhcpred/scripts/MHCPred_scripts/additive.pl), [ELEKTQKEL](http://www.ddg-pharmfac.net/mhcpred/scripts/MHCPred_scripts/additive.pl), [LELEKVLAT](http://www.ddg-pharmfac.net/mhcpred/scripts/MHCPred_scripts/additive.pl), [KTQKELDAA](http://www.ddg-pharmfac.net/mhcpred/scripts/MHCPred_scripts/additive.pl), [EKVLATLDP](http://www.ddg-pharmfac.net/mhcpred/scripts/MHCPred_scripts/additive.pl), [EALQNQVAE](http://www.ddg-pharmfac.net/mhcpred/scripts/MHCPred_scripts/additive.pl), [EKVEALQNQ](http://www.ddg-pharmfac.net/mhcpred/scripts/MHCPred_scripts/additive.pl) | [KVLATLDPE](http://www.ddg-pharmfac.net/mhcpred/scripts/MHCPred_scripts/additive.pl)  [EALQNQVAE](http://www.ddg-pharmfac.net/mhcpred/scripts/MHCPred_scripts/additive.pl)  [EEAIATKKA](http://www.ddg-pharmfac.net/mhcpred/scripts/MHCPred_scripts/additive.pl) | [EAIATKKAE](http://www.ddg-pharmfac.net/mhcpred/scripts/MHCPred_scripts/additive.pl)  [EALQNQVAE](http://www.ddg-pharmfac.net/mhcpred/scripts/MHCPred_scripts/additive.pl)  [EAELNEKVE](http://www.ddg-pharmfac.net/mhcpred/scripts/MHCPred_scripts/additive.pl)  [EGLEEAIAT](http://www.ddg-pharmfac.net/mhcpred/scripts/MHCPred_scripts/additive.pl)  [KKAELEKTQ](http://www.ddg-pharmfac.net/mhcpred/scripts/MHCPred_scripts/additive.pl) | [IKEGLEEAI](http://www.ddg-pharmfac.net/mhcpred/scripts/MHCPred_scripts/additive.pl) | [TQKELDAAL](http://www.ddg-pharmfac.net/mhcpred/scripts/MHCPred_scripts/additive.pl)  [KEGLEEAIA](http://www.ddg-pharmfac.net/mhcpred/scripts/MHCPred_scripts/additive.pl)  [AEAELNEKV](http://www.ddg-pharmfac.net/mhcpred/scripts/MHCPred_scripts/additive.pl)  [ALQNQVAEL](http://www.ddg-pharmfac.net/mhcpred/scripts/MHCPred_scripts/additive.pl)  [EGLEEAIAT](http://www.ddg-pharmfac.net/mhcpred/scripts/MHCPred_scripts/additive.pl)  [KTQKELDAA](http://www.ddg-pharmfac.net/mhcpred/scripts/MHCPred_scripts/additive.pl)  [LNEKVEALQ](http://www.ddg-pharmfac.net/mhcpred/scripts/MHCPred_scripts/additive.pl)  [TLDPEGKTQ](http://www.ddg-pharmfac.net/mhcpred/scripts/MHCPred_scripts/additive.pl)  [LELEKVLAT](http://www.ddg-pharmfac.net/mhcpred/scripts/MHCPred_scripts/additive.pl)  [VEALQNQVA](http://www.ddg-pharmfac.net/mhcpred/scripts/MHCPred_scripts/additive.pl)  [TQDELDKEA](http://www.ddg-pharmfac.net/mhcpred/scripts/MHCPred_scripts/additive.pl) |
| Clade5 | [NIEALQNKV](http://www.ddg-pharmfac.net/mhcpred/scripts/MHCPred_scripts/additive.pl), [KALDTALNE](http://www.ddg-pharmfac.net/mhcpred/scripts/MHCPred_scripts/additive.pl), [KALDTAPKA](http://www.ddg-pharmfac.net/mhcpred/scripts/MHCPred_scripts/additive.pl), [EVTRLQSDL](http://www.ddg-pharmfac.net/mhcpred/scripts/MHCPred_scripts/additive.pl), [KVLATLDPE](http://www.ddg-pharmfac.net/mhcpred/scripts/MHCPred_scripts/additive.pl), [NNTQKALDT](http://www.ddg-pharmfac.net/mhcpred/scripts/MHCPred_scripts/additive.pl), [LEKVLATLD](http://www.ddg-pharmfac.net/mhcpred/scripts/MHCPred_scripts/additive.pl), [YVKEGLEKA](http://www.ddg-pharmfac.net/mhcpred/scripts/MHCPred_scripts/additive.pl), [ALDTALNEL](http://www.ddg-pharmfac.net/mhcpred/scripts/MHCPred_scripts/additive.pl), [TQKALDTAP](http://www.ddg-pharmfac.net/mhcpred/scripts/MHCPred_scripts/additive.pl)  [QSDLKDAEE](http://www.ddg-pharmfac.net/mhcpred/scripts/MHCPred_scripts/additive.pl), [ELDKEAAED](http://www.ddg-pharmfac.net/mhcpred/scripts/MHCPred_scripts/additive.pl), [RLQSDLKDA](http://www.ddg-pharmfac.net/mhcpred/scripts/MHCPred_scripts/additive.pl), [KTQDELDKE](http://www.ddg-pharmfac.net/mhcpred/scripts/MHCPred_scripts/additive.pl), [DKEVTRLQS](http://www.ddg-pharmfac.net/mhcpred/scripts/MHCPred_scripts/additive.pl), [DLENKVAEL](http://www.ddg-pharmfac.net/mhcpred/scripts/MHCPred_scripts/additive.pl), [LDKEAAEDA](http://www.ddg-pharmfac.net/mhcpred/scripts/MHCPred_scripts/additive.pl), [LDTAPKALD](http://www.ddg-pharmfac.net/mhcpred/scripts/MHCPred_scripts/additive.pl), [QNKVADLEN](http://www.ddg-pharmfac.net/mhcpred/scripts/MHCPred_scripts/additive.pl), [KVADLENKV](http://www.ddg-pharmfac.net/mhcpred/scripts/MHCPred_scripts/additive.pl)  [KEVTRLQSD](http://www.ddg-pharmfac.net/mhcpred/scripts/MHCPred_scripts/additive.pl), [ELELEKVLA](http://www.ddg-pharmfac.net/mhcpred/scripts/MHCPred_scripts/additive.pl), [ALTDKKVEL](http://www.ddg-pharmfac.net/mhcpred/scripts/MHCPred_scripts/additive.pl), [TQDELDKEA](http://www.ddg-pharmfac.net/mhcpred/scripts/MHCPred_scripts/additive.pl), [DKKVELNNT](http://www.ddg-pharmfac.net/mhcpred/scripts/MHCPred_scripts/additive.pl), [TALNELGPD](http://www.ddg-pharmfac.net/mhcpred/scripts/MHCPred_scripts/additive.pl), [DTAPKALDT](http://www.ddg-pharmfac.net/mhcpred/scripts/MHCPred_scripts/additive.pl), [LEKALTDKK](http://www.ddg-pharmfac.net/mhcpred/scripts/MHCPred_scripts/additive.pl), [DANIEALQN](http://www.ddg-pharmfac.net/mhcpred/scripts/MHCPred_scripts/additive.pl), [ELNNTQKAL](http://www.ddg-pharmfac.net/mhcpred/scripts/MHCPred_scripts/additive.pl), [EGLEKALTD](http://www.ddg-pharmfac.net/mhcpred/scripts/MHCPred_scripts/additive.pl), [DELDKEAAE](http://www.ddg-pharmfac.net/mhcpred/scripts/MHCPred_scripts/additive.pl), [LQSDLKDAE](http://www.ddg-pharmfac.net/mhcpred/scripts/MHCPred_scripts/additive.pl), [NKVAELDKE](http://www.ddg-pharmfac.net/mhcpred/scripts/MHCPred_scripts/additive.pl), [EAAEDANIE](http://www.ddg-pharmfac.net/mhcpred/scripts/MHCPred_scripts/additive.pl)  [LEDAELELE](http://www.ddg-pharmfac.net/mhcpred/scripts/MHCPred_scripts/additive.pl), [DAELELEKV](http://www.ddg-pharmfac.net/mhcpred/scripts/MHCPred_scripts/additive.pl), [ENKVAELDK](http://www.ddg-pharmfac.net/mhcpred/scripts/MHCPred_scripts/additive.pl), [KALTDKKVE](http://www.ddg-pharmfac.net/mhcpred/scripts/MHCPred_scripts/additive.pl), [ELDKEVTRL](http://www.ddg-pharmfac.net/mhcpred/scripts/MHCPred_scripts/additive.pl)  [ALQNKVADL](http://www.ddg-pharmfac.net/mhcpred/scripts/MHCPred_scripts/additive.pl), [TRLQSDLKD](http://www.ddg-pharmfac.net/mhcpred/scripts/MHCPred_scripts/additive.pl), [LKDAEENNV](http://www.ddg-pharmfac.net/mhcpred/scripts/MHCPred_scripts/additive.pl), [QDELDKEAA](http://www.ddg-pharmfac.net/mhcpred/scripts/MHCPred_scripts/additive.pl), [EDYVKEGLE](http://www.ddg-pharmfac.net/mhcpred/scripts/MHCPred_scripts/additive.pl), [LELEKVLAT](http://www.ddg-pharmfac.net/mhcpred/scripts/MHCPred_scripts/additive.pl), [LNNTQKALD](http://www.ddg-pharmfac.net/mhcpred/scripts/MHCPred_scripts/additive.pl), [DLKDAEENN](http://www.ddg-pharmfac.net/mhcpred/scripts/MHCPred_scripts/additive.pl), [EKVLATLDP](http://www.ddg-pharmfac.net/mhcpred/scripts/MHCPred_scripts/additive.pl) | [KVLATLDPE](http://www.ddg-pharmfac.net/mhcpred/scripts/MHCPred_scripts/additive.pl)  [VELNNTQKA](http://www.ddg-pharmfac.net/mhcpred/scripts/MHCPred_scripts/additive.pl)  [DKEAAEDAN](http://www.ddg-pharmfac.net/mhcpred/scripts/MHCPred_scripts/additive.pl)  [NTQKALDTA](http://www.ddg-pharmfac.net/mhcpred/scripts/MHCPred_scripts/additive.pl)  [EALQNKVAD](http://www.ddg-pharmfac.net/mhcpred/scripts/MHCPred_scripts/additive.pl) | [KALDTAPKA](http://www.ddg-pharmfac.net/mhcpred/scripts/MHCPred_scripts/additive.pl)  [KALDTALNE](http://www.ddg-pharmfac.net/mhcpred/scripts/MHCPred_scripts/additive.pl)  [EKALTDKKV](http://www.ddg-pharmfac.net/mhcpred/scripts/MHCPred_scripts/additive.pl)  [EALQNKVAD](http://www.ddg-pharmfac.net/mhcpred/scripts/MHCPred_scripts/additive.pl)  [KVADLENKV](http://www.ddg-pharmfac.net/mhcpred/scripts/MHCPred_scripts/additive.pl)  [KALTDKKVE](http://www.ddg-pharmfac.net/mhcpred/scripts/MHCPred_scripts/additive.pl)  [LKDAEENNV](http://www.ddg-pharmfac.net/mhcpred/scripts/MHCPred_scripts/additive.pl) | [VKEGLEKAL](http://www.ddg-pharmfac.net/mhcpred/scripts/MHCPred_scripts/additive.pl)  [EKALTDKKV](http://www.ddg-pharmfac.net/mhcpred/scripts/MHCPred_scripts/additive.pl)  [EKVLATLDP](http://www.ddg-pharmfac.net/mhcpred/scripts/MHCPred_scripts/additive.pl) | [KEGLEKALT](http://www.ddg-pharmfac.net/mhcpred/scripts/MHCPred_scripts/additive.pl)  [TALNELGPD](http://www.ddg-pharmfac.net/mhcpred/scripts/MHCPred_scripts/additive.pl)  [TAPKALDTA](http://www.ddg-pharmfac.net/mhcpred/scripts/MHCPred_scripts/additive.pl)  [KVADLENKV](http://www.ddg-pharmfac.net/mhcpred/scripts/MHCPred_scripts/additive.pl)  [KVAELDKEV](http://www.ddg-pharmfac.net/mhcpred/scripts/MHCPred_scripts/additive.pl)  [TQKALDTAP](http://www.ddg-pharmfac.net/mhcpred/scripts/MHCPred_scripts/additive.pl)  [PKALDTALN](http://www.ddg-pharmfac.net/mhcpred/scripts/MHCPred_scripts/additive.pl)  [TLDPEGKTQ](http://www.ddg-pharmfac.net/mhcpred/scripts/MHCPred_scripts/additive.pl)  [LELEKVLAT](http://www.ddg-pharmfac.net/mhcpred/scripts/MHCPred_scripts/additive.pl)  [AAEDANIEA](http://www.ddg-pharmfac.net/mhcpred/scripts/MHCPred_scripts/additive.pl)  [TQDELDKEA](http://www.ddg-pharmfac.net/mhcpred/scripts/MHCPred_scripts/additive.pl) |

Table S8. The best IL4 inducing analog/peptide from PspA_1-5c+p_ construct

| [**Peptide Sequence**](https://webs.iiitd.edu.in/raghava/il4pred/prot_submitfreq_S.php?ran=43495) | [**SVM score**](https://webs.iiitd.edu.in/raghava/il4pred/prot_submitfreq_S.php?ran=43495) | [**Prediction**](https://webs.iiitd.edu.in/raghava/il4pred/prot_submitfreq_S.php?ran=43495) | [**Hydrophobicity**](https://webs.iiitd.edu.in/raghava/il4pred/prot_submitfreq_S.php?ran=43495) | [**Hydropathicity**](https://webs.iiitd.edu.in/raghava/il4pred/prot_submitfreq_S.php?ran=43495) | [**Hydrophilicity**](https://webs.iiitd.edu.in/raghava/il4pred/prot_submitfreq_S.php?ran=43495) | [**Charge**](https://webs.iiitd.edu.in/raghava/il4pred/prot_submitfreq_S.php?ran=43495) | [**Mol wt**](https://webs.iiitd.edu.in/raghava/il4pred/prot_submitfreq_S.php?ran=43495) |
| --- | --- | --- | --- | --- | --- | --- | --- |
| [MGHHHHHHLEKALKE](https://webs.iiitd.edu.in/raghava/il4pred/pepsearch_S.php?seq=MGHHHHHHLEKALKE&thval=0.2) | 0.28 | IL4 inducer | -0.27 | -1.54 | 0.24 | 3.00 | 1841.33 |
| [HLEKALKEIDESDSE](https://webs.iiitd.edu.in/raghava/il4pred/pepsearch_S.php?seq=HLEKALKEIDESDSE&thval=0.2) | 0.31 | IL4 inducer | -0.33 | -1.31 | 1.21 | -3.50 | 1743.07 |
| [EIDESDSEDYVKEGL](https://webs.iiitd.edu.in/raghava/il4pred/pepsearch_S.php?seq=EIDESDSEDYVKEGL&thval=0.2) | 0.32 | IL4 inducer | -0.29 | -1.28 | 1.15 | -6.00 | 1727.96 |
| [EDYVKEGLRAPLQFE](https://webs.iiitd.edu.in/raghava/il4pred/pepsearch_S.php?seq=EDYVKEGLRAPLQFE&thval=0.2) | 0.27 | IL4 inducer | -0.24 | -0.85 | 0.52 | -2.00 | 1794.21 |
| [LRAPLQFELDVKQAK](https://webs.iiitd.edu.in/raghava/il4pred/pepsearch_S.php?seq=LRAPLQFELDVKQAK&thval=0.2) | 0.23 | IL4 inducer | -0.23 | -0.39 | 0.33 | 1.00 | 1756.30 |
| [FELDVKQAKLSKLEE](https://webs.iiitd.edu.in/raghava/il4pred/pepsearch_S.php?seq=FELDVKQAKLSKLEE&thval=0.2) | 0.24 | IL4 inducer | -0.26 | -0.65 | 0.77 | -1.00 | 1777.27 |
| [KQAKLSKLEELSDKI](https://webs.iiitd.edu.in/raghava/il4pred/pepsearch_S.php?seq=KQAKLSKLEELSDKI&thval=0.2) | 0.23 | IL4 inducer | -0.33 | -0.90 | 0.94 | 1.00 | 1730.26 |
| [ELSDKIDELDAEIAK](https://webs.iiitd.edu.in/raghava/il4pred/pepsearch_S.php?seq=ELSDKIDELDAEIAK&thval=0.2) | 0.23 | IL4 inducer | -0.23 | -0.63 | 1.07 | -4.00 | 1689.06 |
| [KIDELDAEIAKLEKD](https://webs.iiitd.edu.in/raghava/il4pred/pepsearch_S.php?seq=KIDELDAEIAKLEKD&thval=0.2) | 0.45 | IL4 inducer | -0.29 | -0.83 | 1.25 | -3.00 | 1730.16 |
| [DAEIAKLEKDVEDFK](https://webs.iiitd.edu.in/raghava/il4pred/pepsearch_S.php?seq=DAEIAKLEKDVEDFK&thval=0.2) | 0.21 | IL4 inducer | -0.29 | -0.92 | 1.23 | -3.00 | 1750.14 |
| [AEIAKLEKDVEDFKN](https://webs.iiitd.edu.in/raghava/il4pred/pepsearch_S.php?seq=AEIAKLEKDVEDFKN&thval=0.2) | 0.22 | IL4 inducer | -0.29 | -0.92 | 1.04 | -2.00 | 1749.16 |
| [DVEDFKNSDGEQAGQ](https://webs.iiitd.edu.in/raghava/il4pred/pepsearch_S.php?seq=DVEDFKNSDGEQAGQ&thval=0.2) | 0.34 | IL4 inducer | -0.34 | -1.65 | 0.96 | -4.00 | 1638.83 |
| [VEDFKNSDGEQAGQY](https://webs.iiitd.edu.in/raghava/il4pred/pepsearch_S.php?seq=VEDFKNSDGEQAGQY&thval=0.2) | 0.30 | IL4 inducer | -0.29 | -1.50 | 0.61 | -3.00 | 1686.92 |
| [GEQAGQYLAAAEEDL](https://webs.iiitd.edu.in/raghava/il4pred/pepsearch_S.php?seq=GEQAGQYLAAAEEDL&thval=0.2) | 0.21 | IL4 inducer | -0.10 | -0.55 | 0.30 | -4.00 | 1564.84 |
| [KKAELEKTEADLKKA](https://webs.iiitd.edu.in/raghava/il4pred/pepsearch_S.php?seq=KKAELEKTEADLKKA&thval=0.2) | 0.36 | IL4 inducer | -0.43 | -1.41 | 1.43 | 1.00 | 1702.19 |
| [KAELEKTEADLKKAV](https://webs.iiitd.edu.in/raghava/il4pred/pepsearch_S.php?seq=KAELEKTEADLKKAV&thval=0.2) | 0.34 | IL4 inducer | -0.32 | -0.87 | 1.13 | 0.00 | 1673.15 |
| [ADLKKAVNEEAAAKL](https://webs.iiitd.edu.in/raghava/il4pred/pepsearch_S.php?seq=ADLKKAVNEEAAAKL&thval=0.2) | 0.24 | IL4 inducer | -0.20 | -0.33 | 0.71 | 0.00 | 1571.01 |
| [KKAVNEEAAAKLKEI](https://webs.iiitd.edu.in/raghava/il4pred/pepsearch_S.php?seq=KKAVNEEAAAKLKEI&thval=0.2) | 0.27 | IL4 inducer | -0.27 | -0.66 | 0.94 | 1.00 | 1642.14 |
| [VNEEAAAKLKEIDES](https://webs.iiitd.edu.in/raghava/il4pred/pepsearch_S.php?seq=VNEEAAAKLKEIDES&thval=0.2) | 0.24 | IL4 inducer | -0.25 | -0.78 | 0.99 | -3.00 | 1645.99 |
| [EEAAAKLKEIDESES](https://webs.iiitd.edu.in/raghava/il4pred/pepsearch_S.php?seq=EEAAAKLKEIDESES&thval=0.2) | 0.42 | IL4 inducer | -0.30 | -1.11 | 1.30 | -4.00 | 1648.94 |
| [LKEIDESESEDYAKE](https://webs.iiitd.edu.in/raghava/il4pred/pepsearch_S.php?seq=LKEIDESESEDYAKE&thval=0.2) | 0.57 | IL4 inducer | -0.38 | -1.67 | 1.41 | -5.00 | 1785.05 |
| [IDESESEDYAKEGFR](https://webs.iiitd.edu.in/raghava/il4pred/pepsearch_S.php?seq=IDESESEDYAKEGFR&thval=0.2) | 0.25 | IL4 inducer | -0.37 | -1.57 | 1.17 | -4.00 | 1775.01 |
| [KKAKLSKLEELSDKI](https://webs.iiitd.edu.in/raghava/il4pred/pepsearch_S.php?seq=KKAKLSKLEELSDKI&thval=0.2) | 0.24 | IL4 inducer | -0.36 | -0.93 | 1.13 | 2.00 | 1730.30 |
| [ELSDKIDELDAEIAK](https://webs.iiitd.edu.in/raghava/il4pred/pepsearch_S.php?seq=ELSDKIDELDAEIAK&thval=0.2) | 0.23 | IL4 inducer | -0.23 | -0.63 | 1.07 | -4.00 | 1689.06 |
| [LSDKIDELDAEIAKL](https://webs.iiitd.edu.in/raghava/il4pred/pepsearch_S.php?seq=LSDKIDELDAEIAKL&thval=0.2) | 0.29 | IL4 inducer | -0.15 | -0.14 | 0.75 | -3.00 | 1673.11 |
| [KIDELDAEIAKLEDQ](https://webs.iiitd.edu.in/raghava/il4pred/pepsearch_S.php?seq=KIDELDAEIAKLEDQ&thval=0.2) | 0.31 | IL4 inducer | -0.26 | -0.81 | 1.07 | -4.00 | 1730.12 |
| [LKAAEENNNVEDYFK](https://webs.iiitd.edu.in/raghava/il4pred/pepsearch_S.php?seq=LKAAEENNNVEDYFK&thval=0.2) | 0.21 | IL4 inducer | -0.30 | -1.28 | 0.63 | -2.00 | 1784.12 |
| [KAAEENNNVEDYFKE](https://webs.iiitd.edu.in/raghava/il4pred/pepsearch_S.php?seq=KAAEENNNVEDYFKE&thval=0.2) | 0.23 | IL4 inducer | -0.38 | -1.77 | 0.95 | -3.00 | 1800.07 |
| [AAEENNNVEDYFKEG](https://webs.iiitd.edu.in/raghava/il4pred/pepsearch_S.php?seq=AAEENNNVEDYFKEG&thval=0.2) | 0.22 | IL4 inducer | -0.29 | -1.53 | 0.75 | -4.00 | 1728.95 |
| [AEENNNVEDYFKEGL](https://webs.iiitd.edu.in/raghava/il4pred/pepsearch_S.php?seq=AEENNNVEDYFKEGL&thval=0.2) | 0.22 | IL4 inducer | -0.27 | -1.40 | 0.67 | -4.00 | 1771.04 |
| [KKAELEKTEADLKKA](https://webs.iiitd.edu.in/raghava/il4pred/pepsearch_S.php?seq=KKAELEKTEADLKKA&thval=0.2) | 0.36 | IL4 inducer | -0.43 | -1.41 | 1.43 | 1.00 | 1702.19 |
| [KAELEKTEADLKKAV](https://webs.iiitd.edu.in/raghava/il4pred/pepsearch_S.php?seq=KAELEKTEADLKKAV&thval=0.2) | 0.34 | IL4 inducer | -0.32 | -0.87 | 1.13 | 0.00 | 1673.15 |
| [EADLKKAVNEAAAKG](https://webs.iiitd.edu.in/raghava/il4pred/pepsearch_S.php?seq=EADLKKAVNEAAAKG&thval=0.2) | 0.26 | IL4 inducer | -0.23 | -0.61 | 0.83 | 0.00 | 1514.90 |
| [KGSASMPKPAPAQQA](https://webs.iiitd.edu.in/raghava/il4pred/pepsearch_S.php?seq=KGSASMPKPAPAQQA&thval=0.2) | 0.26 | IL4 inducer | -0.19 | -0.83 | 0.25 | 2.00 | 1468.89 |
| [GSASMPKPAPAQQAE](https://webs.iiitd.edu.in/raghava/il4pred/pepsearch_S.php?seq=GSASMPKPAPAQQAE&thval=0.2) | 0.34 | IL4 inducer | -0.16 | -0.81 | 0.25 | 0.00 | 1469.83 |
| [SASMPKPAPAQQAEE](https://webs.iiitd.edu.in/raghava/il4pred/pepsearch_S.php?seq=SASMPKPAPAQQAEE&thval=0.2) | 0.62 | IL4 inducer | -0.21 | -1.01 | 0.45 | -1.00 | 1541.89 |
| [PKPAPAQQAEEDYAR](https://webs.iiitd.edu.in/raghava/il4pred/pepsearch_S.php?seq=PKPAPAQQAEEDYAR&thval=0.2) | 0.32 | IL4 inducer | -0.36 | -1.65 | 0.74 | -1.00 | 1670.99 |
| [RLTQQQPKPEQPAPA](https://webs.iiitd.edu.in/raghava/il4pred/pepsearch_S.php?seq=RLTQQQPKPEQPAPA&thval=0.2) | 0.37 | IL4 inducer | -0.38 | -1.71 | 0.44 | 1.00 | 1689.12 |
| [LTQQQPKPEQPAPAP](https://webs.iiitd.edu.in/raghava/il4pred/pepsearch_S.php?seq=LTQQQPKPEQPAPAP&thval=0.2) | 0.37 | IL4 inducer | -0.27 | -1.51 | 0.24 | 0.00 | 1630.05 |
| [PKPEQPAPAPEKPAE](https://webs.iiitd.edu.in/raghava/il4pred/pepsearch_S.php?seq=PKPEQPAPAPEKPAE&thval=0.2) | 0.49 | IL4 inducer | -0.29 | -1.73 | 0.91 | -1.00 | 1585.97 |
| [KPEQPAPAPEKPAEK](https://webs.iiitd.edu.in/raghava/il4pred/pepsearch_S.php?seq=KPEQPAPAPEKPAEK&thval=0.2) | 0.47 | IL4 inducer | -0.36 | -1.89 | 1.11 | 0.00 | 1617.03 |
| [EQPAPAPEKPAEKPA](https://webs.iiitd.edu.in/raghava/il4pred/pepsearch_S.php?seq=EQPAPAPEKPAEKPA&thval=0.2) | 0.34 | IL4 inducer | -0.27 | -1.51 | 0.88 | -1.00 | 1559.93 |
| [APEKPAEKPAPAVDA](https://webs.iiitd.edu.in/raghava/il4pred/pepsearch_S.php?seq=APEKPAEKPAPAVDA&thval=0.2) | 0.20 | IL4 inducer | -0.18 | -0.77 | 0.73 | -1.00 | 1490.86 |
| [PEKPAEKPAPAVDAA](https://webs.iiitd.edu.in/raghava/il4pred/pepsearch_S.php?seq=PEKPAEKPAPAVDAA&thval=0.2) | 0.25 | IL4 inducer | -0.18 | -0.77 | 0.73 | -1.00 | 1490.86 |
| [AEKPAPAVDAAAEAA](https://webs.iiitd.edu.in/raghava/il4pred/pepsearch_S.php?seq=AEKPAPAVDAAAEAA&thval=0.2) | 0.45 | IL4 inducer | -0.04 | 0.07 | 0.43 | -2.00 | 1381.68 |
| [EKPAPAVDAAAEAAA](https://webs.iiitd.edu.in/raghava/il4pred/pepsearch_S.php?seq=EKPAPAVDAAAEAAA&thval=0.2) | 0.45 | IL4 inducer | -0.04 | 0.07 | 0.43 | -2.00 | 1381.68 |
| [EEAELDKKADELQNK](https://webs.iiitd.edu.in/raghava/il4pred/pepsearch_S.php?seq=EEAELDKKADELQNK&thval=0.2) | 0.34 | IL4 inducer | -0.47 | -1.90 | 1.52 | -3.00 | 1760.10 |
| [DELQNKVADLEKEIS](https://webs.iiitd.edu.in/raghava/il4pred/pepsearch_S.php?seq=DELQNKVADLEKEIS&thval=0.2) | 0.71 | IL4 inducer | -0.30 | -1.00 | 0.95 | -3.00 | 1731.11 |
| [LEKEISNLEILLGGA](https://webs.iiitd.edu.in/raghava/il4pred/pepsearch_S.php?seq=LEKEISNLEILLGGA&thval=0.2) | 0.30 | IL4 inducer | 0.02 | 0.43 | 0.08 | -2.00 | 1599.10 |
| [EKEISNLEILLGGAD](https://webs.iiitd.edu.in/raghava/il4pred/pepsearch_S.php?seq=EKEISNLEILLGGAD&thval=0.2) | 0.28 | IL4 inducer | -0.06 | -0.05 | 0.40 | -3.00 | 1601.02 |
| [DTAALQNKLATKKAE](https://webs.iiitd.edu.in/raghava/il4pred/pepsearch_S.php?seq=DTAALQNKLATKKAE&thval=0.2) | 0.35 | IL4 inducer | -0.28 | -0.82 | 0.60 | 1.00 | 1602.03 |
| [TAALQNKLATKKAEL](https://webs.iiitd.edu.in/raghava/il4pred/pepsearch_S.php?seq=TAALQNKLATKKAEL&thval=0.2) | 0.50 | IL4 inducer | -0.20 | -0.33 | 0.28 | 2.00 | 1600.11 |
| [NKLATKKAELEKTQK](https://webs.iiitd.edu.in/raghava/il4pred/pepsearch_S.php?seq=NKLATKKAELEKTQK&thval=0.2) | 0.48 | IL4 inducer | -0.46 | -1.58 | 1.07 | 3.00 | 1730.26 |
| [TQKELDAALNELGPD](https://webs.iiitd.edu.in/raghava/il4pred/pepsearch_S.php?seq=TQKELDAALNELGPD&thval=0.2) | 0.26 | IL4 inducer | -0.21 | -0.84 | 0.57 | -3.00 | 1613.96 |
| [QKELDAALNELGPDG](https://webs.iiitd.edu.in/raghava/il4pred/pepsearch_S.php?seq=QKELDAALNELGPDG&thval=0.2) | 0.27 | IL4 inducer | -0.18 | -0.82 | 0.60 | -3.00 | 1569.91 |
| [KELDAALNELGPDGD](https://webs.iiitd.edu.in/raghava/il4pred/pepsearch_S.php?seq=KELDAALNELGPDGD&thval=0.2) | 0.27 | IL4 inducer | -0.19 | -0.82 | 0.79 | -4.00 | 1556.86 |
| [PDGDEEETEAAAKLE](https://webs.iiitd.edu.in/raghava/il4pred/pepsearch_S.php?seq=PDGDEEETEAAAKLE&thval=0.2) | 0.22 | IL4 inducer | -0.30 | -1.46 | 1.35 | -6.00 | 1603.81 |
| [TEAAAKLEDAELELE](https://webs.iiitd.edu.in/raghava/il4pred/pepsearch_S.php?seq=TEAAAKLEDAELELE&thval=0.2) | 0.26 | IL4 inducer | -0.17 | -0.47 | 0.88 | -5.00 | 1631.96 |
| [LEDAELELEKVLATL](https://webs.iiitd.edu.in/raghava/il4pred/pepsearch_S.php?seq=LEDAELELEKVLATL&thval=0.2) | 0.39 | IL4 inducer | -0.05 | 0.31 | 0.41 | -4.00 | 1686.16 |
| [LEKVLATLDPEGKTQ](https://webs.iiitd.edu.in/raghava/il4pred/pepsearch_S.php?seq=LEKVLATLDPEGKTQ&thval=0.2) | 0.31 | IL4 inducer | -0.18 | -0.52 | 0.47 | -1.00 | 1642.11 |
| [KEAAEAELNEKVEAL](https://webs.iiitd.edu.in/raghava/il4pred/pepsearch_S.php?seq=KEAAEAELNEKVEAL&thval=0.2) | 0.23 | IL4 inducer | -0.22 | -0.65 | 0.94 | -3.00 | 1644.02 |
| [ELNEKVEALQNQVAE](https://webs.iiitd.edu.in/raghava/il4pred/pepsearch_S.php?seq=ELNEKVEALQNQVAE&thval=0.2) | 0.29 | IL4 inducer | -0.24 | -0.82 | 0.55 | -3.00 | 1714.09 |
| [EALQNQVAELEEELS](https://webs.iiitd.edu.in/raghava/il4pred/pepsearch_S.php?seq=EALQNQVAELEEELS&thval=0.2) | 0.40 | IL4 inducer | -0.18 | -0.64 | 0.53 | -5.00 | 1702.03 |
| [ALQNQVAELEEELSK](https://webs.iiitd.edu.in/raghava/il4pred/pepsearch_S.php?seq=ALQNQVAELEEELSK&thval=0.2) | 0.39 | IL4 inducer | -0.22 | -0.67 | 0.53 | -3.00 | 1701.09 |
| [ELEEELSKLEDNLKD](https://webs.iiitd.edu.in/raghava/il4pred/pepsearch_S.php?seq=ELEEELSKLEDNLKD&thval=0.2) | 0.25 | IL4 inducer | -0.37 | -1.43 | 1.35 | -5.00 | 1804.16 |
| [EELSKLEDNLKDAET](https://webs.iiitd.edu.in/raghava/il4pred/pepsearch_S.php?seq=EELSKLEDNLKDAET&thval=0.2) | 0.20 | IL4 inducer | -0.36 | -1.37 | 1.21 | -4.00 | 1734.06 |
| [DYIKEGLEEAIATKK](https://webs.iiitd.edu.in/raghava/il4pred/pepsearch_S.php?seq=DYIKEGLEEAIATKK&thval=0.2) | 0.28 | IL4 inducer | -0.23 | -0.78 | 0.79 | -1.00 | 1708.16 |
| [YIKEGLEEAIATKKA](https://webs.iiitd.edu.in/raghava/il4pred/pepsearch_S.php?seq=YIKEGLEEAIATKKA&thval=0.2) | 0.28 | IL4 inducer | -0.16 | -0.43 | 0.56 | 0.00 | 1664.15 |
| [LEEAIATKKAELEKT](https://webs.iiitd.edu.in/raghava/il4pred/pepsearch_S.php?seq=LEEAIATKKAELEKT&thval=0.2) | 0.55 | IL4 inducer | -0.24 | -0.64 | 0.89 | -1.00 | 1674.14 |
| [IATKKAELEKTQKEL](https://webs.iiitd.edu.in/raghava/il4pred/pepsearch_S.php?seq=IATKKAELEKTQKEL&thval=0.2) | 0.27 | IL4 inducer | -0.33 | -1.02 | 0.93 | 1.00 | 1730.26 |
| [ATKKAELEKTQKELD](https://webs.iiitd.edu.in/raghava/il4pred/pepsearch_S.php?seq=ATKKAELEKTQKELD&thval=0.2) | 0.29 | IL4 inducer | -0.43 | -1.55 | 1.25 | 0.00 | 1732.18 |
| [ELEKTQKELDAALNE](https://webs.iiitd.edu.in/raghava/il4pred/pepsearch_S.php?seq=ELEKTQKELDAALNE&thval=0.2) | 0.29 | IL4 inducer | -0.32 | -1.20 | 0.97 | -3.00 | 1731.11 |
| [KEAAEAELNEKVEAL](https://webs.iiitd.edu.in/raghava/il4pred/pepsearch_S.php?seq=KEAAEAELNEKVEAL&thval=0.2) | 0.23 | IL4 inducer | -0.22 | -0.65 | 0.94 | -3.00 | 1644.02 |
| [EAAEAELNEKVEALQ](https://webs.iiitd.edu.in/raghava/il4pred/pepsearch_S.php?seq=EAAEAELNEKVEALQ&thval=0.2) | 0.24 | IL4 inducer | -0.20 | -0.63 | 0.75 | -4.00 | 1643.98 |
| [VEALQNQVAELEEEL](https://webs.iiitd.edu.in/raghava/il4pred/pepsearch_S.php?seq=VEALQNQVAELEEEL&thval=0.2) | 0.41 | IL4 inducer | -0.13 | -0.31 | 0.41 | -5.00 | 1714.09 |
| [ELEEELSKLEDNLKD](https://webs.iiitd.edu.in/raghava/il4pred/pepsearch_S.php?seq=ELEEELSKLEDNLKD&thval=0.2) | 0.25 | IL4 inducer | -0.37 | -1.43 | 1.35 | -5.00 | 1804.16 |
| [EELSKLEDNLKDAET](https://webs.iiitd.edu.in/raghava/il4pred/pepsearch_S.php?seq=EELSKLEDNLKDAET&thval=0.2) | 0.20 | IL4 inducer | -0.36 | -1.37 | 1.21 | -4.00 | 1734.06 |
| [DYIKEGLEEAIATKK](https://webs.iiitd.edu.in/raghava/il4pred/pepsearch_S.php?seq=DYIKEGLEEAIATKK&thval=0.2) | 0.28 | IL4 inducer | -0.23 | -0.78 | 0.79 | -1.00 | 1708.16 |
| [YIKEGLEEAIATKKA](https://webs.iiitd.edu.in/raghava/il4pred/pepsearch_S.php?seq=YIKEGLEEAIATKKA&thval=0.2) | 0.28 | IL4 inducer | -0.16 | -0.43 | 0.56 | 0.00 | 1664.15 |
| [LEEAIATKKAELEKT](https://webs.iiitd.edu.in/raghava/il4pred/pepsearch_S.php?seq=LEEAIATKKAELEKT&thval=0.2) | 0.55 | IL4 inducer | -0.24 | -0.64 | 0.89 | -1.00 | 1674.14 |
| [IATKKAELEKTQKEL](https://webs.iiitd.edu.in/raghava/il4pred/pepsearch_S.php?seq=IATKKAELEKTQKEL&thval=0.2) | 0.27 | IL4 inducer | -0.33 | -1.02 | 0.93 | 1.00 | 1730.26 |
| [ATKKAELEKTQKELD](https://webs.iiitd.edu.in/raghava/il4pred/pepsearch_S.php?seq=ATKKAELEKTQKELD&thval=0.2) | 0.29 | IL4 inducer | -0.43 | -1.55 | 1.25 | 0.00 | 1732.18 |
| [ELEKTQKELDAALNE](https://webs.iiitd.edu.in/raghava/il4pred/pepsearch_S.php?seq=ELEKTQKELDAALNE&thval=0.2) | 0.29 | IL4 inducer | -0.32 | -1.20 | 0.97 | -3.00 | 1731.11 |
